# Genome-wide discovery of CBL genes in *Nitraria tangutorum* Bobr. and functional analysis of *NtCBL1-1* under drought and salt stress

**DOI:** 10.48130/FR-2023-0028

**Published:** 2023-12-22

**Authors:** Liming Zhu, Jingxiang Wu, Mengjuan Li, Hao Fang, Jingbo Zhang, Yuchang Chen, Jinhui Chen, Tielong Cheng

**Affiliations:** 1 State Key Laboratory of Tree Genetics and Breeding, Co-Innovation Center for Sustainable Forestry in Southern China, Nanjing Forestry University, Nanjing 210037, China; 2 Key Laboratory of Forest Genetics and Biotechnology of Ministry of Education, Nanjing Forestry University, Nanjing 210037, China; 3 Experimental Center of Desert Forestry, Chinese Academy of Forestry, Dengkou 015200, China

**Keywords:** CBL, Nitraria tangutorum Bobr., Salt stress, Drought stress

## Abstract

Calcineurin B-like (CBL) proteins are a class of important Ca^2+^ receptors that play key roles in plant stress response. CBLs have been shown to participate in responses to abiotic stresses such as drought, salt, and cold in many plant species, including *Arabidopsis* and rice. However, little is known about their potential functions in the desert halophyte *Nitraria tangutorum*. Here, we have identified 11 CBL genes distributed across six chromosomes of *N. tangutorum* and categorized them into four groups through phylogenetic analysis. Synteny analysis showed that they have strong collinear relationships and have undergone purifying selection during their evolution. *NtCBL* promoter regions contain a variety of *cis*-acting elements related to hormone and environmental stress responses. Real-time quantitative PCR showed that the expression of *NtCBLs* differed significantly among various tissues and was upregulated by salt and drought stress. We chose *NtCBL1-1* for an in-depth functional characterization and observed that transgenic *Arabidopsis* plants expressing *NtCBL1-1* exhibited increased tolerance to both drought and salt stress. Compared to wild-type *Arabidopsis*, transgenic lines showed higher germination rates, slower chlorophyll degradation, more soluble proteins, and reduced accumulation of the oxidative stress marker malondialdehyde. These findings indicate that *NtCBL1-1* plays a significant role in responding to drought and salt stress, laying the foundation for further investigations into the functional mechanisms of *NtCBL* genes in *N. tangutorum*.

## Introduction

Plants encounter various abiotic stresses throughout their life. Salt, drought, cold, and other abiotic stresses adversely affect plant growth and development, sometimes even leading to plant death^[1]^. Over the course of evolution, plants have gradually developed various mechanisms to mitigate the damage caused by environmental stress^[2]^. These pathways encompass the calcium ion response mechanism, where external stimuli induce alterations in the concentration of free calcium ions within the cytoplasm. This shift is subsequently transduced into downstream signals, instigating a sequence of responses that empower plants to adapt to or resist changes in their external environment. Calcineurin B-like proteins (CBLs) are a family of Ca^2+^ receptors. The CBL protein was first identified in the model plant *Arabidopsis* and was named for its high homology to animal neuronal calcium sensors (NCS) and yeast calcineurin B (CNB)^[3]^.

As Ca^2+^ receptors, CBLs have a typical helix-loop-helix elongation factor hand (EF-hand) domain that binds calcium ions. Various numbers of CBL genes have been identified in plants, including 10 in *Arabidopsis*^[4]^, six in *Brassica napus*^[5]^, and eight in grape^[6]^. Plant CBLs typically interact with CBL-interacting protein kinases (CIPKs) to form CBL–CIPK complexes, which transmit the Ca^2+^ signal^[7]^. The CBL–CIPK signaling network mediates plant responses to abiotic stresses such as high salt, drought, ABA, and low temperature, and it has an important role in the maintenance of normal plant physiological activities^[8]^. For example, AtCBL4–AtCIPK24 phosphorylates the Na^+^/H^+^ antiporter AtNHX7 on the cell membrane under high-salt stress^[9]^, promoting efflux of excess Na^+^ to maintain an appropriate balance of Na^+^ inside and outside the cell. Both *CBL1* and *CBL9* target *CIPK23*, which plays a role in the regulation of potassium uptake and stomatal movement^[10,11]^. Overexpression of *AtCBL9* and *AtCIPK3* increased the tolerance of transgenic plants to exogenous ABA^[12]^. *CaCIPK3* and *CaCBL2* interact to improve drought tolerance in pepper^[13]^.

*Nitraria tangutorum* Bobr. (*N. tangutorum*) is a deciduous shrub from the Nitrariaceae that is endemic to China and widely distributed in arid, semi-arid, and saline desert areas of northwest China^[14]^. *N. tangutorum* shows strong resistance to salt and alkali drought and has a highly developed root network, enabling it to stabilize sand and act as an effective windbreak. It therefore has an important role in protecting and maintaining the balance of the ecological environment, particularly in desert, semi-desert, and salinized areas of China.

Currently, research on *N. tangutorum* predominantly centers on physiological and ecological aspects, with relatively few studies addressing gene function analysis, especially for stress-resistant gene families. This has hindered the progress of molecular biology research on *N. tangutorum*. The CBL gene family has been extensively demonstrated to play a role in responding to abiotic stresses in other plant species. Nevertheless, their distribution and functionality in the halophyte *N. tangutorum* have yet to be documented.

In this study, CBL family members in *N. tangutorum* were identified, and their basic physicochemical properties, phylogeny, and stress responses were characterized. Subsequently, a representative CBL gene, *NtCBL1-1*, was cloned, and the effects of its overexpression in *Arabidopsis* were analyzed. These findings offer insights into the evolution and biological functions of the CBL gene family in *N. tangutorum*, thus establishing a theoretical basis for further investigations into the mechanisms of abiotic stress resistance in this desert halophyte. Also, it provides valuable insights for improving the stress tolerance of other agricultural and forestry plants.

## Materials and methods

### Plant materials and abiotic stress treatments

*N. tangutorum* was collected in Dengkou County, Inner Mongolia in 2020. The fruit was subsequently extracted, dried, and stored in sandy soil at 4 °C for a vernalization period of 3 months. After vernalization, the seeds were germinated in a seedling tray, and the resulting seedlings were transplanted into nutrient-rich soil. They were then cultivated in a greenhouse with a temperature of 23 °C, under a light-dark cycle of 16 h of light and 8 h of darkness.

Stress experiments were conducted using 2-month-old seedlings with multiple replicating seedlings employed for each treatment. One group was exposed to salt stress at 500 mM NaCl, while another group was exposed to drought stress at 20% PEG 6000. During stress treatment, the corresponding salt or PEG solution was carefully added to the seedling culture medium several times until the new solution began to slowly permeate. Subsequently, seedlings were immersed in an equal concentration response solution to maintain consistent stress levels. For each stress condition (0, 1, 4, 8, or 24 h), whole seedlings were exposed, and three seedlings were sampled from each treatment at each time point. Following sampling, the seedlings were rapidly frozen in liquid nitrogen and stored at −80 °C for RNA extraction.

### Identification of CBL genes in *N. tangutorum*

*Arabidopsis* AtCBL protein sequences and rice (*Oryza sativa*) OsCBL protein sequences were downloaded from TAIR (www.arabidopsis.org/) and Rice Genome Annotation Project (http://rice.plantbiology.msu.edu/) databases. CBL sequences from rice and *Arabidopsis* were used as BLASTP search queries to identify potential CBL genes in the unpublished genome sequence of *N. tangutorum*. The candidate sequences were submitted to the SMART database for further confirmation (http://smart.embl-heidelberg.de/). The identified CBL genes were named according to their homologous to AtCBL genes. Cell-PLoc2.0 (www.csbio.sjtu.edu.cn/bioinf/Cell-PLoc-2/) was used to predict their subcellular localization, and ExPASy (https://web.expasy.org/) was used to calculate their isoelectric points (PIs) and molecular weights (MWs).

### Phylogenetic chromosomal location and synteny analysis

MAFFT was used with default parameters to construct a multiple alignment of CBL nucleotide sequences^[15]^. IQ-TREE^[16]^ was used to build a phylogenetic tree (iqtree -s -m MFP -b 1000 -nt auto), which was then visualized with iTOL^[17]^. Inter-chromosomal relationships among *N. tangutorum* CBLs were determined using TBtools, and inter-species synteny analysis was performed with the MCscan pipeline of JCVI^[18]^. Ka/Ks ratios between NtCBL gene pairs were calculated with KaKs_Calculator 2.0^[19]^.

### Gene structure and *cis* regulatory element analysis

Protein motifs in the CBLs were analyzed using MEME website tools (http://meme-suite.org/tools/meme); the motif length ranged from 10 to 50 amino acid residues, the maximum number of motifs identified was 50, and other parameters were set to default values. Gene structures were analyzed using TBtools^[20]^. The promoter sequence was derived from the genome annotations file (Supplemental File S1), covering a 3,000 bp region upstream of the CBL gene's initiation codon. This extracted promoter sequence was then submitted to the PlantCARE platform^[21]^ for the prediction of *cis*-acting elements. The results of this prediction were subsequently visualized using Adobe Illustrator.

### Expression analysis of CBL genes in *N. tangutorum*

Plant total RNA was extracted using the Easte Super Total RNA Extraction Kit (Promega, Shanghai, China). Subsequently, cDNA was synthesized using the HiScript III 1st Strand cDNA Synthesis Kit (Vazyme, Nanjing, China). Quantitative real-time PCR primers were designed using the NCBI Primer-BLAST tools (https://www.ncbi.nlm.nih.gov/tools/primer-blast/), and the primer details can be found in Supplemental Table S1. The quantitative PCR was performed using AceQ qPCR SYBR Green Master Mix (Vazyme, Nanjing, China). For each stress condition and time point, three biological replicates were analyzed. The relative expression levels were determined using the 2^−ΔΔCᴛ^ method as described by Livak & Schmi^[22]^.

### *NtCBL1-1* cloning and *Arabidopsis* transformation

Based on the gene family identification results, specific primers were designed using the reference CDS sequence of *NtCBL1-1* (Supplemental Table S2) for the cloning process. Following successful cloning, the *NtCBL1-1* fragment was incorporated into the *pBI121* overexpression vector utilizing *Xma*I and *Sac*I restriction sites. The resultant overexpression vector was then introduced into wild-type *Arabidopsis* (Col-0) plants using the Agrobacterium tumefaciens infection transgenic method, as previously reported by Clough & Bent^[23]^. Positive transgenic plants were selected and subsequently propagated to generate homozygous strains of the T3 generation, which were employed for stress resistance experiments.

### Analysis of salt and drought tolerance of transgenic *Arabidopsis*

To measure germination rate, seeds of wild-type and T3-generation transgenic *Arabidopsis* were sterilized and placed onto ½ Murashige and Skoog (MS) medium containing 0 or 150 mM NaCl or 300 mM mannitol. Fifty to 70 seeds of each transgenic line were arrayed on each petri dish, and there were three biological replicate plates. On day 7, the plates were photographed and the germination percentage calculated.

For phenotypic and physiological assessments, transgenic *Arabidopsis* plants were transferred from petri dishes to nutrient-rich soil after 10 d of initial growth and then cultivated for an additional 10 d. Plants with roughly uniform growth were selected and irrigated with 200 mM NaCl or 300 mM mannitol to simulate salt or drought stress. The amount of solution added to each pot was the same. Phenotypic changes were observed continuously; leaves were collected, snap-frozen in liquid nitrogen, and stored in a −80 °C freezer for physiological and biochemical measurements. Soluble protein and MDA were detected using commercial kits (Jiancheng Bioengineering, Nanjing, China). Chlorophyll content was determined by the method of Lichtenthaler & Wellburn^[24]^. In brief, the main veins were removed from fresh *Arabidopsis* leaves, and samples of leaf material (0.1 g) were weighed and placed into 10 mL centrifuge tubes. Extraction solution (1:1 absolute ethanol:acetone) was added to each centrifuge tube, and extraction was performed under dark conditions for 24 h, during which time the chlorophyll was completely dissolved by shaking 3–5 times. Each assay was performed with three biological replicates of each treatment and time point.

## Results

### Genome-wide discovery of *CBL* genes in *N. tangutorum*

By performing blastp analysis using candidate sequences from *Arabidopsis* and rice CBL proteins, combined with redundancy elimination and domain analysis, we successfully pinpointed 11 members of the CBL gene family in the entire genome of *N. tangutorum*. These genes were designated *NtCBL1-1* to *NtCBL10-2*, primarily based on their homology with AtCBLs.

Subsequently, basic information on these identified CBL genes was compiled. It was observed that these genes were unevenly distributed across six chromosomes. The amino acid lengths ranged from 213 to 321, with isoelectric points (pI) between 4.66 and 5.22. Their molecular weight (Mw) sizes ranged from 24.65 kDa to 36.84 kDa (Table 1). Furthermore, predictions of subcellular localization indicated a likelihood of expression on the plasma membrane for all identified members.

**Table 1:** Physicochemical properties of CBLs of *N.tangutorum*

Gene ID	Original ID	Locus	Length (aa)	MW (kDa)	PI	Subcellular localization prediction
*NtCBL1-1*	NITAA04G1075	Chr4A	213	24.48	4.66	Plasma membrane
*NtCBL1-2*	NITAB04G1197	Chr4B	213	24.48	4.66	Plasma membrane
*NtCBL3-1*	NITAB02G1010	Chr2B	252	29.06	5.05	Plasma membrane
*NtCBL3-2*	NITAA02G0832	Chr2A	226	26.10	4.82	Plasma membrane
*NtCBL4-1*	NITAA02G2027	Chr2A	213	24.65	5.25	Plasma membrane
*NtCBL4-2*	NITAA02G2024	Chr2A	213	24.72	5.17	Plasma membrane
*NtCBL4-3*	NITAB02G2334	Chr2B	213	24.69	5.24	Plasma membrane
*NtCBL8-1*	NITAB04G1638	Chr4B	321	36.84	5.22	Plasma membrane
*NtCBL8-2*	NITAA04G1516	Chr4A	226	26.19	5.01	Plasma membrane
*NtCBL10-1*	NITAB05G0933	Chr5B	267	30.71	4.88	Plasma membrane
*NtCBL10-2*	NITAA05G0858	Chr5A	275	31.59	4.94	Plasma membrane

### Phylogenetic and synteny investigation of the CBL gene family

**Figure 1:**
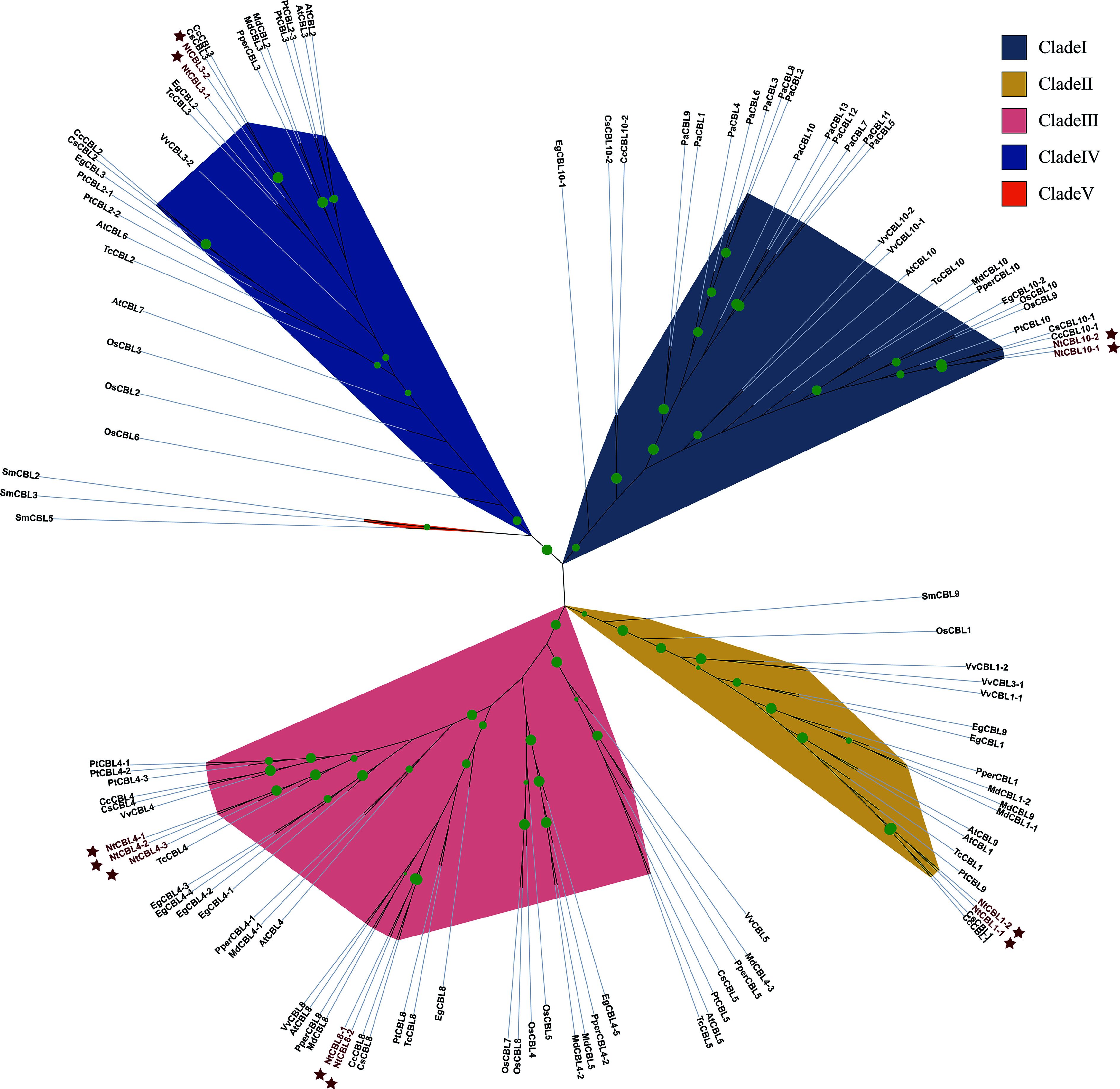
The phylogenetic tree shows the relationships between CBL protein sequences from *N. tangutorum* and 12 other species. Phylogenetic analysis was performed using IQ-TREE software with maximum likelihood (ML) method and subjected to 1,000 bootstrap replicates. The modules are color coded to represent the five subclades of CBLs. NtCBLs are indicated by dark red stars. Green dots of different sizes indicate Bootstraps confidence levels above 80.

To explore the phylogenetic relationships among CBLs, we constructed a maximum likelihood phylogenetic tree using 120 CBL sequences from 13 species (Supplemental Table S3), including *N. tangutorum*. Our analysis revealed that these CBL genes fall mainly into five distinct categories, with most genes clustered together based on their respective gene names. Clade 5 stands out with only three CBL genes found in *Selaginella moellendorffii*, while the other four subgroups (1 to 4) contain 30, 19, 43, and 25 genes, respectively. NtCBL genes were identified in Clades 1, 2, 3, and 4, with Clade 3 being particularly abundant, housing up to five NtCBL genes (Fig. 1).

**Figure 2:**
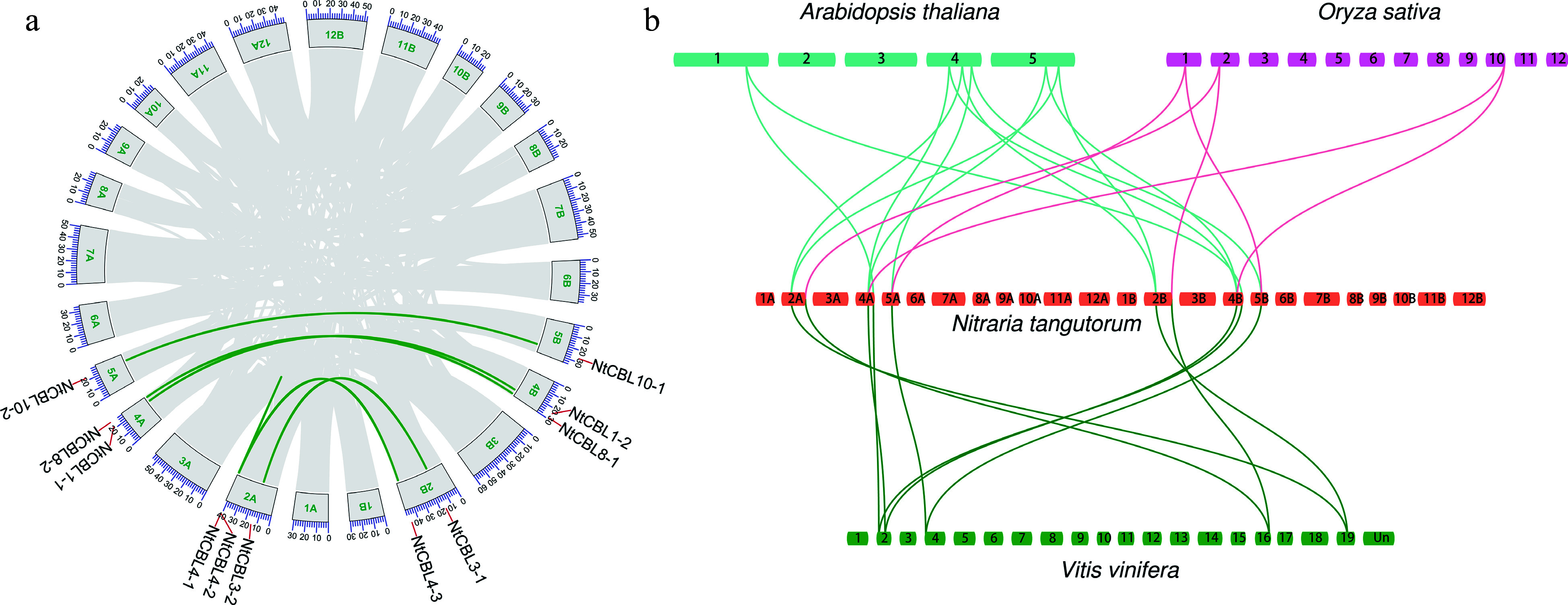
Genome-wide synteny analysis of CBL gene family among *N. tangutorum* and three other species. (a) inter-chromosomal relationships of NtCBLs (the links on the green curve indicate synteny relationships between genes). (b) Synteny analyses between the CBLs of *N. tangutorum*, *Arabidopsis*, *Vitis vinifera*, and *Oryza sativa*, the links between species indicate homologous relationships between genes.

To investigate the structural relationships among the 11 NtCBLs, we performed a genomic collinearity analysis using JCVI. Seven collinear CBL pairs were identified in the *N. tangutorum* genome: *NtCBL1-1* and *NtCBL1-2*, *NtCBL3-1* and *NtCBL3-2*, *NtCBL8-1* and *NtCBL8-2*, *NtCBL10-1* and *NtCBL10-2*, *NtCBL4-1* and *NtCBL4-2*, *NtCBL4-1* and *NtCBL4-3*, and *NtCBL4-2* and *NtCBL4-3* (Fig. 2a). Their chromosomal distribution and sequence similarity suggest that most NtCBL gene pairs were generated through whole genome duplication/polyploidization, while some NtCBLs, such as *NtCBL4-1* and *NtCBL4-2*, appear to have undergone tandem duplication.

We conducted Ka/Ks calculations for the gene pairs, uncovering that, aside from *NtCBL4-1* and *NtCBL4-2*, which displayed a Ks value of zero, the Ka/Ks values for the remaining gene pairs were notably below 1 (Supplemental Table S4). This observation suggests that these gene pairs underwent evolutionary purifying selection, emphasizing a constrained evolutionary process. Collinearity between genes from different species often indicates functional similarities. In light of this, we performed a collinear analysis of CBL genes across *Arabidopsis*, rice, grape, and *N. tangutorum*. The outcome indicated the existence of 12, 6, and 10 collinear relationships between NtCBLs and *Arabidopsis*, rice, and grape, respectively (Fig. 2b). This suggests that NtCBLs may share a close evolutionary relationship with *Arabidopsis* and grape, while showing a relatively distant relationship with rice.

### Gene structure and conserved motifs analysis of NtCBLs

**Figure 3:**
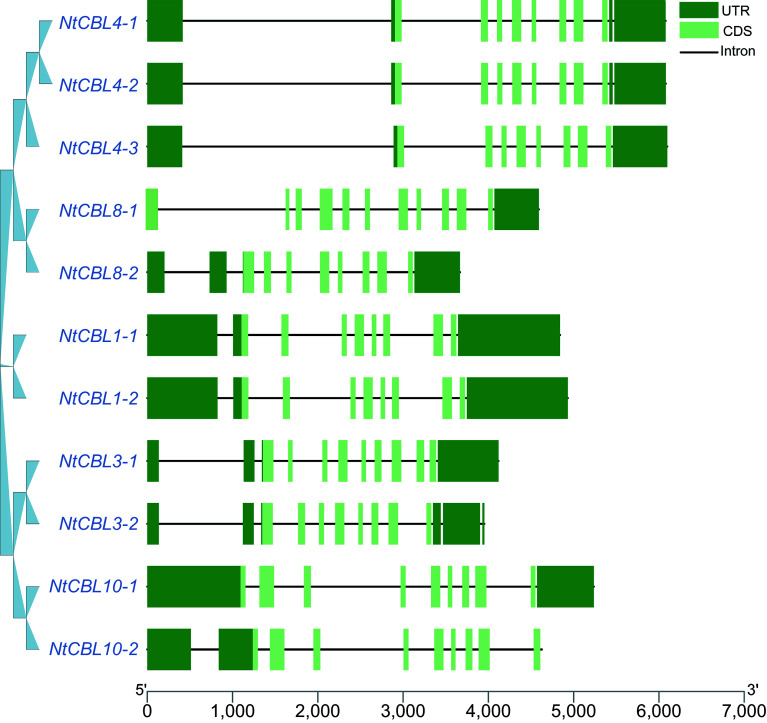
Gene structure of the CBL gene family in *N. tangutorum*. The dark green boxes represent the UTR (Untranslated Region), the light green boxes represent the CDS (gene coding region), and the black lines represent the intron region.

To obtain a more comprehensive understanding of the gene structure of CBL genes in *N. tangutorum*, we conducted an analysis of their gene structures. We observed that all CBL genes exhibited intron structures, with gene lengths spanning from 3,667 to 6,098 base pairs and coding sequence (CDS) lengths ranging from 8 to 11 exons. Notably, a lack of a 5' UTR region was observed in *NtCBL8-1*, while *NtCBL10-2* lacked a 3' UTR region. Conversely, typical 5' UTR and 3' UTR structures were present in the remaining genes (Fig. 3).

**Figure 4:**
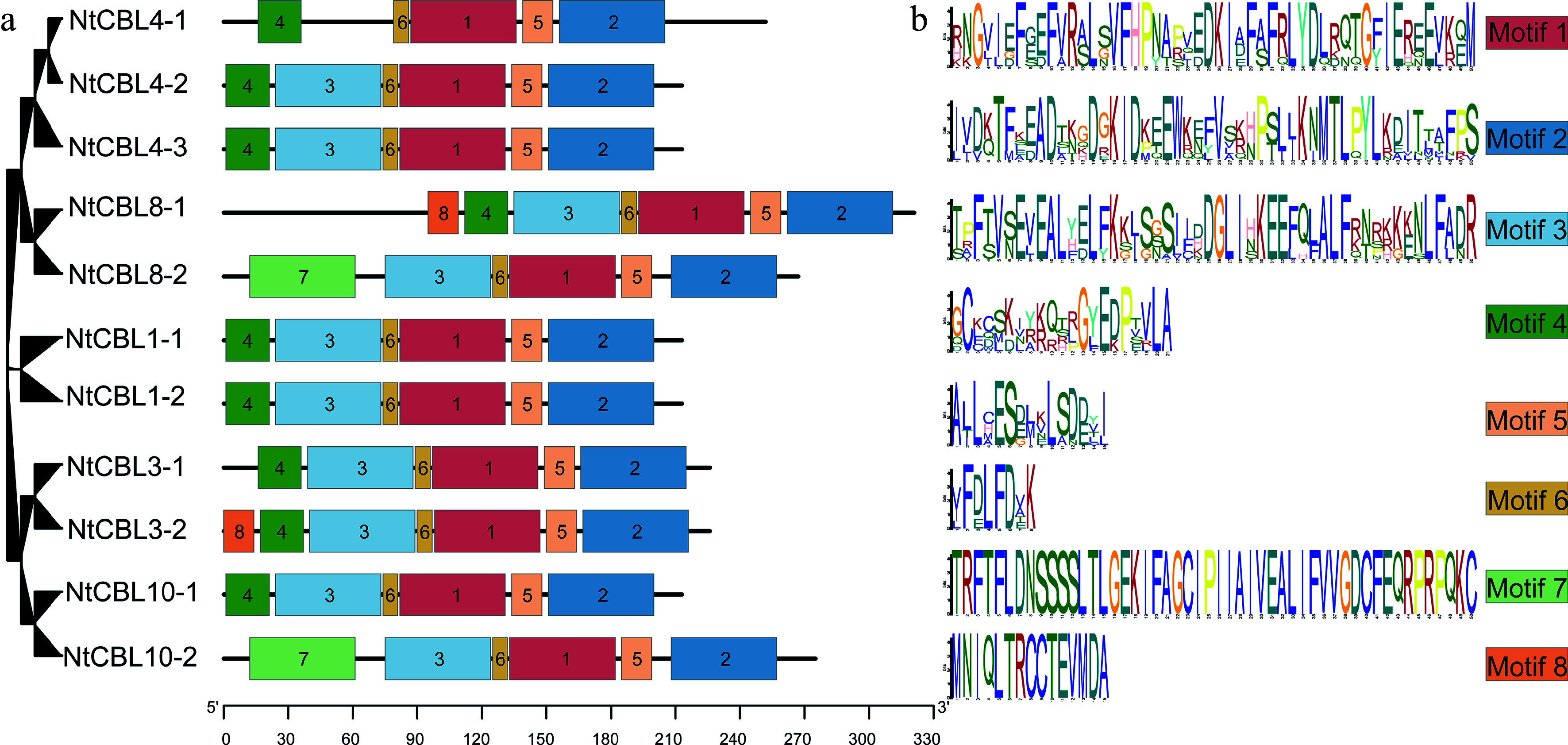
Conservative motifs distribution in *N. tangutorum*. (a) Distribution of different motifs on NtCBL genes. (b) Specific sequence information of different motifs, with larger letters indicating higher conservation.

Simultaneously, the pattern distribution within NtCBL proteins was investigated. As shown in Fig. 4, eight major motifs were identified among NtCBLs. Notably, motifs 1, 2, and 6 exhibited the highest degree of conservation of all CBLs. However, it is worth noting that motif 3 was absent in NtCBL4-1. In addition, pattern alterations were observed in four gene pairs that underwent fragment duplication, such as *NtCBL8-1* and *NtCBL8-2*, as well as *NtCBL10-1* and *NtCBL10-2*. Collectively, these findings suggest that functional differentiation may have occurred in NtCBLs during evolution.

### Analysis of *Cis*-regulatory elements in NtCBLs

Promoter *cis*-acting elements constitute a critical region for transcription initiation, as highlighted by Hernandez-Garcia & Finer^[25]^. Analysis of these elements holds significant value in unraveling the potential functions of genes. To investigate plausible biological roles of the *N. tangutorum* CBL gene family. We examined the sequence located 3 kb upstream of the NtCBL gene initiation codon for *cis*-acting element analysis.

This analysis unveiled the existence of a multitude of cis-regulatory elements intricately associated with hormone and stress responses. Specifically, cis-acting elements associated with abscisic acid responsiveness, auxin-responsive elements, methyl jasmonate (MeJA) responsiveness, gibberellin responsiveness, and salicylic acid responsiveness were identified within hormone signaling pathways. In addition, a range of abiotic stress-related elements were identified, including anaerobic induction, defense and stress responsiveness, drought inducibility, and low-temperature responsiveness (Supplemental Fig. S1). Collectively, these findings strongly suggest that CBL genes may indeed participate in biological functions related to these hormone and stress response pathways.

### NtCBL gene expression in various tissues and under drought and salt stress

Relevant studies have shown that CBLs play a role in drought and salt stress processes in plants^[26]^. Meanwhile, previous analysis revealed cis-acting elements associated with drought or salt stress response in the NtCBL promoter region. All these findings point to the potential involvement of NtCBLs in drought or salt stress responses in *N. tangutorum*.

Therefore, we have devised a set of experiments using qRT-PCR to investigate the expression patterns of NtCBL genes in different tissues and under abiotic salt stress conditions. First, we conducted an analysis of sequence similarity among NtCBLs (Supplemental Table S5). Due to the tetraploid nature of *N. tangutorum*, the genes within the NtCBL gene family exhibit extremely high similarity (nearly exceeding 95%). This high similarity made it nearly impossible to design specific primers. Therefore, *NtCBL1-1* and *NtCBL1-2* were combined as *NtCBL1* for qRT-PCR, and the same approach was applied to the other genes.

**Figure 5:**
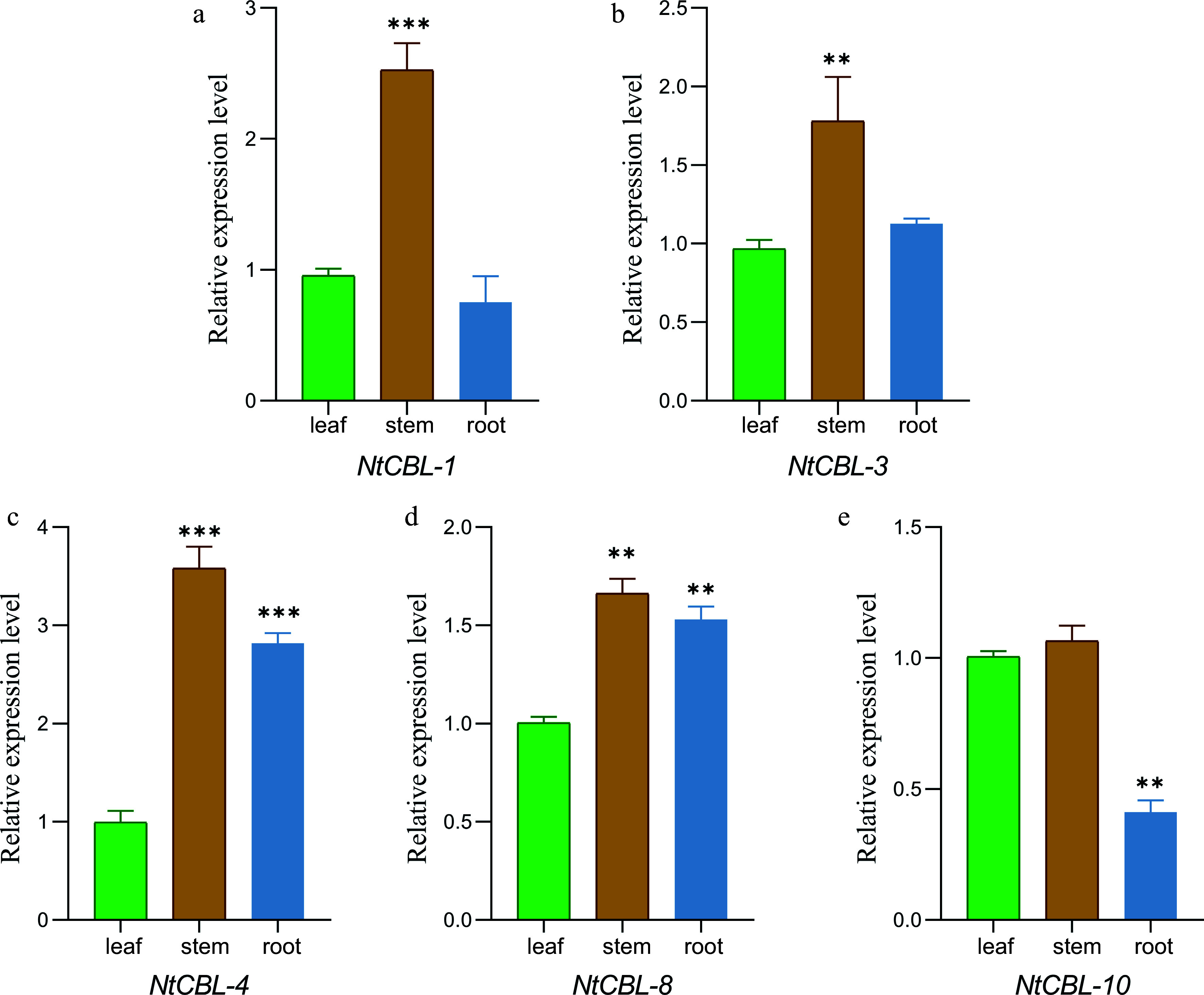
The gene expression characteristics of *N. tangutorum* CBL genes in root, stem, and leaf tissues were analyzed by fluorescence quantitative PCR. **p < 0.01, ***p < 0.001 (ANOVA followed by Tukey’s HSD).

The expression patterns of the NtCBL gene family in the roots, stems, and leaves of *N. tangutorum* were initially examined through a comparison of expression results across different tissues. It was observed that the relative expression of all NtCBLs was highest in the stem. NtCBLs could be roughly divided into two groups based on their expression patterns. In the first group, which included *NtCBL-1* and *NtCBL-10*, gene expression was ranked stem > leaf > root. In the second group, which included *NtCBL-3*, *NtCBL-4*, and *NtCBL-8*, gene expression was ranked stem > root > leaf. These different expression patterns of NtCBLs suggest that they may have distinct, tissue-specific functions (Fig. 5).

**Figure 6:**
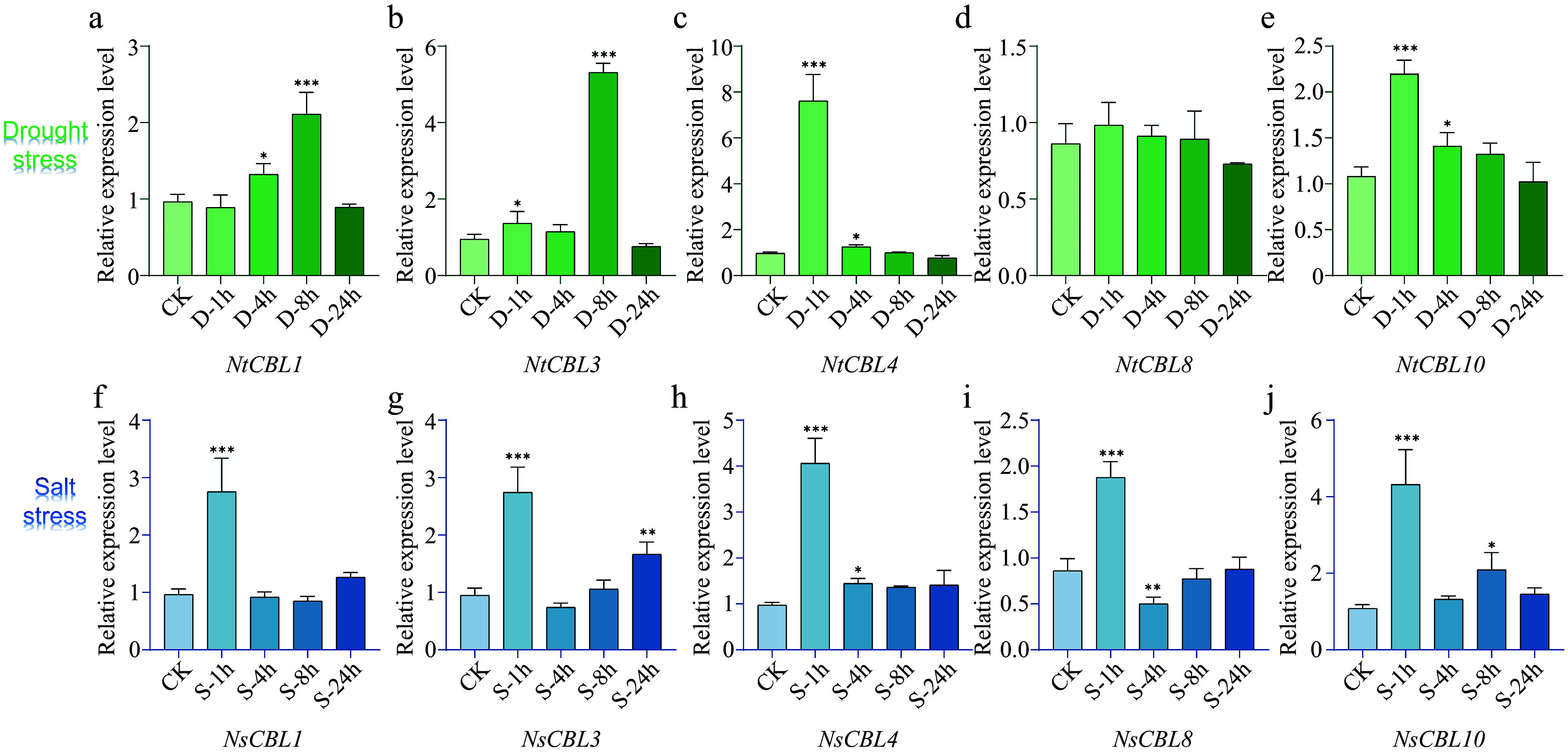
Expression patterns of NtCBL genes during drought (upper panels) and salt stress (lower panels) in *N. tangutorum*. 'ck' stands for untreated control, while '1h', '4h', '8h', and '24h' respectively represent different time points of salt or stress treatment, which are 1, 4, 8, and 24 h. **p < 0.01, ***p < 0.001 (ANOVA followed by Tukey’s HSD).

Subsequently, we examined the expression patterns of NtCBL genes under salt and drought stress. The majority of NtCBLs demonstrated distinct responses to these stresses, although the timing and magnitude of the response differed among individual genes (Fig. 6). Under drought stress, the expression of *NtCBL1* and *NtCBL3* peaked at 8 h, whereas *NtCBL4* and *NtCBL10* reached their highest expression levels at 1 h, and *NtCBL8* showed no significant change throughout the experiment. All NtCBL genes were significantly upregulated under salt stress, with their expression peaking at 1 h of salt stress and subsequently declining. These findings unequivocally illustrate that the expression of NtCBL genes is highly influenced by drought and salt stress, implying that NtCBL proteins likely contribute to *N. tangutorum*'s response to these challenging environmental conditions.

### Exogenous expression of *NtCBL1-1* enhances germination rate during salt and drought stress

With reference to research in other plant species^[27−29]^ and response to drought and salt stress in *N.tangutorum,* we chose *NtCBL1* as the representative gene for NtCBL and examined its potential role in enhancing stress resistance through overexpression in *Arabidopsis*. We designed specific primers only for *NtCBL1* (Supplemental Table S5), and confirmed that the cloned gene was *NtCBL1-1* by first-generation sequencing comparison, then constructed the 35S:*NtCBL1-1* overexpression vector and transformed it into wild-type *Arabidopsis* *via* Agrobacterium-mediated transformation to obtain positive transgenic plants. We obtained multiple transgenic positive lines (Supplemental Fig. S2) and randomly selected eight of these lines for analysis of their relative expression levels. We observed variations in the expression levels among these lines (Supplemental Fig. S3). Based on their expression levels, we specifically chose lines 1, 4, and 6 and renamed them as lines 1, 2, and 3, respectively, for subsequent functional validation of *NtCBL1-1*.

**Figure 7:**
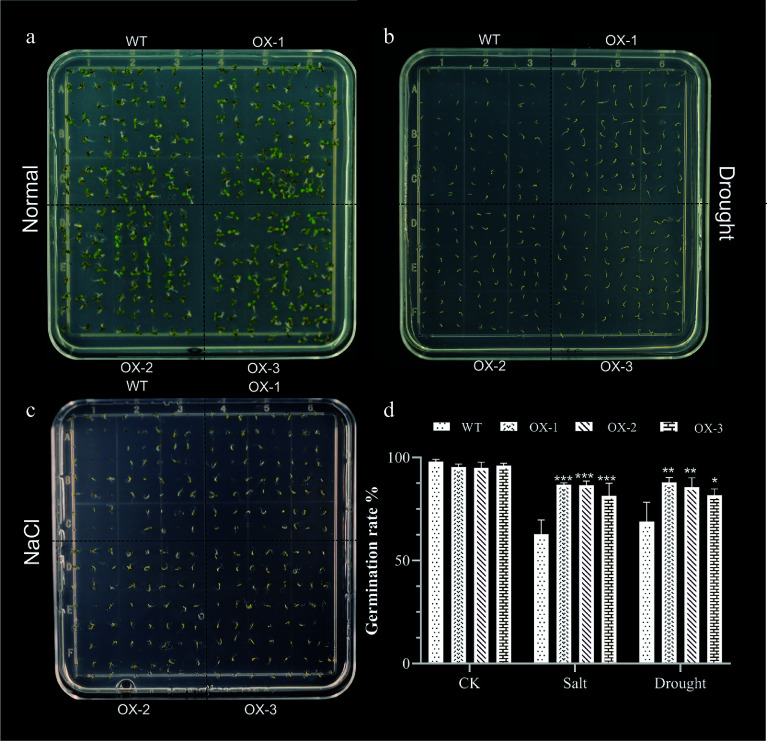
Heterologous expression of *NtCBL1-1* increases *Arabidopsis* germination rates under salt and drought stress. (a)−(c) Phenotypic charts of germination rates under normal growth conditions, 150 mM NaCl treatment, and 300 mM mannitol treatment, with about 70 seeds per dish and three replicates for each experiment. (d) Germination rate statistics of *Arabidopsis* overexpressing *NtCBL1-1* under normal growth conditions, 150 mM NaCl treatment, and 300 mM mannitol treatment. *p < 0.05, **p < 0.01, ***p < 0.001 (ANOVA followed by Tukey’s HSD).

Previous studies have shown that stress can significantly affect seed germination rates^[30]^. To determine whether heterologous overexpression of *NtCBL1-1* affected *Arabidopsis* germination, we sowed seeds of wild-type *Arabidopsis* and three independent transgenic lines on ½ MS medium containing 0 or 150 mM NaCl or 300 mM mannitol, and we observed their germination rate after 7 d. Under normal growing conditions, wild-type and transgenic lines germinated rapidly, and their germination rates were similar (Fig. 7). Under 150 mM salt stress, the germination rate of wild-type *Arabidopsis* was reduced, and the three overexpression lines showed higher germination than wild *Arabidopsis*. Under drought stress, both wild-type and transgenic lines showed reduced germination compared to control conditions, but the germination rate of transgenic lines was significantly higher than that of wild-type lines. Thus, overexpression of *NtCBL1-1* in *Arabidopsis* ameliorated—at least in part—the inhibition of germination caused by salt and drought stress.

### Exogenous expression of *NtCBL1-1* enhances drought and salt stress tolerance in *Arabidopsis*

**Figure 8:**
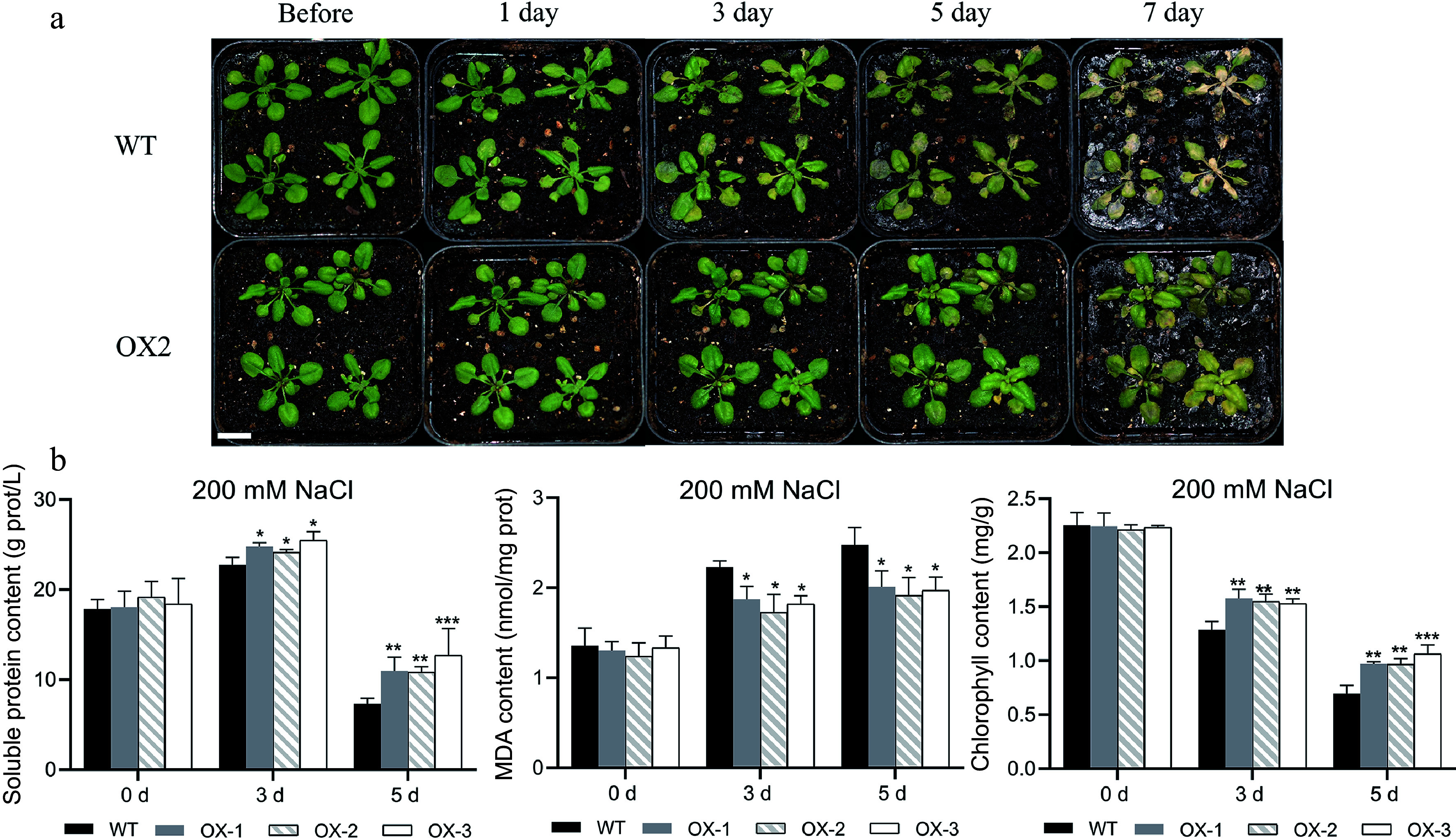
Heterologous overexpression of *NtCBL1-1* increased salt tolerance in *Arabidopsis*. (a) Wild-type *Arabidopsis* phenotypes and overexpression lines exposed to 200 mM NaCl for 0–7 d. (b) Chlorophyll content, soluble protein content, and MDA content after 0–5 d of exposure to 200 mM NaCl. Statistical significance denoted as *p < 0.05, **p < 0.01, ***p < 0.001 (ANOVA followed by Tukey’s HSD).

To assess whether the heterologous expression of *NtCBL1-1* impacts the salt and drought tolerance of *Arabidopsis*, we subjected soil-grown seedlings to irrigation with 200 mM NaCl or 300 mM mannitol for a duration of 7 d. On the first day of salt stress, leaves of wild-type *Arabidopsis* began to show slight wilting, but transgenic plants overexpressing *NtCBL1-1* showed no visible changes (Fig. 8a, Supplemental Fig. S4). On day 3, leaf yellowing became visible on wild-type *Arabidopsi*s, and the transgenic plants began to wilt. On day 5, leaves of wild-type *Arabidopsis* were severely wilted and showed large areas of yellowing, whereas leaves of the transgenic plants had begun to turn yellow. On day 7, wild-type plants were completely withered, but only some leaves of the transgenic plants were withered and yellow.

Since the wild-type plants had nearly died by the 7^th^ day of the salt stress treatment, we conducted physiological measurements exclusively on plants harvested on days 0, 3, and 5. Before salt stress treatment, there were no significant differences in chlorophyll content between wild-type and transgenic lines. Chlorophyll content decreased as the duration of salt stress increased in both wild-type and transgenic *Arabidopsis*, but chlorophyll content was significantly higher in transgenic lines than in the wild type. This difference was most striking on day 5 (Fig. 8b). Soluble protein content showed a trend similar to that of chlorophyll content; it was lower in the wild type than in the transgenic lines under salt stress. Although all genotypes showed accumulation of MDA during the stress treatment, MDA content was significantly lower in the transgenic lines.

**Figure 9:**
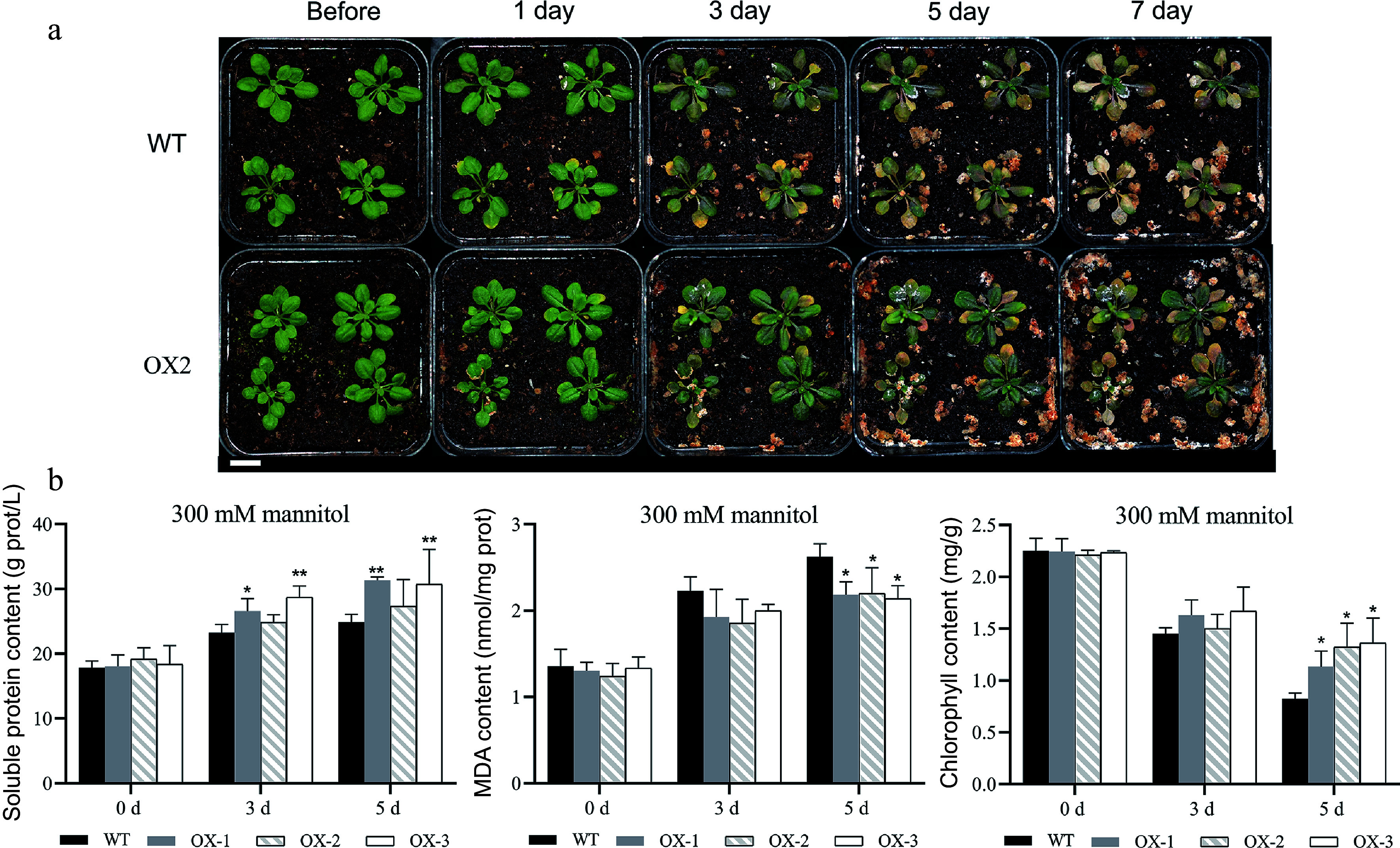
Heterologous overexpression of *NtCBL1-1* in *Arabidopsis* increased drought stress tolerance. (a) Phenotypic comparison between wild-type *Arabidopsis* and overexpression lines under 300 mM mannitol exposure for 0–7 d. (b) Chlorophyll content, soluble protein content, and MDA content of wild-type *Arabidopsis* and overexpression lines exposed to 300 mM mannitol for 0–5 d. Statistical significance denoted as *p < 0.05, **p < 0.01 (ANOVA followed by Tukey’s HSD).

The transgenic lines also showed less severe stress symptoms than the wild type in response to simulated drought (Fig. 9a, Supplemental Fig. S5). In contrast to the salt stress results, there were no significant differences in MDA or soluble protein content on day 3 of drought stress. However, on day 5, the MDA content was significantly higher in the wild type than in the transgenic lines, and the soluble protein content was significantly lower (Fig. 9b). Thus, overexpression of *NtCBL1-1* also improved drought stress tolerance of transgenic *Arabidopsis*.

## Discussion

Deterioration of the soil environment significantly limits plant growth and development, and soil salinization is becoming an increasingly serious threat to forestry and agricultural production. As of 2021, about one billion hectares of land worldwide have been affected by soil salinization, accounting for about 7% of the Earth's total land area^[31]^. Excessive salinization impairs the normal growth of most plants, but halophytes can typically complete their entire life cycle under such conditions. Studying the adaptive mechanisms that enable halophytes to thrive under saline-alkali conditions is therefore important for the utilization and restoration of saline-alkali land.

As important Ca^2+^ sensors, CBLs have key roles in plant perception and response to a variety of abiotic stresses, including drought^[32]^, salinity^[33]^, and cold^[34]^, as well as the stress hormone ABA^[35]^. CBLs have been studied in a number plants, and 10, 9, and 7 CBL genes have been identified and characterized in *Arabidopsis*^[4]^, *Saccharum spontaneum*^[36]^, and *Triticum aestivum*^[37]^, respectively. However, the number of CBL genes and their functions in the halophyte *N. tangutorum* have not previously been reported. In this study, we identified 11 CBL genes in the *N. tangutorum* genome and classified them into four clades on the basis of their phylogenetic relationships. Each clade showed general conservation of gene structure and protein domain composition, although there were some differences (Fig. 4) that may reflect functional differentiation of the NtCBLs.

Plant polyploidization refers to the increase in the number of chromosomes within plant cells, often manifested as the duplication of entire sets of chromosomes^[38]^. Polyploidization leads to the presence of multiple identical or highly similar gene copies within the genome. These duplicated copies of genes undergo mutations during evolution and accumulate new features, driving the expansion of gene families. For instance, in the polyploid crop *Brassica napus*, the number of FBA gene family members is higher than in diploid plants such as *Arabidopsis* and rice^[39]^. Additionally, the MADS-box gene count in tetraploid *Gossypium hirsutum* was significantly higher than diploid *Gossypium hirsutum*^[40]^.

Plant polyploidization also leads to gene redundancy and enhanced stability^[41]^. In *N. tangutorum*, all CBL genes have homologs in their corresponding subgenomes. That is, except for the *NtCBL4* gene, all NtCBL genes exhibit two highly similar gene duplications on corresponding chromosomes in their respective subgenomes. For example, *NtCBL1-1* and *NtCBL1-2* occur on CHR4A and CHR4B, while *NtCBL10-1* and *NtCBL10-2* are located on CHR5A and CHR5B, respectively. This duplication probably results from the tetraploidization process in *N. tangutorum*. In general, polyploidization offers significant benefits to plants, especially in adapting to environmental changes. This may be one reason *N. tangutorum* adapts to extreme saline-alkaline and drought conditions. Through polyploidization, plants may produce more copies of genes, and these copies could mutate, leading to new traits or functions. This diversity helps plants better adapt to various pressures or environmental conditions and helps them maintain a competitive advantage during their evolutionary process.

Apart from chromosomal duplication, gene self-replication and differentiation also play significant roles in the expansion and evolution of gene families. Systematic phylogenetic analysis in this study reveals the presence of multiple copies of the CBL4 gene in several non-polyploid plants. For instance, there are three copies in *Populus euphratica*, five copies in *E*. *grandis*, two copies in *C*. *sinensis*, and three copies in *M*. *domestica* (Fig. 1). Multiple copies of CBL4 have also been observed in other plants, such as two copies in canola^[5]^, and three copies in *M. sativa *^[42]^. Generally, gene redundancy is critical to maintaining essential biological functions in certain plants and may play a pivotal role in their adaptability and survival. Expansion of the CBL4 subfamily may be closely related to this phenomenon. In *N*. *tangutorum*, despite being a polyploid plant, the CBL4 gene had undergone replication prior to polyploidization, resulting in the formation of *NtCBL4-1* and *NtCBL4-2*, suggesting a potentially significant role for CBL4 in the life processes of *N. tangutorum*.

The *cis*-acting elements in the promoter region can regulate gene expression, thereby revealing internal information about gene function^[43]^. Analysis of the NtCBL promoter reveals various cis-acting elements associated with hormone regulation and defense responses. For instance, within the NtCBL gene promoter region, there are abscisic acid (ABA) responsive elements known as ABREs (Fig. 5). In the promoter regions of CBL genes in other species such as pepper, quinoa, and *Vitis vinifera*, we have found ABRE elements, indicating the relative conservation of ABRE elements in the CBL gene promoter regions. When plants face stress like drought, their water levels decrease, leading to the production of ABA. The ABA signaling pathway is activated, triggering a series of physiological and biochemical responses to help plants adapt to drought stress^[44]^. The varying responses of CBL genes to drought in pepper^[45]^, quinoa^[46]^, *Vitis vinifera *^[^^6^^]^, and *N*. *tangutorum* might be associated with ABRE elements.

Genes typically display patterns of tissue-specific expression. In our study, we observed predominant expression of CBL genes in stem and root, similar to the expression pattern of CBL in rice. However, in rice, CBL4 exhibits major expression in leaves^[47]^, while in *N*. *tangutorum*, *NtCBL4* exhibits the least expression in leaves. In cotton research, the expression of *GhCBL4-5* was found to be higher in stem and root compared to leaves^[48]^, which is somewhat similar to the expression pattern of NtCBL4 in this study. Despite differences in the expression patterns of CBL genes across species and tissues, they exhibit notable responses to stressors such as drought, salinity, and other adverse conditions. This high degree of conservancy in their response to environmental stressors might indicate the pivotal role CBL genes play in physiological regulation and stress responses in plants. Wide adaptability suggests the universality and significance of CBL genes and their regulated signaling pathways across different species.

Currently, multiple genes within the CBL family have been cloned and their function under non-biological stressors has been validated^[49−51]^. Among these genes, *CBL1* is one of the most extensively studied representatives within the CBL gene family. *CBL1* has been studied in many plant species, including *Arabidopsis*^[33]^ and *Brassica napus*^[27]^, but its function in *N. tangutorum* remains to be fully characterized. Here, we selected *NtCBL1-1* as a representative of the NtCBL gene family and performed a preliminary functional characterization by overexpression in *Arabidopsis*. Although the sequences of *NtCBL1-1* and *NtCBL1-2* were highly similar, we were able to design specific primers for cloning *NtCBL1-1*, and we confirmed the identity of the cloned gene by Sanger sequencing. We then used this gene to construct an overexpression vector for *Arabidopsis* transformation.

Previous reports on CBL1 in other species, as well as our own qRT-PCR results, led us to speculate that *NtCBL1-1* might participate in responses to salt and drought stress. Although both stresses impaired seed germination, seeds of the *NtCBL1-1* overexpression lines had a higher germination rate than those of the wild type under stressed conditions (Fig. 8). Likewise, the transgenic plants showed less severe symptoms in response to salt stress and simulated drought. Leaves of the wild type showed severe wilting and yellowing under both stresses, whereas those of transgenic plants were only slightly yellow, and this difference became more obvious with increasing stress duration.

Studies have shown that plant stress can lead to chlorophyll degradation^[52]^, and plants with greater stress resistance tend to have higher soluble protein content^[53]^. MDA is a lipid peroxidation product whose content can serve as a measure of oxidative damage^[54]^.

Overexpression of stress-resistant genes may affect plant physiological characteristics, such as soluble protein content, malondialdehyde (MDA) levels, and chlorophyll content. These physiological traits are often associated with a plant's ability to withstand stress and its mechanisms for coping with environmental pressures. Over-expression of stress-resistant genes typically triggers the activation of signaling pathways, leading to a series of biochemical responses in plants. This results in the production of more stress-resistant proteins and metabolites, including soluble proteins and antioxidant enzymes. This process helps alleviate oxidative stress, enhances the plant's resistance to free radicals and oxidative damage, thereby reducing the accumulation of oxidative byproducts such as soluble proteins and MDA. Chlorophyll, a key molecule in photosynthesis, plays an important role in protecting plants from environmental stressors. Overexpression of stress-resistant genes can reduce the damage to chlorophyll caused by stress factors, helping maintain photosynthetic efficiency and normal plant growth.

For example, researchers have found that overexpression of the stress-resistant gene *HvPIP2;5* in transgenic *Arabidopsis* leads to retention of more chlorophyll and a reduction in MDA accumulation^[55]^. In another study, overexpression of the superoxide dismutase gene reduced the accumulation of soluble proteins in alfalfa leaf tissues^[56]^.

These findings suggest that by overexpressing stress-resistant genes, plants can better cope with environmental stress, reduce oxidative damage, and reduce the accumulation of oxidative byproducts, thus maintaining their normal growth and survival.

In this study, transgenic *Arabidopsis* overexpressing *NtCBL1-1* contained more chlorophyll and soluble protein and less MDA than the wild type under salt stress (Fig. 8), indicating that they had higher salt tolerance. Responses to simulated drought stress were similar: *Arabidopsis* overexpressing *NtCBL1-1* showed greater drought tolerance than the wild type (Fig. 9). Thus, overexpression of *CBL1-1* from *N. tangutorum* improved the abiotic stress tolerance of transgenic *Arabidopsis*. The calcium receptor encoded by *NtCBL1-1* may therefore form part of the signal transduction pathways that underlie the high salt tolerance of this dryland halophyte. Overexpression of the *NtCBL1-1* may have activated multiple physiological pathways in *Arabidopsis*, aiding the plant in better coping with environmental stress, alleviating oxidative stress, protecting chlorophyll, and maintaining water balance. These physiological changes enhance the stress resistance of the transgenic *Arabidopsis*.

This study revealed that the overexpression of the *NtCBL1-1* in *Arabidopsis* led to enhanced phenotypes, germination rates, and chlorophyll content under drought stress compared to wild-type *Arabidopsis*. However, this is just the tip of the iceberg, as we still need a more comprehensive understanding of the underlying regulatory networks. In parallel, we are actively engaged in the development of a transgenic system specifically tailored for *N. tangutorum*. We eagerly anticipate conducting in-depth mechanistic inquiries through self-overexpression analysis. Moreover, the translocation of CBL into *N. tangutorum* holds the promise of yielding more robust salt-tolerant halophytes. This prospective outcome could offer valuable insights for future biotechnology-driven advancements in forestry breeding and ecological restoration.

## Conclusions

In this study, we identified CBL family members in *N. tangutorum* and characterized their basic physicochemical properties, phylogeny, and responses to stress. The results indicate that there are a total of 11 NtCBL genes distributed across six chromosomes in *N. tangutorum*. Expression analysis reveals that these genes are highly responsive to both salt and drought stress. Functional studies of *NtCBL1-1* demonstrate that transgenic Arabidopsis plants overexpressing *NtCBL1-1* exhibit enhanced tolerance to both drought and salt stress. Under drought and salt stress conditions, compared to wild-type *Arabidopsis*, transgenic plants show increased germination rates, slower chlorophyll degradation, higher accumulation of soluble proteins, and reduced levels of the oxidative stress marker malondialdehyde. These findings underscore the significant role of *NtCBL1-1* in responding to drought and salt stress and provide insight into the evolution and biological functions of the CBL gene family in *N. tangutorum*, providing a theoretical foundation for further study of abiotic stress resistance mechanisms in this desert halophyte.

## SUPPLEMENTARY DATA

Supplementary data to this article can be found online.

## Data Availability

All data generated or analyzed during this study are included in this article and its supplementary table files. The Gene sequence, CDS sequence, and GFF annotation information of all NtCBL gene families are included in the supplementary files.
