# Supplementary material for: Genome-wide discovery of CBL genes in Nitraria tangutorum Bobr. and functional analysis of NtCBL1-1 under drought and salt stress
Source: For Res (Fayettev). 2023 Dec 22;3:28. doi: 10.48130/FR-2023-0028 (PMC11524306; doi:10.48130/FR-2023-0028)
Supplement: Supplementary file 1 — Supplementary data to this article can be found online. [file FR-2023-0028-S1.zip › 10.48130_FR-2023-0028-Suppl-FigureS1.pdf]

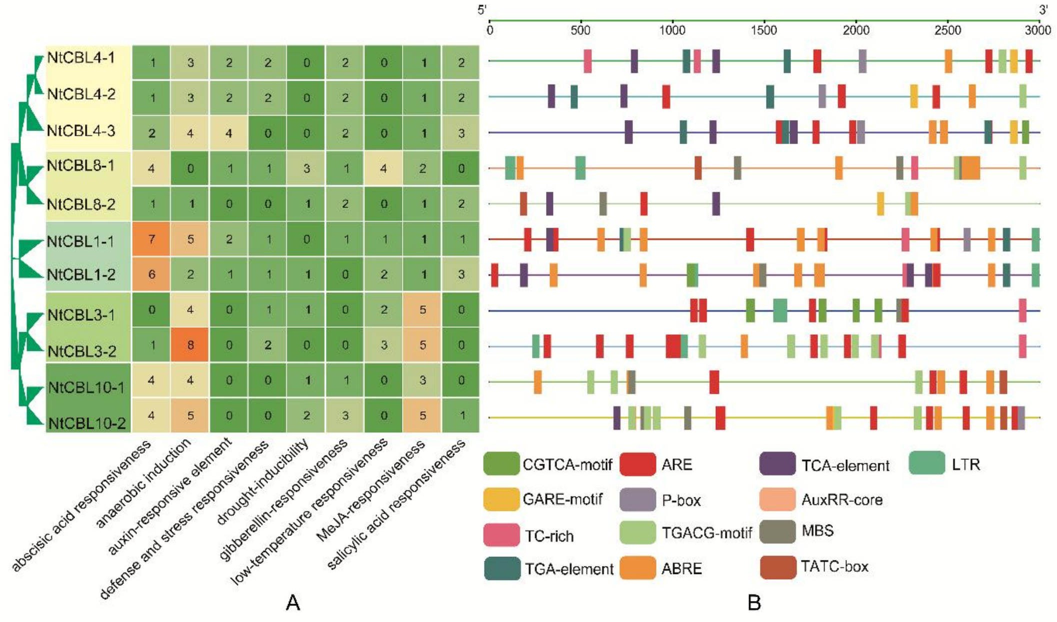

Figure S1 *Cis*-regulatory elements analysis of NtCBLs. (A) A heat map showing the number of *cis*-acting elements related to stress response in NtCBLs. (B) The distribution of cis-acting elements in the promoter region (ATG upstream) of NtCBLs, where different boxes represent different cis-acting elements.
