# Supplementary material for: Genome-wide discovery of CBL genes in Nitraria tangutorum Bobr. and functional analysis of NtCBL1-1 under drought and salt stress
Source: For Res (Fayettev). 2023 Dec 22;3:28. doi: 10.48130/FR-2023-0028 (PMC11524306; doi:10.48130/FR-2023-0028)
Supplement: Supplementary file 1 — Supplementary data to this article can be found online. [file FR-2023-0028-S1.zip › 10.48130_FR-2023-0028-Suppl-FileS1.docx]

**CBL_genome.fasta**

>NITAA04G1075 CHR4A 21576777 21581709

AAAAAATCCCTATTTTAGTGAAATCTATATGGTCTGAATTAATAATTAAAATCTCTTCAA

TAATTAAAAATGGTATTTTTTTGTAGTTTTGTATAATTTGTTGCCTTTCTTAATGATCAA

TTGCCAAAAATTAATAAAAGACAACTACATGAAGTCCTTTGAAAGTGCGAAACTAATTGA

AAATTTAGACAAACACAAATAGACCATAATTGGTCTTTTTCACAGGAAAGAGAAAGCACG

TTCTCCTCTACCTTAGAGGAGACTGATCTCCTCACCATAGACTAGACCCAATCTCCTCAA

CGCCTATCAAGAAGGAGCATGTTCGCACCGGCAGAGGACACCTGACTTGTGCTCTCTTCT

GAATAGCGCCGAGTTCTTCCTCGAATGGGAGAAACAGAATACATCCACTAGAGCCGACAA

GTTTCCTCTACCAACAGAAAGCGGTAAAGTTCACCGGCAAAGGAGACCAAATCCCTCCAC

CAATGGAGGTCATTGTATCCGGAAGTCAACGGACATGATGACCAGGCGAACTTTTTTAAA

CTAAGTGTGACTTCTTTAACATTTCACAGACTTCCTGTGGCCAGTTAACTTTTCCCCTCT

CCCAAAATAAGGCATAAATTTTCTCTCAAATGATAAAGAAGAACTTTTTAAAAATGAAAA

AGGGGAAGTATCACGGAAACTTTCTATTACTATTTGGGTGCCGCTAAAATGAACTCTATA

AAACCAAGAAGAAATCAATTACAGAACTTGCATTTTAAGGCTTCAACTTGCGCCCGACGG

CAAGATTAAAGGATCCAAACACAAATTCAACTTTCCTATGAATTAACTCTCAATGATAGA

TGCACCCCTAAGGTTTGGTTTAGGCTTAGAGCGGTGCCAAATTCTGACCAACCACGACCA

ACGACATCCGATCACATAACGGAGAGATCTATATTTCTATACACTGCGCTTTCCCAAGAT

TGTTTTTGCAGAAAGACCACCGCAAAATCACTCGAAATATCAACTCACTTCTTAATTTAC

ACTAGTACATTTGACAAACTCTACAGTTGACCGACTTTCCAACCCAGTAAACTTCAGAAG

CTTCAATCAACATACAGACTCCATCTTCAATAGTTAAAAAGAAATACCAAAAAAAGAAAT

GATATCATACTAATTTTCAAATCCCTAAATCTAATTCCTGTCTAAATTATGTGGCAAGTT

CATCCACTTCCGAATTAAAAACAAAACTGGGAAAAGTCGTCGTTATGTCCCTGCAACCAG

AAATATACAAAATGAGTATATCAACCAGAAGTTTTTAACACTAGGAAGTGAGTCTTTACG

GCGCAGTTTGTCATCACATACCTCAAATAAGGAAGAGTCATGATCTTCAACAAAGATGGG

TTTTTAGAGACAAAGTTTTGCCATTCTGTTTTATCTATTTTTCCATCCTGGTTTGCATCG

GCTTCCAAGAAAGTCTGCCCAAGAAAGTAAGGGCATTGTGAACTTTTAGGCAATCTAGGA

AAATGCAAACTAATGAAGAATACACACAGACTGTAAAAAAATAGACCGACTGGAAATGAA

CCATTGCCTTGCACTTATCATCCTCATTTACAGCACAAATTAATCATCAAATCTATCAGA

TTCGTATAACTGAATGAATTATTACCAATGCTTCATGAGCAAAAAAGCAACAACAACATC

AAACTTCTATTTAAAACTCCCAGGATATCCAACAGAAGCCAACATGTCATTTCTGTAATT

TTGTAAAGGATACACATCCGCAAACTAAAACCAAGTTTTCTTGTAGATTACAAATGTTGA

CCGAAAAGCTATGATTTGTTATTAGAATGAAAGATTCTAAATGTCTGGGCTGCCGAAAAA

TTCTAAGCATCTACGGTGAAGAATAGCTTTACATATATATGTGCTGAGAATGTTGATGCA

AAATATGTTCAATTTGTTGGATTTTGAAGAAGGCGAAACTTACCTTATCGAGTATTACTT

CAATCGTTTCATCAGCCAGCTTCATTTCAGATTCACATAGAAGTGCAATCAGCATTTGCT

TGACCTGCCACAATCACACCAATGAAGTTGCCACTATGATAGTTTTGAATTGTGACTCAA

CATATTAAACAGTACACCAACATACCTCTTCACGCTCGATGTATCCTGTATTATCTTGAT

CATATAGCCTAAATGAGACTGGAGTAGACAAGCAAAGATTAGATAAGTATGAATATACAA

CATGAATTTTAAGCCATCTAATGTAAATAGAACGCTTTCAGCCAACTTACAGTCAATCTT

GTCCTCTTGAGAAGCATTTGGATGAAAAACATTTAGTGATCTAACAAAATCACTAAAATC

AATGAGCCCCTTTCGCTTCACATCAAAAAGTTCAAAGAGCTGTATAACAAGACTAGTTAG

TCTAAAGGTCCAATTAACGGCCACGTACTTATAGAATGATAGGAAACTCGAGAGAAGATT

ATTAAAGATACCCTGTTGGCAAATAGATTTTCCTTCTTTCTGTTTTTGAAGAGAGCCAAC

TGAAACTCTTCCTACAAATAAGACACAAATTTTATATCAGATTTAGGTGCATCCTGCAAA

TTATATTGAATTCTTCACAATTACAACCCCATCCAGCCATCTCTGAATGTAGGATCATTT

TCAGTTCAGAAAAATTGAAGCCCTATATATGACCAAATAGCAGGTGGAGTTTGAAATTCA

ATATTCAGAAACCTTCCTTAACTAAACAAAATAATAGGACTATCTTACAATTAATTTGCG

TTACCAGTTACATTCTTGAGGCATTCACTTTCTCCATCAATAGACATAAAACTCAACAAC

TAACAAAGTGCTTAATCTAAGGGGAGGAGAAGGAGCGAAAGAGAAAGAGAAAGAGAGGGG

GGGGGGGGGGGGGTGTTGGATGGATTAATATGCTCCCAGAAGAACACGTCTTTTAAATGA

TTTTCATTGTTTTACGGACTGATCTCAGATTAGCCCTGCCATCACATTCACTATGGATTT

TTTTCAAAGTAACATGAGTCCATGAACAGATTATAACTACAGTAAAATCCTAAAACAGTA

GATCATCTAAAGTTTTAATTCATGTGTCACTTTGAGCCGACAACAATCACCACCCTTTTT

TATCTATGCTCACAGTCCAGTTCATGTTTCAATATGAACTTAACCAACAACAACCTCCTT

TACCAAGCTAAAATAACTGCAACTGTGGCTAGACAGATACAATCATCGGTGGACAACATA

CCTTGCTTATTAACCCATCGTCAATGAGGGAACCGCTAATGCTCTTGAATAGCTCAAACA

AGGCTTCAACTTCACTAACACTAACTGCAGATCCAAACCAGAGAACCAAATGTGTTCAAA

TAACATGCAACAAGGGATACATAGACAGACATAATGATGCAATAACAGAATGAATGCTGC

TATGACTTCAGAAGCCTTTTACACTTTTTCCCCTGTAAATTGAAATCAAAGAACACATAT

ATTTACATATTTTTTTAAATCATAGATACAATCTACAAGGAAAAGTGAAGCGCCTAGACC

GCTTAAACAGAGTAATCAGATAAGAATTTCTACTGTGGTTATAATACAGCATAAGAAATG

ATGACTAATATTATTCAAAAAAAAAAAAGAAATGATGACACGTAAAATACTTGTAACAAA

ATTTGAAATTGGTAACAGGGGATAAGCAGTTCTCATCTCAATGAAACAGTGGCTGGACTA

CAACTTACACGCAGTTTGCGAAGCCAGAACAACTGGGTCCTCATACCCAGGGTGCTGCCT

CGCTACCTTTGACTGAAAACACCCCATTTATCTACCAGAAAAACACGACAACGAAGAAGC

CGAAAATATGCCTTGCTTAAACTCTTAGCTTCCTCAACAAGATTATAGTATCACTATCTT

CAAAACCCTCAGCATCTTTAACACCTAGCAATTTGCACAAAAAGAAAAGAGCCTAATTCA

AAGAGAATATTAATTCATTGACAGAGAATCTTTATGAAAACGACACCGGCGTGAACAAAA

GCTATTTAACAAATGCACATCAGAACCTACCAACAATGATAATCAAAACAGATGATATAG

CACGTGGATTTTCAAATTATCTAGAAACCTGTTGAATTGCTTCTACAGTACTTGTACAGG

GAAGAGATTCATTCAATTTTATCTTGTGCTTCGTTTTTCTAGTTTTCTACCTCTCAGAAT

TTTAAGAACGGGAAAAAAAAAGGAGAGAAGCAGCGAAAAATTGAACTAACTCAACAGCGG

ATTGAAGATCTAGAATTTCTTTTATAATTATTCACCTCCAATTTCTTGGGAAACAAACAA

AGCAGAAAGTAAACGCAAATGAAATTCCAAGATCTTTTATTGCTATTATTCGAAGTTGTA

AACCAAAAAGATCACGTAAACACCTTCCCCAGAAAACAACAAAAAGTTGGAAAAGAAAGA

AAAATAAAAGATTAACCAAAAACGTCGAGTTCTGCTCAGCACTTTGCTATTAATAACTCT

AAGCATGAAAAGACACCAATTCAGAAGAGAGAAAAAGAAAAAGTAAGAACACAAAATCAA

AAGCGTGAAAAGGAAGCGAGACGGAGAGAAAGAGAGAAAAAAGAACTTACCGACCAAAAA

ATTGGCCGGAAAATGCAAAGCCGGGGCGACGATAAACAACCACTTTTCCCAATATTGTTT

GGCCTCTTAGAAAGAAAAGAAAGACAGAAAAAACAGAGAAGGTTTAGGTCGAGATTTAGC

TGGGCTAGTGGAGAGATTTGTGGCCTGTGTCTCTCGCCGAGGGGAGAGAGAGAGAGAAAG

AAAAACTTCTATGATTGATTGGGTGATCCAACGGGTGAGATTATATCCGATGAGCGAACA

GATTTTCCCGTATGTACAGTAGTACCAGTGTGTATACATGGATTTGCACATCTGTACATT

TTTTTGTCTTAAA

>NITAB04G1197 CHR4B 24367647 24372487

AAAAAAAAACTATTTTAATGAAATCTATATGGTCTGAATTAATTAAAATCTCTTATATAA

TTATAAAAGGTATTTTTGTAGTTTTGTATAATTTGTTGACTTTCTTAATAATCAATTGAC

AAAAATTAATAAAAGACAACTACATGAAGTTTTTTGAAAGTGCGAAGCTAATTGAAAATT

TAGACAAACACAAAGAGACCATAATTGGTCTTTTTCACAGGAAGGAGAAAGCACATTCTC

TTCTACGTTAGAGGAGACTAATCTCCTCACCATAGACTAGACCCAATCTCCTCGACGTCT

GTCAAGAAGGGGCATGTCTGCACCGGCAGAGGATACGAGGTTTGTGATCTCTTGTGAATG

ACCCCGAGTTCTTCCTCGAATGGGAGAAACCGAATACATCCACTAGAGCCGACAAGTTTC

CCCTCTACCAACAGAAACCGGTAACGTTCACCGGCAAGGAGACCGAATCCCTCCACCAAT

GGAGGTCATTGCATCCGGGAGTCAACAACATGGTAACCACGCTAACATTTTTAAACTAAG

TGTGACTTCTTTAACATTTCAGAGACTTCCCGTGGCCAGCTAACTTTCCCCCTCTCCCAA

AATAAGGCATAAATTTTCTCTCAAATGATAAAAACTTCTTTTTAAAAAGAAAAAGACGGA

AAAAGGAAGTATCACCGAAACTTTCTATTGCTATTTGGGTGCCGCTAAAATGAACTCTAT

AAAACCAAGAAGAAAATCAATTACAGAACAACTTGCATTTTAAGGCTTCAATTTGCACCC

GACTGCAAGATTAAAGGATGCAAACACAAATTCGACTTTCCTATGAATAACTCTCAAACG

ATAGATTCACCCCTAAAGTTTGGTTTACGTTTACAGTGGTGCCAAATTCCGACCAACCGA

CCGACCAACGACGTCCAATCACATAACGGAGAGATCTATGTTTCTATACACTTTGCTTTC

CCAAGATTGTTTTTGCAGAAAGACCACCGCAAAATCACTCGAAATATCAACTCACTTCTT

AATTTACACTAGTACATTGACAAACTCTACAGTTGGCCGACTAACTTTCCAACCCATTAC

ACTTCAGAAGCTTCAATCAACATACAGACTCCCATCTTCAATAGTTAAAAAGAAATACCA

AAAAAAGAAATGATATCATACTAATTTTCAAATCCCTAAATCTAATCGCTGTCTAACTTA

TGTGGCAAGCTCATCCACTTCTGAATTAAAAACAAAACTGGGAAAAGTCGTCGTTATGTC

CCTGCAACCAGAAATATACAAAAATGAGTATATCAACCAGTAAGTTTTAATACTAGTAAA

TGAGATTTTGCGTGCGCAATTTGTCATCACATACCTCAAATAAGGAAGGGTCATGATCTT

CAACAAAGATGGGTTTTTAGAAACAAAGTTCTGCCATTCTGTTTTATCTATTTTGCCATC

CTGGTTTGCATCGGCTTCCAAGAAAGTCTGCTCAAGAAAGTAAGGACATTGTGAACTTTT

AGGCAATCTAGGAAAATGAAAACAAATGAAGAATACACAGAGACTGAAAATAATAGACCG

ACTGGAAATTACCATTGCCTTGCACTTATCATCCGCATTTACACCACAAATTAATCATCA

AATCTATCAGATTCATACAACTGAATAAATTACTACCAATGCTTCATGAGCAGAAAAGCA

ACAACAACATCAAACTTCTACTTAAAACTCCCAGGATATCCAACATAAGCCAGCATGTCA

TTTCTGCAATCTTGTAAAGGATACACATCCGCAAACTCAGATCAAGTTTTCTTGTAGATT

ACAAATGTTGACCAATAAGCTATGACTTGGTATTAGAATGAAAGATTCTAAATATCTGGG

CTGTCAAAAAATTCGAAGCACCTACCATGAAGAATAGCATTACATATATATTTGCTAAGA

ATGTTGATGCAAAACATGTTCGATTTGTTGGATTTTGAAGGCAGCGGATCTTACCTTATC

GAGTATTACTTCAATCGTTTCATCAGCCAGCTTCATTTCGGATTCACATAGAAGTGCAAT

CAGCATTTGCTTGACCTGCCATAATCACACAAATGAAGTTGCCACTCTGATATTTTTGAC

TTGCGACACAAAATATTAAACAGTACACCAACATACCTCTTCACGCTCGATATATCCTGT

ATTATCTTGATCATATAGCCTAAATGAGACTGGAGAAGACAAGCAAAGGTTAGATAAGTA

TGAATATACAACTTGAATTTTAAGCCATCTAATGTAAATATAACGCTTTCAGTCAACTTA

CAGTCAATCTTGTCCTCTTGAGAAGCATTTGGATGAAAAACATTTAGTGACCTAACAAAA

TCACTAAAATCAATGAGCCCCTTTCGCTTCACATCAAAAAGTTCAAAGAGCTGCATAACA

AGACTAGTTAGTCTAAATGTCTAATTAACGACCATGTACTTAAAGAATGATAGGAAACTC

AAGAGAAGATTATTAAAGATACCCTGTTGGCAAATAGATTTTCCTTCTTTCTGTTTTTGA

AGAGAGCCAACTGAAACTCTTCCTGCAAATAAGACACAAATTTTATATCAGATTTAGGTG

CATCCATGCAAATTATCCTGAATTCTTCACAATTACAACCCCATCCAGCAATCTCTGAAT

GTGGGATCACTTTCAGTTCAGAAAAAATTGAAGCCCTATATATGACCAAATACCAGGTGG

AGTTTGAAATTCAATATTCTGAAATCTTCCTTAACTAAACAAAATAACAGGACTATCTTA

CAATTAATTTGCGTTACCCATCATCAATAGACATAAAACTCAGCAACAAATAAAGAGCTT

AATCTAAGAAAGAACAGGGGGGGAATTAATATGCTCCCAGAAGAACACGTCTTTTAAATG

ATTTTCGTTTTTATACGGACTGATCTCAGATTAGCCCTGCTATCACATTCACTGTGGATT

TTTTTCAAATTATCATGTGTCCATGAACCAGATTATAACTACAGTAGATCATCTATGTTT

TAATTCATGTGTCACTTTGAGCCAACAACAATCACCACCCTTTTTTATCCATGCTCACAG

CCCAGTTCATGTTTCAATATGAACTTAACCAACAACAACAACGTTTACCAAGCTAAAATA

ACTGCAACTGTGGCTAGACAGATACAATCATTGGTGGACAACATACCTTGCTTATTAACC

CATCGTCAATGAGAGAACCGCTAATGCTCTTGAATAGCTCAAACAAGGCTTCAACTTCAC

TAACACTAACTGCAGATCCAAAACAGAGAACCAAATGAATTAACTTTCCAGGTTCAAACA

ACATGCAACAAGGGGATACATAGACAGTTATAATGATGCAATTAACAGGATGAATGGTGC

TATGACTTCAGAAGCCTTTTGCACTTTTTTCCCTATAAATTTAAATCAAAGAAAACATAA

ATCTGCATATTTTTAAAATCATAGATACGATCTACAAGGAAAAGTGAAGCGCCTGGACCA

CTGAAACAGAGTAATCAGATAAGAATTTCTACTGTGATTATAATACAGCATAAGAAATGA

TGACTAATAAACATAAAATACTTGTATCAAAATTTGATATTGGTAACAGGGGATAAGCAG

TTCTCATCTCAATGAAACAGTGGCTAGACAACAACTTACACGCAGTTTGCGAAGCCAGAA

CAACTGGGTCCTCATACCCAGGATGCTGCCTTGCTACCTTTGACTGAAAACACCCCATTT

ATCTACCAAAAAAACACAACAATGAAGAAGCCGAAAATACGCCTCGCTTAAACTCTTAGC

TTCTTCAACAAGATTATAGTATCACTATCTTCATAACCCTCAGCATCTTTAACACCTAGC

AATTTGCACAAAAAGAAAAGAGCCCGATTCAAACAGAATATTAATTTATTGACAGAGAAT

ATTTATGAAAACGACACCGGCGGGAACAAAAGCTATTTAACAAATACATATCAGAATCTA

CCAACAATGATAATCAAAACAGATGATATAGCACGTGGATTTTCAAGTTATCTAGAAACC

TCTTGAATTGCTTCTATAGTACTTGTACAGGGAAGAGATTCATTCAATTTTATCTTGTGC

TTCGTTTTTCTAGTTTCCTGCCTCTCGGAATTTTAAGAACGGGACAAAAGGAGAGAAGCA

GCGAAAAAATGAACCAACTCAACAGCGGATTGAAGATCTAGAATTGCTTTTATAATTATT

CACCTCCAATTTCTCGGGAAACAAACAGAGCAGAAAGTAAACGCAAATGAAATTCCAAGA

TCTTTTATTGCTATTATTCCTATTCGAAGTTGTAAACCAAAAAGATCACGTAAACACCTT

CCCCAGAAAACAACAAAAAGGTGAAAAAGAAAGAAAAACAAAAGATTAACCAAAAATGTC

GAGTTCTGCTCAGCACTTTGCTAGTAATAACTCTTAAGCATGAAAAGACACCGGTTCAAA

AGAAAGAAAAAGAAAAAGTAAGAACACAAAATCAAAAGCGTGAAAAGGAAGCGAGACGGA

GAGAAAGAGAAAAAAAGAACTTACCGACCAAAAACTTGGCCGGAAAATGCAAAGTCGGGG

CGACGATAAACAACCACTTTTCCCAAGATTGTTTGGCCTCTTAGAAAGAAAAGAAAGACA

GCAAAACAGAGAAGGTTTAGGTCGAGATTTAGCTGGGCTAGTGGAGAGATTTGTGGCCTG

TGTCTCTCGTCGAAGGAGAGAGAGAAAGAAAAACTTCTATGATTGATTGGGTAATCCAAC

GGGTGAGATTAAATCCGATGAGCGAACAGATTTTCCCGTATGTACAGTAGTACTAGTGTG

TATATATGGATTTGCATATCTGTACATTTTTTTGTCTTAAA

>NITAB02G1010 CHR2B 15519064 15523183

CTTCATGCTAAAATAATTTATTTATAAATATCTGAAAATAATCATTATTTTTGATATTCA

TTCGTTCATTCATTCTGCGGAAGAACAAACCTGCACTTTCTTCCATCTCTCAATCTTTTG

GTTTTCACTCATAAAGGTAATTTCTTTATTCATTCAATCACTGGGTTTCTCTACGTCAAG

CTATTTATATTTATATATATCTGTTTTTGTTTGGTTCCCGAGAAAACGAAAGAAAAACTT

TTTGTCAGGAACCGAACAGATCTTTTTTAGCCTCTGTTTTTTTAGCTAACGTTTGTTGCT

AGATCTGCTGATTTTTTGTTTCTTATGTTTAATCGAGTGAGTCGATATTCTGGAAATTGA

CGTTCAATTGGATGATTGGATCCTGGATCGGGTTCAATTGTTTGTTCAATTTCGATTTGT

GTTGTCGGTTTTGAATGTTTCGTTTGGATTGATAGTTCGCCTGAGCCTCGAGAAGCTGTG

ACGGTTTTCCATATTTTGAGAATTTCCTTGAATTATCCATCCGGAATTGTTGGTATATCG

TGTATGATTCAGTGTTTTGAGTGGTGCTTGGTCGTCTTGTCTGGTAGAAATTCTAGCTCA

GTTGCTTAAAGGCTACAGAAATAGTTGGTTTAGGTCTATGGTACAATCTTTTCACAATTC

TAGACATGATGTTATATTTGCTAGGTACTGTTATATTTCTTAGGTTTTTGTTTTCGAATA

TTGTGTTTGTCATACCTATAGAACTACATTCACAGTAGGAAGAGTTCTTGTCTGTTCATC

CACCTCGCTATGTGAGAATTGGATAATAAAACAGCTGAAAAAAGTGATGAAATCTGGTAT

ATTAATCTAGCTGCACAAAAGAAGGTTGATTGGCAGCAAGGTAGCAGGTACTATATTCAA

AGTAATCTTTTGATTTTAAACTATAAGGCGTGGCTGAGCCCAAAGTTGTTTCCTTAAACC

TTGACTTGTTATGTATGAGTTATTGTTCCTATACTATCGTTATGGCCATGTGCAGTATAT

ATTTCTATTTGATTTCATTTGATTATTTCCGAAGTTGGGAAATAGCTTCTGTATGTATAC

TGCTGCCATGTTGGGCACTTGTTGACAGTATGATTTCGCTTCGGAATGCAGAGATAGTCT

TCCCTATCAAAAAGGAAAATAAAAGAAAGAGAGAGACATTGTCTTGTGCACTCCTGCTTT

GACAAGTCTGGCAAGCGATTAAAGTTAGCACTTAGCAGCAGCTGGAAATGGTTAAAAGGT

TCAATTGTTTTCTTTCTCTGCAAATGGGATTCCTTTTCATAGTGTAACGATGAATTTTAT

TTAATTATTTCCTTTGTTCTACAGGTATGGAGTGTCTTGCTAAGCAGGAAATATCATGTT

GCAGTGCGTAGAGGGATTTAAGCATTTATTTGCTTCCCTATTGCAGTGCTGTGATCTTGA

TTTGTACAAACAATCAAGGGGCCTTGAAGATCCCGAAGTTCTTGCTAGAGAGACAGTTTG

TATGTTATCTACTTAATGTTGTTATAATGCTATTAAAGATATACTTATTCTAATCCATTG

TCGATGCTATAAAAAATTTGCTCAACAATATTAAGGATGTCTTAAAGATATACACTTGCA

TAGAACGTATTGTGAATTTTTAACAACGAATGACTCTTGTATTTTGTTAGTGGATACTGT

TGTTATATATTACTCCTGCTTAATTTTGTCCTTCAAAAACCCTGTATTTAAAGAACTATT

GAATTAATTTACTGAAATTGTTCCTTCACTAATATTAATGCAGTTAGTGTAAGTGAAATT

GAAGCACTTTATGAGCTGTTTAAGAAGATCAGCAGTGCTGTGATTGATGACGGGCTGATT

AACAAGGTTTGTGTGAATCTTCATATATATATATATATATATATATATATATATATATAC

ACACACACATATTTCAAATGTCTTTGTGCTAAATTTTCTTCATTAGATGATTTCATCTGT

TTTTCAGTTTCCATTATCATATGGTCAATACTTCTTTCATATTTTACCTTATAAGTTTTC

TTGAGTTTGTTTATTTTGTTATTCCCTTGCAGGAGGAGTTCCAATTGGCGTTGTTTAAGA

CGAACAAAAAAGAGAGCTTGTTTGCAGACCGGGTAAATTTATTGCTCCTCAAAACTTAGT

CTTCCATCTTATTATATCAGTTGCAACTTTTTTTCATTAGAGTACTTTGGCTTATCTGTT

TATGTGAGTTATTGTTATAGCAGTTCTTATTGATTGCAGGTCTTTGACTTGTTTGATACA

AAGCACAATGGAATTCTGGGTTTTGAAGAGTTTGCTCGTGCTCTCTCTGTCTTCCATCCA

AATGCTCCCATTGATGATAAAATTGAGTGTATGTTTGGCATTGCCTAATCTCTCAACATT

GTCATTTCTTTTAACTTCTTAATATTTTTGTTTGCTTGAGTACTGGGATGAAGTATTTGG

TCGTTACCTTTTGGCTTTTAATTTGCTTCTTTTCTTTTTATATCTCTTAATGATTTGATA

TTCTGGTGCAGTTTCTTTTCAGCTATACGATCTCAAGCAACAGGGTTTTATCGAGAGACA

GGAGGTAAATGTCTCAAATCTGGAGTGAAGTTCTGTTATCAGAGATATGGTTTTGTGACC

TTTCAATTATGCAATTCAACTATTAATTCACAAGTCCACATATAGGTGAAGCAAATGGTG

GTAGCTACACTTGCTGAGTCGGGTATGAACCTCTCAGATGATGTTATAGAAAGTATAATT

GACAAGGTGAGCTCCTCTATCCAATTAGATGTTTGAGTGATCTTTCTGAATCATTCTTCT

TTGTCATAAATGCCTTCTAAGTATGGAATAATTTTATATTCTCAACACTCTGAGCTTTGT

TTCAGACTTTTGAGGAAGCTGATACAAAACATGACGGGAAGATTGACAAGGAAGAATGGA

GAAACCTTGTCCTGAGACATCCATCTCTTCTCAAGAATATGACTCTTCAATACCTTAAGT

AAGTTGCAAATAAGTTATCTACATTCTTGGCTGGTGTATTATTCATACCCGATAAATTCT

CTTGCTAAGGTTATCTGATCGGAAACTATATTCAGTGTAGCTCATGTCTATAAGAAAACA

TGAACAAAGTCATAAGGACTGATGTTTTCTTGTAAACCTAACCTAGGAATAGGATAGCGC

ATTAAACAACATGAACAAAGTTATAAGGATTGATGTTATTATAACTAATGTCATATTGTC

GTTAGACCATAATTTAGCTTTTGATGGTGAAGTTTCTGAATACTAGCTATTGCTTGGCTC

TGATGGTGATTTGTTTGTTTATCTGAACAGGGACATCACCACAACTTTCCCAAGTTTTGT

ATTCCATTCACGAGTTGAAGATACCTGAACTCAAAATATGCAGATGAAGATCATAAGACA

TTAATTTTAAGTAAGGCCGGTTTTCTTTCTAGACGCAGTACCCCCTCTCTTATGTGAAAA

GCAGTTGCTCTTTTTTGGATGGATTTTGGTTGTTTGTATTCATATTCAAGTACTGAATCA

TCACTCTGGTATTCCTGGTTTTGAAGATCTTTGTGGAAATTGCAATTTGGTTTTGTGAAT

GCATTTTTAGTAAGTATGTAAAGAGCAATGAGCCAAAGAGAAGGGATCTTCGCTCTGCAA

GTGCAACAGCTCTAGTATCAATCTCGTTGATGTATGAGATGTTACTCAAACTGCTTTTAC

GATTATTTGATTATAAAACCTGCCGGCAACTTAAAGAAGTCGTGGCCTGGATTGGATACA

CACCACCCGTTAGTAAGAGAGCTTGATGTTGCAAACATCTTTTTGGTACATGAATTATAT

GTTGCCTTCCGCACATTGCCTCGTTATGATCTTCAAGTGCCGTGCGTTTTTATCCTTTTT

TGGTAATCTGTTAAGAGTCTGTTGAAACTTTTTTAGTATGGTATACGAGTCTACTTTTAC

AAAAAAAAAAAAAAAAAGCAATTAATTTGCATGACAAATTGGCGTGTAGCATAACAAGAA

GTGTCTGTCAATTGAACTATCAACTCGTGGTCAAGTTTTCTTGTTAGGAATGATATTAAT

GTTTTTGTTTTTATAAAGATGGCAGTAACGTTTTCTGACA

>NITAA02G0832 CHR2A 14740891 14744844

CTACATGCTAAAATAATTTATTTATATATATCTGAAAATTAATCATTGTTTTTTATTTTC

ATTCGTTCATTCATTCTGCAGAAGATCAAACCTGCACTTCTTTCCATCTCTCAATCTTTT

GGTTTTCACTCATAAAGGTAATTTATTTAATCATTCAATCACTGGGTTTCTCTGCGTCAA

GCTCTTTATATTTATGTATATCTGTTTTTGTTTGGTTCCCCAGAAAACGAAAGAAAAACT

TTTTTTGAGGAACCGAACTGATCTTTTTTAGCTAAGGTTTGTTGCTAGATCTGCTGATTT

TTTGGTTTTTATCTCTAATCGAGTAAGTCGATATTGTGGAAATTGACGTTCAATTGGATG

ATTGGATCCTGGATCGGGTTCAATTTCGATTTGTGTTGTCGGTTTTGAATGTTTCGTTTG

GATTGATAGTTCGCCTGAGCCTCGAGATATTGTGACGGTTTTCCGTAGTTTGAGAATTTC

CTTGAATTATCCATTCGGAATTGTTAGTATCTCGTGTATGATTCAGTGTTTTTTAGTGGT

GCTTGGTCGTCTTGTCTGGTTGAAATTCTAGCTAAGTTGCTAAAAGGCTGCAGAAATAGT

TGGTTTAGGTCTATGGTACTGTAATCAATCTTTTCACAATTCTAGACATGGTATTATTTT

TGCTGGGTACTGTTGTATTTCTTGGGTTTTTCTTTTCGAATTTTGTGTTTGTTATACCTA

TAGAACTACATTCACAGTAGGAAGAGTTCTTGTTTGTTCATCCATCTCGCTATGTGAGAA

TTGGATAATAAAACAGCTGAAAAAAGTGATGAAATCTGGTATACTTATCTAGCTTCCTTA

CGGCACAAAAGAAGGTTGATTGGCAATAAGGTAGCAGGTACTATATTCAAAGTTATCTTT

TGATTTCAAACTATAAGGCGTAGCTGAGCCCAAAGTTGTTTCGTTAAATCTTGACTTGTT

ATGTGTGAGTTATTGTTCCTATACTATTGTTATGGCCATGTGCAGTATATATTTCTAGGT

GATTTCATTTGATTATTTCCGAAGTTGGAAAATAGCTTCTATAGGTATACTGTTGCCATG

TTGGGCACTTGTTGACAGTATTATTTCGCTTCGGAATGCAGAGATAGTCTTCCCTATCAA

AGAGGAAAATAAAAGAAAGAGAGAGAGACATTGTCTTGTGCACTCCTGCTTTGACAAGTC

CGGCAAGTGATTAAAGTTAGCACTTAGCAGCAGCTGGAAATGGTTAAAAGGTACAATTGT

TTTCTTTCTCTGCAAATGGGATTCCTTTTCATGGTGTAATGATGAATTTTATTTAATTAT

TTCCTTTGTTCTACAGGTATGGAGTGTCTTGCTAAGCAGGAATTATCATGGTGCAGTGCA

TAGAGGGATTTAAGCATTTATTTGCTTCCCTATTGCAGTGCTGTGATCTTGATTTGTACA

AACAATCAAGGGGCCTTGAAGATCCTGAAGTTCTTGCTAGAGAGACAGTTTGTATGTTAT

CTACTTAATGTTGTCATAGTGTTCTTAAATATATACTTAGTCTAATTCATTGTTGGTGCT

ATAAAAAAATTTGCTCAACAATATTAAGGATGTCTTAAAGATATACAATTTGATATACAC

TTGGATAGAACTTATTGTGAATTTTTAACAACGAATGACTGTTGTATTTTGTTAGTGGAT

ACTGTTGTTATATGTTACTCCTGCTTAATTTTGTGCTTGAAATCCCTGTATTTAAAGAAC

TATTGAATTAATTTACTGAAATTGTTCCTTCACTAATATTAATGCAGTTAGTGTAAGTGA

AATTGAAGCACTTTATGAGCTGTTTAAGAAGATCAGCAGCGCCGTGATCGATGACGGGCT

GATTAACAAGGTTTGTGTGAATCTTCATGCATTTTGAATGTCTTTGTGCTAAATTTTCTT

CATTAGATTATTGCTTCTGTTTTGTCAGTTTCCATTATCATATGGTCAATACTTCTTTCA

TATTTTACCTTATAAGTTTCTCGAGTTTGTTTATTTTGTTATTCCCTTGCAGGAGGAGTT

CCAATTGGCGTTGTTTAAGACGAACAAAAAAGAGAGCTTGTTTGCAGATCGGGTAAATTT

ATTGCTCCTCAAAACTTAGTCTTCCATCTTATTATATCACTTGCAACTTTTTTTTTTCAT

TAGAGTACTTTGGCTTATCTGTTTATGCGAGTTATTGTTATAGCAGTTCTTACTGATTGC

AGGTCTTTGACTTGTTTGATACGAAGCACAATGGAATTCTGGGTTTTGAAGAGTTTGCTC

GAGCTCTCTCTGTCTTCCATCCAAATGCTCCCATTGATGATAAAATTGAGTGTATGTTTG

GCATTGCCTAATCTCTCAACATTGTCATTTCTTTTAACTTTTTTAATATTTTTGTTTGCT

TGTGTGCTGGGATGAAGTATTTGGCCGTTACCTTTCAGCTTTTAATTTGCTTCTTTTCTT

TTTATACCTCTTGATGATTGATATTCTGGTGCAGTTTCTTTTCAGCTATACGATCTCAAG

CAACAGGGTTTTATTGAGAGACAGGAGGTAAATGTCTCAAATCTGGACTGAAGTTCTGTT

ATCAGAGATATGGTTTTGTGACCTTTCAATTAGGCAATTCAACTTTTAATTCACAAGTCC

ACATATAGGTGAAGCAAATGGTGGTAGCTACACTTGCTGAGTCGGGTATGAACCTCTCAG

ATGATGTTATAGAAAGTATAATTGACAAGGTGAGCTCCTCTATCCAATTAGATGTTTGAG

TGATCTTTCTGAATCATTCTTCTTTGTCATAAATGCCTTCTACGTATGGAATAATTTTAC

ATTCTCAACACTCTGAGCTTTGTTTCAGACTTTTGAGGAAGCTGATACAAAACATGATGG

GAAGATTGACAAGGAAGAATGGAGAAACCTTGTCCTGCGACATCCATCTCTTCTCAAGAA

TATGACTCTTCAATACCTTAAGTAAGTTGCAAATAAATTATTTGCATTCTTGGCTGGTAT

ATTATGCATACCCGATAAATTCTCTTGCTAAGGTTATCTGATCGGAAACTATATTCAGTA

TAGCTCATGTCTATAAGAAAACATGAACAAAGTTATAAGGACTGATGTTTTCTTGTAAAC

CTAACCTAGGAATAGGATAGCGCATTAAACAACATGAACAAAGTTATAAGGACTGATGTT

ATTATAACTAATGTCATATTGTAGCTAGACCATAATTTAGTTTTTGATTGTGAAGTTTCT

AACTACTAGCTACTGCTTGGCTCTGATGGTGATTTGTTTGTTTATCTGACAGGGACATCA

CCACAACTTTCCCAAGTTTTGTATTCCATTCACGAGTTGAAGATACCTGAACTCGAAATG

TGCAGATGAAGATGATGAGACATTATTTTTAAGTAAGGCCGGTTTTCTTTCTAGACGCAA

TACCCCTCTCTTATGTGAAAAGCTTGGGCGAGAGTTTCTGCATTGTATTAGTTGCTATTT

TTTGGATGGATTTTGGTTGTTTGTATTCATGTTCAAGTACTGAATCATCACTCTCTGGTA

TTCCTGGTTTTGAAGATCTTTGTGGAAATTGCAATTTGGTTTTGCGAATGCATTTTTAGT

AAGTATGTAAACAGCAATGAGCCAAAGAGAAGGGATCTTCGCTCTGAAAGTGCAACAGCT

CTCTAATGGAGTATCAATCTCGGTGATGTATGAGATGTTACTCATACTGCCTTTACGATT

ATTTTGATCATAAAACCTACCAGCAACTTACGAAGTCGTGCCAGGATTGGATACACACCA

CCCTTTAGTAAGAAGCCTTGATTTTGCAAACATCTTTTCGGTACATGAATTATATGTTGC

CTTGCGCACATTGCCTAGTTATGAACTTCAAGTGCCGTGCTTTTTTTTTTTTTTTTTTTT

TGGGTGGGGGGGGGGGGGGGGGGGGGGTGGTAATCTGTTAAGAGTCGGTTGAAA

>NITAA02G2027 CHR2A 41041387 41047466

AAATCTTCTTATCATTTTGAGGTCAACACTTAATATACATAATTTCTCGTTCCAATCAAA

AGACGAAAAGAATCATAAACAGTTACAAGGGGACTCTAAAAGTAACTGTCTTGAGTATTT

TTTTTAAGTTTCCTCCAAACCAAGAAACAAGTTTTCTGCTACTAGAAAATAGTATATATA

TTATTATAACTTTAAAAGGTTCATACATGTCTAAAACCCTCGAAAAGGTCATCGTCTTTA

AAATGGAAACGGCCTCTAACAGTTCATTGCCATATACATATTTATTCACTAGTGAAAAAT

GAAAAAGAAAAATATACCTCTTAATTTGCAGTACACGAATCACAAAATTTCTTTTTACTG

TTTTCAACGAAAAGAACTCAAAACCAAAACATATGTACAACTGACTTGTGCGAAAGCTGC

GTGAACCCAAAACCCCAAAAACAAAAAATAGTCGGCCTTAAATACACGAAACTGAACACT

TTTGCGAGCTTAGATGTTCCGGGTCAGCTGTGATTGATGGAACCAGACTCTGAGAGTTCG

AGCTATCCTTGTTTAACCATGGATATAGCAAAAGCACCCACGGTTGAAACGGCCCCTTGG

TTCTCTGTCAAGAAATGGCATCTCGAGAAATGGCATCTTGGGATTGTTAGATATGGTAGG

AGGCTACACATCTGAATCCTCAACTTCAGAAGATAGAACAAAGCTTGGAAATGCCAAGGT

TATATCCCTATCGAAAGTTAAAAAGGGTGGAAAAAAAGGTAAATGATGAGAGTCGATATT

ACAATTTAATTCATGTAATTAATAAAACAAGATGAAAGGGAAGGTCTGTCATGAGACGAT

GTGTTACATGTGACTATGGGCTGACATGGCATCTTCTAATTGAAGATAGTCCTAAATCAA

ACAACCCAATCAAAGAATCTCAAGATGAAATAGCTTTTTAGTACTCACTTTAAATATGGA

AGAGTCATGTTCTTTATTAGAGAAGGATGCTTCGAGACAAACTCCTTCCATTCTTCTGGG

TCAATTTTTCCATCACCTTTAGTATCAGCTTCTTTGAATGTCTGCAGGAAATGACGCAAA

AGTCAGAATTTTCAATGAAATAAGCATAAAAGAACATAAAATAATCAACAATGCCAAGAT

ATGCGCACCTGATCCACAATAGTTTCAATGTATTCATCTGAGAGTACTAAATCTGATTCA

TGCAAAAGCGCCAATACCATCTCCCTCAACTGTCAAGCATTACAAAAACGTAAGCACAAA

CATAGTACAAAGGTGTTATGAAATGTTTTCCTGAACATGTTTAGTCCTTTTGATTCTCGA

CACGCAGTTGGAGAATGCCACCAACGATTCTTAAGAATACTTTTAACTGTTTTTCCTGTT

TTGGTTTTACAAAGTACTTTATAAAGAAAAGTTTGTATGACAATATCAAATCGGTAAAAG

AACAGGCTCTCAGGACTCTTAAAGAACAACAGAGTAAAGAACGGCAGCAGTAACTGCATA

CCTCCTCCCGCTCGATAAAGCCGGTTTGTCTTAAATCATACAATCTAAAAGCAACTGCAA

CCAGAAAAGAGAGAACAAAGATCAGTTAGAAGCATTGAATAATTGGAAAAGCCTTAACAT

GAAGAGTAAAGGATTACTCAAAGAGCATAATAAATTGGTAGAGAAATTAATCTTACATGC

TATTTTATCCTCAACACGCGCATTTGGATGGAATACTCCCAAGGATCGAACGAATTCCCC

AAACTCAATAACCCCATTGCGTTTGGCATCAAAAAGATCAAAAATCTAAAAAACACGCAA

AAAAGCTATATCAATATAAACCAGAAGCAAAGACTACCATATTCTATTGCTGCGAAAGCA

CAAGATGCTCTTCACATTTAAAATAAATACTAATACATACCCTGTCAGCAAAAAGATTCT

TCTTTCTTCTGTTTCTAAAGAGTGCAAATTGGAATTCTTCCTGCTCACAATTCACATAAA

AAGTTAGATCCAAATAAATATGAACTAAAAAAATATGAATAATACAAAATTATTAAATTG

TGACTCCAGATCAACATTCAAAATACCTTGTGAATGAGCCCATCATGGAATATTGAATTG

CTTAATTTCTTGAAAAGCTCATGCAAGGCTTCTACTTCACTAACTGTAACTGCAATGAAA

AGAAAATATATCTTTAACCCCAATAATCATTTTACAATACATCATTAGCTGGATATAAAC

AGGCCACAAACTATCTATTACTATATCTTTCATGCCACAACTACCCTATTCATAACCACA

CTCTCCAATTTCTTAGTGTACAATTACGCCACACAAGGTCACACCTGTTGTCAGCAAAGA

TTAGAAATTCGATAAAGTCGTGATTGCTCCTAAAAGCATCCTACAGACAATAGTCAAAGG

TAGGTCTCTTTTACGGTTCACTATTATCAGTAGTGCCTGTCTTAATAGCACCTTTCTTGG

TGATGGTTCTACATATAAGCACTCTGTTCCTTAGCCATAAATTAAGAGTGCCTTTAGCAA

TACATATATATATGCGTGTGTGTGTATGCATAGCTTAAGTTTTATTAGACAATATAAGAA

TGTAACAAAATATCAAACTACCTGCAAAAGTAGTTCATTTTCTTTTCTAAAGAACCAAAT

ATCTTTTTGTTCTTTTTCCGATCACAACTTGAAGTAATTTTTTTAATACAGAAGACATTT

TTCTAAGCCAAAAGAAATTAGTTGAAAAGAATCCATGGTCAGTGAACTTTTTGTGGTGAC

AAAAAGCTGTACTACCAGAGATTACTTTTCAATTAATCTAAAGAGTATCAACCTCAAGTC

CTTCCCCTCGCAGCACCATGGGAAGGCCATATCTTGAACATGGTCCATCATCAAGTAAAA

CTATCTACAATTCCACTTAATAAAGATTATTCAAAGTGCAGAATTCTTGGGTTATGTACC

ATTTTAGTTAAAGGTAGAAAACACACATCACTGTTAATAAGCTGAGAAGCACCAATGAAA

CTTTAATAATAAATCATAGGCAAAATATGTATAAAGTGAAAGTCAAAAGAATTGTACAGG

GGCGGGGAGATGACTGACAAGGTGTTACAGCAGCAAGAACGGTAGGATCCTCATAACCAA

GTGTTTGTTTCCTTATCTTCGAGCACTTACAACCCATCAGATGAACTAGCGTAATGTTTT

CACAATATACATAAATGGTAGTTCCTTCAAAAATTCTACCTGCCAAAAACCAGGATGAAT

GCATACCATCAGAAAACATAAACATATGGAAATACAGAATTTGCAGAAAATTACATGTTT

AGTACATCAACAGAGAAATCGAGTAGCATTTGTAAGATTAAATATAGCATATCATCTAAA

TCCATGAACATCGTCAGGATGTTCAAGATGAACAATGAAAACAGCACTGAACATGAAAGC

GTACTTGTTCAAATAAATAAAGGCAAACCAAATGAATATGAACAAGGGATTAGTTGTATT

CAGTACATGTAATGAATAAAGCTGTAATCAATCTGTTCCATAAATTGGAATAAGGCGACC

TACAGGAAAGTACCAATTCTAAAGAAATAATATCTATTCCATTGTGCAGAAAACAATAAA

TGACAATGATTGATATAGCATAGATACCCCTACAAGTCTATGACACTTGCAATAACATAA

GTACAACACGTATAAAAAAACTTTAGTATACATGTCCTTGGACATCATAATTGATGGTAA

TACAATTATATGAGGATCATAGTAATAGACAGTCCATGACTTAGCTTAAAGCTCACTATT

TCAGACCCTCTTATCATCTGGTAGATGAACAGACTCTAAAGGTCATCTGATTGCAGGTAA

AGAGATGTCAAGCGATAAATAATGTCTTCTTAAGAAAGAAGAAAGACAGTGACAAAAGAA

GTTTCGTATACTAGAAAAACCCAACTAATACATACTATATATGGTCCTAGTTCCCCACTA

ATGGACACATATGTTACCATCATGGAACCGGCCAGCTTAGCAAATGGCGTGTGACATTAC

AGACAAATAAGTGAAAGAAGTTGTAGTGGAAACTTCAGATACTTCTAACAGCACAATAAT

TGTCGAATTCAAAAAGCATTATGTAAGCAGAAATGACCTTTCCCGGTTATATCCAAAAGG

ACGGGATGCTCCAAGATGGAACTCTTAGTTGGACAGATTAAACAAATATTACAGTAAAAG

ACATGACAAATTCGCCCACATTTTTCTGAAAGAAACACAATTTTAACAAGCTATTCACAA

CTTGAGCTCCATCTGGATGTATAAACAACCGCAAAAATACCAATTTCTTACTATTTTTCT

CCAAATTTTCCAGTACCATACACCAAATTCTAGTAAAACAAATTGTCCAATATGCCGGAG

AAAACCACTAAAGAAGAACGCACACATATGATCCTCACACTCCAAATAGACCACATGTTC

TGTGTGATTAAGCAAAAGAAACTTTTCCACCAAACAATAATTTTTAAAGCAAATAAACAA

ATACATGGCAGAGAAAAATAGTGAATTAAAAATTTCAAAAACTTCCAACGACACCAAATT

TTAGAGAAGAAAAGAACACAAACTACCAAGAAAGCATATGCAAGCAGATTTGACCTCATA

GATTGATAAGTTTTCCAAAACTTCAGATATACACAAGGGCTGAAGTGATCCTTAGGCATT

TATTAACTATAGAGATGATTAAATTATTATGATGAAAACTTAAGGCCTACCTAATACCTC

CCACTCTCTGTTTTTGGCTGTTAAAGCATTTAATTAGTTGGGACCGAGTTGAATCATTTG

CAATAAATTAATTCTACAAATTTTCCTAAGTTACTTAAAAGAGAATATATTGTATCAGAT

AAATTTTAAGCACATACGACAAAAAATCTAGCAATTGGACCATAAAATTTTCTTCGCATT

CAAGGGGAAAAAAATATGGAAAATCTTATCTCCAAGGAATGAAACCCGTACAAGGAAGAC

TACGAGATTTTCTTATGGTGATTTCCATAAATGAGATGACATACAGAAAGAAGGTAAAAA

AACTAACACATCAAGGTTAATGATTTTATCATCTAGAGAAATTTCATGTTTCTAGGTCGT

TTTGGGACCACAGATCTGACAACTCAAGATAAGCTAATCATATTCTGCAAATTAAGGCCC

ATTTCCTTTGTTTCATTTTTTAAGTTGATTTTCTCGGCAACCAAACAGACCGCTTACCAG

TTTCTGTATTCAATGTACGTATATTTCAAGAAAACTACTCCGATGATGATATTTATTTTT

TAGACATACGTTAAAGTGAATTTACGATCGGCGGCGGAAAGAAAAGGAACGAAAATTAAG

GATTTCTGACTTGAGCAAGATACTCGGCGACGTAATCCATCTTTCTTGTTCGGCAACCGA

GAAAATGCAGGAAAGAATAAGAAATTAAAAAATATTCTAGTTCATGAGATTGTTAGTCCA

ACTTAAGAATTAACCGATCGGCTAAAACTTTGTAAGACGCGATAAACTTGAGATCTAAGA

CGAACTCGTCTTTTTTCCTCAACTTTTCCCGGCAACCAGCAGATACCTCAGCACATCCCC

GGATTCACCGACCAAAAAAAAAAAAAGGAAAACACAAACAGAGAATCCAAGAAAGAAAAC

CTCCAAATGAGAACCGAACTGACCTGCGCAAATGCCTGATTCCTGATGCTCCGGCCAGAA

AAAGAAAAGACAAAGCAGCAAAAATGGCTTAAATAAAATCTACAACGCGAGTTGTGGTTA

TTGTTTTTGCAGAAAATAATCAAAACTGGAGAATAGAGAGAATAGAGGAGAAGAAGAAGA

AGAAGAGAACTGTAGTCCCTGTAAAAATCTATTAAAGCTTCCTTCTAAGCTTCAATGAAA

TCTCTATTATCCCGCTTTGTTAATGAAGCTACATTTATATATATGTATATGTGTGTATGT

ACGCCAAAGTTAAATTTTGTCTTGCCTATTTTTAATATTATTAGGGATAATTAAACTTTA

GTCCCAAAAAGAAAATTTAATTGTATTTTAATCTCTATAATTTTAGAAAATTGTACTTTT

ATGTCTTATGTCTGTTAAAA

>NITAA02G2024 CHR2A 40867594 40873675

AAATCTTCTTATCATTTTGAGGTCAACACTTAATATACATAATTTCTCGTTCCAATCAAA

AGACGAAAAGAATCATAAACAGTTACAAGGGGACTCTAAAAGTAACTGTCTTGAGAATTT

TTTTGAAGTTTCCTCCAAACCAAGAAACAAGTTTTCTGCTACTAGAAAATAGTATATATA

TTATTATAACTTTAAAAGGTTCATACATGTCTAAAACCCTCGAAGAGGTCATCGTCTTTA

AAATGGAAACGGCCTCTAACAGTTCATTGCCATATACATATTTATTCACTAGTGAAAAAT

GAAAAAGAAAAATATACCTCTTAATTTGCAGTACACGAATCACAAAATTTCTTTTTACTG

TTTTCAACGAAAAGAACTCAAAACCAAAACATATGTACAACTGACTTGTGCGAAAGCTGC

GTGAACCCAAAACCCCAAAAACAAAAAATAGTCGGCCTTAAATACACGAAACTGAACACT

TTTGCGAGCTTAGATGTTCCGGGTCAGCTGTGATTGATGGAACCAGACTCTGAGAGTTCG

AGCTATCCTTGTTTAACCATGGATATAGCAAAAGCACCCACGGTTGAAACGGCCCCTTGG

TTCTCTGTCAAGAAATGGCATCTCGAGAAATGGCATCTTGGGATTGTTAGATATGGTAGG

AGGCTACACATCTGAATCCTCAACTTCAGAAGATAGAACAAAGCTTGGAAATGCCAAGGT

TATATCCCTATCGAAAGTTAAAAAGGGTGGAAAAAAAGGTCAATGATGAGAGTCGATATT

ACAATTAAATTCATGTAATTAATAAAACAAGATGAAAGGGAAGGTCTGTCATGAGACGAT

GTGTTACATGTGACTATGGGCTGACATGGCATCTTCTAATTGAAGATAGTCCTAAATCAA

ACAACCCAATCAAAGAATCTCAAGATGAAATAGCTTTTTAGTACTCACTTTAAATATGGA

AGAGTCATGTTCTTTATTAGAGAAGGATGCTTCGAGACAAACTCCTTCCATTCTTCTGGG

TCAATTTTTTCATCACCTTTAGTATCAGCTTCTTTGAATGTCTGCAGGAAATGACGCAAA

AGTCAGAATTTTCAATGAAATAAGCATAAAAGAACATAAAATAATCAACAATGCCAAGAT

ATGCGCACCTGATCCACAATAGTTTCAATGTATTCATCTGAGAGTACTAAATCTGATTCA

TGCAAAAGCGCCAATACCATCTCCCTCAACTGTCAAGCATTACAAAAACGTAAGCACAAA

CATAGTACAAAGGTGTTATGAAATGTTTTCCTGAACATGTTTAGTCCTTTTGATTCTCGA

CACGCAGTTGGAGAATGCCACCAACGATTCTTAAGAATACTTTAACTGTTTTTCCTGTTT

TGGTTTTACAAAGTACTTTATAAAGAAAAGTTTGTATGACAATATCAAATCGGTAAAAGA

ACAGGCTCTCAGGACTCTTAAAGAACAACAGAGTAAAGAATGGCAGCAGTAACTGCATAC

CTCCTCCCGCTCGATAAAGCCGGTTTGTCTTAAATCATACAATCTAAAAGCAACTGCAAC

CAGAAAAGAGAGAACAAAGATCAGTTAGAAGCATTGAATAATTGGAAAAGCCTTAACATG

AAGAGTAAAGGATTACTCAAAGAGCATAATAAATTGGTAGAGAAATTAATCTTACATGCT

ATTTTATCCTCAACACGCGCATTTGGATGGAATACTCCCAAGGATCGAACGAATTCCCCA

AACTCAATAACCCCATTGCGTTTGGCATCAAAAAGATCAAAAATCTAAAAAACACGCAAA

AAAGCTATATCAATATAAACCAGAAGCAAAGACTACCATATTCTATTGCTGCGAAAGCAC

AAGATGCTCTTCACATTTAAAATAAATACTAATACATACCCTGTCAGCAAAAAGATTCTT

CTTTCTTCTGTTTCTAAAGAGTGCAAATTGGAATTCTTCCTGCTCACAATTCACATAAAA

AGTTAGATTCAAATAAATATGAACTAAAAAAAATATGAATAATACAAAATTATTAAATTG

TGACTCCAGATCAACATTCAAAATACCTTGTGAATGAGCCCATCATGGAATATTGAATTG

CTTAATTTCTTGAAAAGCTCATGCAAGGCTTCTACTTCACTAACTGTAACTGCAATGAAA

AGAAAATATATCTTTAACCCCAATAATCATTTTACAATACATCATTAGCTGGATATAAAC

AGGCCACAAACTATCTATTACTATATCTTTCATGCCACAACTACCCTATTCATAACCACA

CTCTCCAATTTCTTAGTGTACAATTACGCCACACAAGGTCACACCTGTTGTCAGCGAAGA

TTAGAAATTCGATAAAGTCGTGATTGCTCCTAAAAGCATCCTACAGACAATAGTCAAAGG

TAGGTCTCTTTTACGGTTCACTGTTATCAGTAGTGCCTGTCTTAATAGCACCTTTCTTGG

TGATGGTTCTACATATAAGCACTCTGTTCCTTAGCCATAAATTAAGAGTGCCTTTAGCAA

TACATATATATATATGCGTGTGTGTGTATGCATAGCTTAAGTTTTATTAGACAATATAAG

AATGTAACAAAATATCAAACTACCTGCAAAAGTAGTTCATTTTCTTTTCTAAAGAACCAA

ATATCTTTTTGTTCTTTTTCCGATCACAACTTGAAGTAATTTTTTTAATACAGAAGACAT

TTTTCTAAGCCAAAAGAAATTAGTTGAAAAGAATCCATGGTCAGTGAACTTTTTGTGGTG

ACAAAAAGCTGTACTACCAGAGATTACTTTTCAATTAATCTAAAGAGTATCAACCTCAAG

TCCTTCCCCTCGCAGCACCATGGGAAGGCCATATCTTGAACATGGTCCATCATCAAGTAA

AACTATCTACAATTCCACTTAATAAAGATTATTCAAAGTGCAGAATTCTTGGGTTATGTA

CCATTTTAGTTAAAGGTAGAAAACACACATCACTGTTAATAAGCTGAGAAGCACCAATGA

AACTTTAATAATAAATCATAGGCAAAATATGTATAAAGTGAAAGTCAAAAGAATTGTACA

GGGGCGGGGAGATGACTGACAAGGTGTTACAGCAGCAAGAACGGTAGGATCCTCATAACC

AAGTGTTTGTTTCCTTATCTTCGAGCACTTACAACCCATCAGATGAACTAGCGTAATGTT

TTCACAATATACATAAATGGTAGTTCCTTCAAAAATTCTACCTGCCAAAAACCAGGATGA

ATGCATACCATCAGAAAACATAAACATATGGAAATACAGAATTTGCAGAAAATTACATGT

TTAGTACATCAACAGAGAAATCGAGTAGCATTTGTAAGATTAAATATAGCATATCATCTA

AATCCATGAACATCGTCAGGATGTTCAAGATGAACAATGAAAACAGCACTGAACATGAAA

GCGTACTTGTTCAAATAAATAAAGGCAAACCAAATGAATATGAACAAGGGATTAGTTGTA

TTCAGTACATGTAATGAATAAAGCTGTAATCAATCTGTTCCATAAATTGGAATAAGGCGA

CCTACAGGAAAGTACCAATTCTAAAGAAATAATATCTATTCCATTGTGCAGAAAACAATA

AATGACAATGATTGATATAGCATAGATACCCCTACAAGTCTATGACACTTGCAATAACAT

AAGTACAACACGTATAAAAAAACTTTAGTATACATGTCCTTGGACATCATAATTGATGGT

AATACAATTATATGAGGATCATAGTAATAGACAGTCCATGACTTAGCTTAAAGCTCACTA

TTTCAGACCCTCTTATCATCTGGTAGATGAACAGACTCTAAAGGTCATCTGATTGCAGGT

AAAGAGATGTCAAGCGATAAATAATGTCTTCTTAAGAAAGAAGAAAGACAGTGACAAAAG

AAGTTTCGTATACTAGAAAAACCCAACTAATACATACTATATATGGTCCTAGTTCCCCAC

TAATGGACACATATGTTACCATCATGGAACCGGCCAGCTTAGCAAATGGCGTGTGACATT

ACAGACAAATAAGTGAAAGAAGTTGTAGTGGAAACTTCAGATACTTCTAACAGCACAATA

ATTGTCGAATTCAAAAAGCATTATGTAAGCAGAAATGACCTTTCCCGGTTATATCCAAAA

GGACGGGATGCTCCAAGATGGAACTCTTAGTTGGACAGATTAAACAAATATTACAGTAAA

AGACATGACAAATTCGCCCACATTTTTCTGAAAGAAACACAATTTTAACAAGCTATTCAC

AACTTGAGCTCCATCTGGATGTATAAACAACCGCAAAAATACCAATTTCTTACTATTTTT

CTCCAAATTTTCCAGTACCATACACCAAATTCTAGTAAAACAAATTGTCCAATATGCCGG

AGAAAACCACTAAAGAAGAACGCACACATATGATCCTCACACTCCAAATAGACCACATGT

TCTGTGTGATTAAGCAAAAGAAACTTTTCCACCAAACAATAATTTTTAAAGCAAATAAAC

AAATACATGGCAGAGAAAAATAGTGAATTAAAAATTTCAAAAACTTCCAACGACACCAAA

TTTTAGAGAAGAAAAGAACACAAACTACCAAGAAAGCATATGCAAGCAGATTTGACCTCA

TAGATTGATAAGTTTTCCAAAACTTCAGATATACACAAGGGCTGAAGTGATCCTTAGGCA

TTTATTAACTATAGAGATGATTAAATTATTATGATGAAAACTTAAGGCCTACCTAATACC

TCCCACTCTCTGTTTTTGGCTGTTAAAGCATTTAATTAGTTGGGACCGAGTTGAATCATT

TGCAATAAATTAATTCTACAAATTTTCCTAAGTTACTTAAAAGAGAATATATTGTATCAG

ATAAATTTTAAGCACATACGACAAAAAATCTAGCAATTGGACCATAAAATTTTCTTCGCA

TTCAAGGGGAAAAAAATATGGAAAATCTTATCTCCAAGGAATGAAACCCGTACAAGGAAG

ACTACGAGATTTTCTTATGGTGATTTCCATAAATGAGATGACATACAGAAAGAAGGTAAA

AAAACTAACACATCAAGGTTAATGATTTTATCATCTAGAGAAATTTCATGTTTCTAGGTC

GTTTTGGGACCACAGATCTGACAACTCAAGATAAGCTAATCATATTCTGCAAATTAAGGC

CCATTTCCTTTGTTTCATTTTTTAAGTTGATTTTCTCGGCAACCAAACAGACCGCTTACC

AGTTTCTGTATTCAATGTACGTATATTTCAAGAAAACTACTCCGATGATGATATTTATTT

TTTAGACATACGTTAAAGTGAATTTACGATCGGCGGCGGAAAGAAAAGGAACGAAAATTA

AGGATTTCTGACTTGAGCAAGATACTCGGCGACGTAATCCATCTTTCTTGTTCGGCAACC

GAGAAAATGCAGGAAAGAATAAGAAATTAAAAAATATTCTAGTTCATGAGATTGTTAGTC

CAACTTAAGAATTAACCGATCGGCTAAAACTTTGTAAGACGCGATAAACTTGAGATCTAA

GACGAACTCGTCTTTTTTCCTCAACTTTTCCCGGCAACCAGCAGATACCTCAGCACATCC

CCGGATTCACCGACCAAAAAAAAAAAAAGGAAAACACAAACAGAGAATCCAAGAAAGAAA

ACCTCCAAATGAGAACCGAACTGACCTGCGCAAATGCCTGATTCCTGATGCTCCGGCCAG

AAAAAGAAAAGACAAAGCAGCAAAAATGGCTTAAATAAAATCTACAACGCGAGTTGTGGT

TATTGTTTTTGCAGAAAATAATCAAAACTGGAGAATAGAGAGAATAGAGGAGAAGAAGAA

GAAGAAGAGAACTGTAGTCCCTGTAAAAATCTATTAAAGCTTCCTTCTAAGCTTCAATGA

AATCTCTATTATCCCGCTTTGTTAATGAAGCTACATTTATATATATGTATATGTGTGTAT

GTACGCCAAAGTTAAATTTTGTCTTGCCTATTTTTAATATTATTAGGGATAATTAAACTT

TAGTCCCAAAAAGAAAATTTAATTGTATTTTAATCTCTATAATTTTAGAAAATTGTACTT

TTATGTCTTATGTCTGTTAAAA

>NITAB02G2334 CHR2B 45889259 45895357

AAATTATCTTATGATCTTGAATTCAACACTTAACATACATAATTCGTCATTCCAATCAAA

AGACAAAAAGAATCATAAACAATTACAAGGGGTTAAGTGTCTATGTATTTTCAAGTTCTC

TCCGAACCAAGAAACAAGTTTTCCGCTACTAGAAAATAGTATATATATAATATTATAACT

TTAAAAAGTTCATACATGTCTAAAACCCTCGAAGAGGTCATTGTCTTTAAAATGGAAACG

GCCTCTAACAGTTTATTGCCATATACATATTTATTCACTAGTGAAAAATGAAAAAGAAAA

ATATACCTCTTAATTTGCAGTACACCAATCACAAAAAATTTTTTTTTACTGTTTTAAACG

AAAAGAACTCAAAACCGAAACATATGTACCACTGACTTGTGCGACCGCGGCGTGAACCCA

AAACCCCAAAAACAAAAAATAGTCGGCCTTAAATACACGAAACTGAACACTTTTGCGAGC

TTAGATGTCCCGGGTCAGCTGTGATTGATGGAACCAGACTCTGAGAGTTCGAGCTATTCT

TGTTTAACCATAGATATAGCAAAAGCACCCACGGTTGAAACGGCCCTTTCGTTCTCTGTC

GAGAAATGGCATCTTGGTATTGTTAGATATGGTAGGAGGCTACACATCTGAATCCTCAAC

TTCAGAAGATAGAACAAAGCTTGGAAATGCCAAGGTTATATCCCTGTCGAAAGGAACAAA

AACAAAAAAAGGAAAAAAAAGGTAAATGATGAGAGTCGATATTACATTTTAATTCATTAA

GTTAATAAAACAAGATGAAAGGGAAGGTCTGTCATGAGACGGTGTGTAACATGGGACTAT

GTGCTGACATGGCATCTTCTAATTGAAGATAGTCCTAAATCAAAGAATCTCAAGATGAAA

TAGCTTTGTTGTACTCACTTTAAATATGGAAGAGTCATGTTCTTTATTAGAGAAGGATGC

TTCGAGACAAACTCCTTCCATTCTTCTGGGTCAATTTTTCCATCACCTTTAGTATCAGCT

TCTTTGAATGTCTGTAGGAAATGACGCAAAAGTCAGACTTTTCAGTGAAATAAGCATAGA

AGAACATAAAATAATCAACAATGCCAAGATGTGCGCACCTGATCCACAATAGTTTCAATG

TATTCATCTGAGAGTACTAAATCTGATTCATGCAAAAGCGCCAAAACCATCTCCCTCAAC

TGTCAAGCAGTACAAAAACGTAAGCACAAACATAGTACAAGCGTGTTATCCTGAACATGT

TTAGTCCTTTGGATTCTCGACACGCAGTTACCGGAAAATGCCACTAACGATTCTTAAGAA

TACTTTTAACTGTTTTTACTGTTTTGGTTTTACAAAGTTACTTTATAAAGAAAAGTTTGT

ATGACAAGATAAAATAGGCAAAAGAACAGGCTTTCAAGACTCTTAATGGAAAACAGAGTA

AAGAACAGCAGCAGTTACTGCATACCTCCTCTTGCTCGATAAAGCCGGTTTGTCTTAAAT

CATACAATCTAAAAGCAACTGCAACCAGAAAAGAGAGAACAAAGATTAGTTAGAAGCATT

GAATAATTGGAAAAGCTTTAACATGAAGAATAAAGGATTTACTCAAACAGCATAATAAAT

TGGTAGAGAAATTAATCTTACATGCTATTTTATCCTCAACACGCGCATTTGGATGGAATA

CTCCCAGGGATCGGACGAATTCCCCAAACTCAATAACCCCATTGCGTTTGGCATCAAAAA

GATCAAAAATCTACAAAACACCCAAAAAAGGCTATATCAATATAAGCCAGAAGCAAAAAC

TACCATATTCTATTGCTGCAAAAGCACAAGATGCTCTTCACATTTAAAATAAATACTAAT

ACATACCCTGTCAGCAAATAGATTCTTCTTTCTTCTGTTTCTAAAGAGTGCAAAATGGAA

TTCTTCCTGCTCACAATTCACATAAAAAGTTAGATTCAGACAAATATGAACTAAAAAGAT

ATGAATAATACAAAATTATTAAACTGTGACTTCAGATCAACATTCAAAATACCTTGTGAA

TGAGCCCATCATGGAATATTGAATTGCTTAATTTCTTGAAAAGCTCATGCAAGGCTTCTA

CTTCACTAACTGTAACTGCAATGAAAAGAAAATATATCTTTAACCCCAATAATCATTTTA

CAATACATCATTAGCTGGATATAAACAGGCCACAAACCATCTATTACTATATCTTTCATG

CTACAACTACCCTATTCATAACCACACTCTCCAATTTCTTAGTGTACAATTATGCCACAC

AAGGTCACACCTGTTGTCAGCGAAGATTAAAAATTCGATAAAGTCGTGATTGCTCCTAAA

AGCATCCTACAGACAATAGTCAAAGGTAGGTCTCTTTTATGGTTCACTATGTTCAGTAGT

GCCTGTCTTAATAGCACCTTCCTTGGTGATGGTTCTACATATAAGCACTCTGTTCTTTAG

CCATAAATTAAGAGTGCCTTTAGTTATACATATGTGTGTGTGTGTGTGTGTATAGCTGAG

GTTTTATTAGACACTATAAGAATGTAACAAAATATCAAACTACCTGCAAAAGTAGTTCAT

TTTCTTTTCTAAAGAACAAATATCTTTTTCTTCTTTTTCTGATCACAACTTGAAGTAATT

TTTTAATACAGAAAACATTTTCTAAGCCAACCAAAATCAGTTGAAAAGAGTCCTGGTTTC

AATTTAAGTGAAGAACTTTTTGTGGTGACAAAAAGTTGTACTACCACAGATTAATTTTCA

ATTAATCTAAAGAGTATCAACCTCAAGTCCTTCACCTTTCGGCACCATGGGAAGGCCATA

TCTTGAACATGGTCCATCATCAAGTAAAACTATCTACAATTCCACTTAATAAAGATTATT

CAAAGTGCAGAATTCTTGGGTTATGTACCATTTTAATTAAAGGTAGAAAACACACATCAC

TGTTAATAAGCTGAGGAATTTAATATTGGTTCATAAGAAGCACCAATGAAACTTTAATAA

TAAATCACCGGCAAAATATGTATTAAGTGAAAGTCAAAAGAATTGTACAGGGGCGGGGAG

ATGACTGACAAGGTGTTACAGCAGCAAGAACGGTAGGATCCTCAAAACCAAGTGTTTGTT

TCCTTATCTTCGACCACTTACAACCCATGAGATGAACTAGCGTAATCTTTTCACAATATA

CATAAATGGTAGTTCCTTCAAAAATTCTACCTGCCAAAACCCAGGATGATGCATACCGTC

AGAAAATATAAACATATGGAAATAAAGAAATTTGCAGAAAATTACATGTTTAGTACATCA

ACAGAGAAATCGAGTAGCATTTGTAAGATTAAATATAGCATATCATCTAAATCCATAAAC

ATAGTCAGGATGTTCAAGATGAACAATGAAAACAGCAATGAACACTAAAGAGTACTTGTT

AAAATAAATAAAGGCAAACCAAACGACAATGAACACAAGGGATTACTTGTATTCAGTTAT

GTAATGAATAAAGCTGTAATAAATCTGTTCCATAAACTGAAATAAGCCAACCTACAGGTC

CGTACCAGTTCTAAAGAAATAATATCTGTTCCACCGGGCAGAAAACAATAAATGACAATG

ATTGATATAGAATAGATACCCCTACAAGTCTATGACACTTGCAATAACATAAGTACAACA

AGCATAAAAAAAACTTTAGTATACGTGCCCTTGGACATCATAATTGATGGTAATACAATT

ATTTGAGGATCATAGTAATATACGGTCCATGACTTTGCTTAAAGCTCACTATTTCAGACC

TTCTTATCATCTGGTAGATGAACAGACTCTAAAGGTCATCCGATTGCAGGTAAAGAGATG

TCAAGTAATAAATAATGTCTTCTTAAGAAAGAAGAAAGACAGTGACAAAAGAAGTTCCGT

ATACTAGAAAAAGCCATATGCAACTAATATATACTATGTATGGTCCTAGTTCCCCACTAA

TGGACACATCTGCTACTATCATGGAACCAGCCAGCTTAGCAAAATGGCGTGTGACATTAC

AGACAAATAAGTGAAAGAAATTGTAGTGGAAACTTCAGATACTTCTAACAGCACAATAAT

TGTCGAATTCAAAAAGCATTATGCAAGCAGACATGACCTTTCCCGGTTAAATCCAAAAGG

ACGGGATACTCCAAGATGGAACTCTTAGTTGGACAAATTAAACCAATATTACAGTAAAAG

ACATAACAAATTCCCCCACAGTTTTCTGAAAGAAACACAATTTTAACAAGCTATTCACAA

CTTGAGCTCCATCTGTATATATAAACAACCGCAAAAATACCAATTTCTTACAATTTTTCT

CGAATTTTCCAGTACCATACACCAAATTCTAGTAAAACAAATTGTCCAATATGCCGGAGA

AAACCACTAAAGAAGAAAGCACGCATATGATCCTCCCACTCCAAATAGATCATATGTTTG

GTGTGATTAAGCAAAAGAGACTTTTCCATCAAACAATAATTTTTAAAGGGTATAAACAAA

AATATGGCAGAGAAAAATAGTGAATAAAAAATTTCAGAAACTTCCAACGACACCAATTTC

TAGAAAAGAATCGAACACAAACCACCAAGAAAGCATATGCAAGCAAATTTGACCTCATAG

ATAGATAAGTTTTCCAAAAAATCAGATATACACAAGGGCTGAAGTGATCTTCAAGCATTT

ATTAACTATAGAGATGATTAAATTATTATGATGAAAACTTAAGGCATACCTAATACCTCC

CACTCTTTGTTTTTTGCTATTAAGGCATTTAATTAGTTGGGACCGAGTTGAATCATTTGC

AATAAATTAATTCTACAATTTTTTCCTAAGTTACCTAAAAGAGAATATATTCTATCAGAT

AAATTTTAAGCACATATAACCAAAAAAAAAAAAAAAATCTAGCAATTGGACCATAAAATT

TCTTCGCATTCGAGAGGGAAAAAAATGGAAAATCTTATCTCCAAGGGATGAAACCCGTAC

GAGGAAGACTGCGAGATTTTCTTATGGTGATTTCCATAAATGACATGACATACAGAAAGA

AGGTAATTAACTAACACATCAAGGTTAATAATTTTATCATCTAGAGAAATTTCATGTTTC

TAGGTCGTTTTGGGACCACAGAACTGACAACTCAAGATAAGCTAATCATATTCTGCAAAT

CAAGGCCCATTTCCTTTGTTTTATTTTTTAAGTTGATTTTCTCGGCAAGCAAACAGACCG

CTTACCAGTTTCTGTATTCAAATTCAATGTACGTATATTTCAAGAAAACTACTCCGAATC

TCCGATGATGATATTTATTTTTTAGACATACGTTAAAGTGAATTTAAGATCGGCGGCGGA

AAGAAAAGGAACGAAAATTAAGGATTTCTGACTTGGGCAAGATACTCGACGACGTAATCC

ATCTTTCTTGTTTGGCAACCGAGAAAATGCAGGAAAGAATAAGAAATTCAAAAATATTCT

AGTTCATGAGATTGTTACTCCGACGAAAGAATTAAACGATCGGCTAAAACTTTGTAAGAC

GCGATAAACTTGAGATCTAAGACGAACTCGTCTTTCTTCCTCAACTTTTCCCGGCAACCA

GCAGATATCTCAGCACATCACCGGATTCACCGACCCAAAAAAAAAAAAAAGGGAAAACGC

AAACAGAGAATCCAAGAAAGAAAGCCTCCAAATGAGAACCGAACTGACCTGCGCAAATGC

CTGATTCCTGATGCTCCGGCCGGAAAAATAAAAGAAAAAGGAGTAAAATGGATTAAATAA

AATCTACAACGCGAGTTGTTGTTGTTTTTGCAGAAAATAATCAAAACTGGAGAATAGAGG

AGAAGAAGAAGAAGAGAACTGTAGTCTCTGTAAAATCTATTAAAGCTTCCTTCTAAGCTT

CAATGAAATCTCTATTATCTCGCTTTGTTTTCTCTACAATGAAGCTATATATATACATAT

ATGTATATGTGTGTATGTACGCCAAAGTTAACTTTTGTCTTGCCAATTTTTTAAATATTA

TTAGGGATAATTAAGCTTTAGTCCCAAAAAGAAAATTTAATTGTATATTAATCTTTATAA

TTTTAGGAATTATACTTTTATCTCTTATGTCTGTTAAAA

>NITAB04G1638 CHR4B 28804196 28808788

ATAATATAGCCTCTCAAAAAAGACTTCCTAACTTTTAATCAAAAACACGCTTAGATTCAG

TTTGCTTAACATTGAGCTGCTCTTTGTCGGTTTAACAACATACATTTTATTTTAACTAAA

GCGAAAACATTCAAAGCAACTAATCATACACAAGATAATGTATAATTATTGATGATACAT

AGCCCAAAAGAAAGACAAATACAGAGTAAGAAGCCCTAAATTAAAGGTGCCAGAAGAAGA

TCTTTTTATGTTACACTTGAATTCACATAAACTCCTTGGGGGAAACAAAAAACAATTCTA

TACTCATTTAGCTCGGGGAGTTGTCAGAACGAATTTCATAATCTTGACAGAGTGAGAGTA

CACAAATCTTCAACAGCAGCAGACGGGCGTGATAAAGCTGAGTAGTGTCTTGATATTGTT

AAGAACTTAATAAAACACCGATTGAGCGCCAAACAATTTCTCTCGACATGTGCAGCAACT

TCAAGGAAGACATCAGATACCAAATGCTATTTGCAATGATTTCTAGACTCGAGCTTGGGA

ATCAAGTAAGAAGCTTGGAAATGCCATGGTTATTTCCCTGGAAAGCAGAAAGCAGAAACC

AGAGTTTAAAATTAAGATTTTAATATACCATTGTATTTCGGCATCAAAATCATAGAAAGG

ATTGAAAAAAAAAAAGTTAGGATCTGATGACTCGAGATTTAGCCAATACTACTGTAAACT

GTCTTTGGTCTTAATGCACGATTAGAATGTTCTACATGATATGTTGAATACTGAAAAGGG

AACAGCTTATAGTAGTGAAAGACATAGAGGTATTGAGAATTATGTAACGTACTTTAGATA

TGGAAGTGTCATGTTTTTAATGATAGTTGGATTCTGTGCCACATACTGCTTCCATTCTTC

CATATCAATTTTTCTATCTCCAGTTAAATCTGCCTCCGACATGGTCTGTCAAATAAGAGC

ACAAGCAAATACACTTAGAAATAGAGCAGAATGAATGAAACATACCAAAACATGAGCCGT

GCATAAATTGTCCACACCTTGTCTACAATTGATTCGATGACATCATCAGAAAGTTCTAGT

TCTGATTCACACAATAGAGCCACCACCATCTCCTTCACCTTCCCGAAGATAATTTGTTGA

CAACATGAGTTCGGAGTATCAGATTTGCAGATATTGTATTATCATAGATAATGGAAGTTT

GAGAATGAAAAAAAAAAAAAAAAAAAAAAAAAAAAGGAGCTTGGATCTAAATTTCTTATT

TTAGTGACATCCACTATTCTATAACCAATTGGAGTTATTAACTTAAAATATCTTAAGAAA

ATAGTATGTAGAAGGGCAATAAGTGAAGTTGCAAGGTATTCACCTCATTATGCTCAATGA

AGCCAGTCTGCCGAAGATCGTACAATCTAAATGCGACTGCAAGAAAAAAAAATTCTAGCT

TAGGATCATGCATACATTTTGACAGATATTATAATAATAGAGCCAATATAGAAGGACATC

AGTTTCTATTAATCATACAGGCAATTTTGTCTTCCACGGGAGTATTAGGATGGAAGACAC

TTAGTGCCCGAACAAATTCTCCGAATTCAATTACCCCATTGTGCTTGACGTCAAACAGGT

CAAATACCTGCAATAACATGGCATGACTATGATAAATATTATTTAGTATAGCATAAGCTA

TTAGAAGCTAGACAAAACGATCACTGCAGAGAATAATAATCCACGACTAACTATAACAAT

TTTCCAATTAAACTGATTAACTGCAGCAACTTCGGCTCATCTTCGAACAAAGTAAATTGC

AATAACTTTTGCACATAGCACACGCTTAGGGCATCACAATACCGCAGGATGATCCAACAA

AGCTACAGAAAATATATTTTTCATCAATGTAATTCAGCTCCTAGCCTTTTAAAATGTTTA

AAATCATCTAGATGAACTAGACTCCAGAGCAATAAAACAAACCCTGTCCGCAAAAAGATT

CTTCTTCTTGCTGTTCCTAAATAGTGCAAGCTGGAATTCTTCCTGCAAAAAATCATCAAT

ACAAGTTAGCACTTCGAACACAAAATCTAGTAGCAAAGAAAAGAAGCAAGAAACTCCGAC

AAATCTTCGGCCCAAAAAAACATGCTTTCATAGTTCAAGCCAAGTATTCAGATTAACTCA

TCCAAACAATAACAGACACCAATGCAACCAGAGAAAGAAATACCTTGTTAATAAGACCAT

CATTACATATAGAGCCACTGATCTTCTTGAACAAATCATACAATGCTTCTACCTCATTAA

CAGTAACTGCCAAAACATCGCAAGAAATTCTTTAGATACAGAGATATGAAACAATCCTAA

TACAATTGACAGCAGGATGCCCCTGATGCAGTAACATCAATTCATACGTTTTTCTACTTA

CATGGCGTCTCAGAAGCAAGGAGCGAAGGCTTCTCATAGCCACGTCTTCGTCTGTAATTC

TTCATGCACAAGCAGCCCGTAAGAGCATCCATGACTTCAGTACAACAACGTGTTAGCTGA

ATGTTCATACAGATTTTTTCATCTTTATACACCTGTCATGTTCCCAAAACAAAGTTCATG

AATATCAGATTTATGCTTAATAAGATCAGATCCCAATATCTTTGTTAATGCTCCCTAATA

CAATCGTACATCTTACTCTCTTTGTTTGCTCTTGGTCAAAATTGCATATTTCATGTTCTT

TTAGCCATACAGACTATAGTTAAAAAACATGTTTAATCAGATCTAAACAGCCTTCTGCTA

ACCTTAATGTCCCTGTACTGCTCGATATCAGCAAGAGAAATAAGCTCCAGGGAAAGAAAT

TTTACATATAATATCTTCCAATTTATAAAAGGTAAACCTCTCCGTCTTCTCTCTGTTTAC

GAATTGGCAAGAGGTAACCAGTTCTAACCTCACAAATGCAGAATTCCAGAATACTTAATA

TTGTAAACTGAACTGTATCAAAAAAATTTAAACAGATCAGAAAAAAAAATAAAAAATGAA

GGTACCGCCCAGATTACCCTTGTATAACATAACACATGCAAAAAATTCTTGCACACGATA

CAATAATGGAATTAGAGTCCTTTCAATTGGAACAGTTCATTTCTTCATGTCAATCAGACG

GCTCACGAATCATAATACATTATGCAACAATTTTTTTTTAAAGAAAAAAAAAAGAAAGAA

AGAAAGAAAAAAGAAGAAGAGATCAGAGCAAAAAAGTCAACCAAACCGGATTAAACATGA

TAGAGAGACAATGCTCAACGAAGATTGACGGTGCAGCAACAACCAGTAACGACACTCGAA

CAAGAGAAAAAAGTTACAAATATGATAAACCTAATTCATTTTCCTGCTACAGTAAACCCC

CAAGAAAAGAACTAGAAAATAAACCGCCATAAATTCCATCCATAGAAAGAAGAAAAATCA

ACCGAAAAAAAAAAAGCACCAAAACTACACGAAACAGTGTTTCTGACCTCCGTTTCGCCA

ATGAGTTGAAGACGAATCTAGAAGCAAAGTATGTAGAAATAATAATTAAAAAGTTAAAAA

ATCAAGAGCTGTATCGAGTTTTTTAATGGAGGAACTCTGGCTTTGGTAAGCGATGGTGAT

GTGTTAGCAGAGGTTTGCACTTCCTCCCCCTTTAAATTTATTATATCGTCGTTGTCGGCA

CCTCCTTCTCGTGAAAAAAGAAAAGAAAATATAATTTTCAATAGTCTTGCTAAGTCAACA

ACTTTTTTTTTTTTTCCTCTTTGTTCGGTTTGTAGGGATTATCCCGTAAACTTATCCATA

CTACGATTAATTTTTATTTGGATTAAATTACATCTTAATTGAAACTGTACAATAAATTAG

ATCGGATTAACACAACCTAAAATCAATCGGATCCAAATTAGATTGGTCATAGAAAAATTT

GGAGTATGTTACTTTGACCCTTTAATGATATAGTTTATAGAACTAATTAGAGTTGGTCCA

ATAACTACACATGCAAAGAAGGGAAAAAAATTTGGATTGGATGCAACCCTGCTGACCATA

TTAGGGCATGGCCCTACTACTTTAGATCGGATCGGGTCTTAAGACCAAAGTACACTTCTA

CATTTTATTTGATAACATCGTCTATACATAATTAGTAAATCAATAATTCAAATTCTCTCT

TTTATATATAAAAAATAAATAAAATAAAATTATGACATATTTTTAATCCCATTATAAATT

TGTACATGATAAAGACATATTTTCTTATATCAGTATATTTTTTTTAATATCTGTTACGGG

TAAAAATATCTCATCCGAGTTGGCGAAACACATGGCAAAGCACGTCACTAAGGAAGGCCC

ACCGGGACCCACGTCAGCAAGCTGGACAAAGGAGGAGTGAAGAAGAGACGCGCCCGGCCT

GGAGAGGCCGAGGAGCTCCCCGGCCTATTTGGCCGAGGTGAGCACCTCGGTCCCAGGCCG

AGGCGCACCTCGACCAAACGGCCAGGTGAGACCTCGGCCATTGGCCGAGGAGTAGCCTTG

GCCATTGGCCGAGGACTTCTCGGCCAATGGCTCCTCGGCCCGTGGCCGAGGTAGCTCCTC

GGTCACGAGCCGAGTCGAGTGACCTCGCCCCAT

>NITAB05G0933 CHR5B 26867197 26872434

TATATGAATATCATGGTGCACTAAAATTATATACATATAACACTACTAATAAATAATAAA

AAAAAATTCCAAAAAAAAAAAAATTAAGAATAAAAATAAAATCACAAATTATAAATTATA

ACCGCAGTGTCTATTAGACTTATAATTTCACTTGAAATGTAATAGTTAAATTCTCATTGA

AAATACGTTACGGTTTTGATGAAATTCAAACTTACATAGAAATTAGTTATATTACAAACA

GTCAAATTTCAATATCCAAATGAAGAAAAAAATCAATCAATAATTAAAGTGGGAAGATAC

GTCTCATCCTTGCTGATCTAATACGGTTGCCACACATAATAATACACATAAATACAAATA

AAAAATAAAATAAAATAATTAGTAATCCACCATGAATCATTCACAAAGTTAAATAATCAA

TCATTTAAACATTTAACGTCAACCTTATATACTTAACCTAAAGGTAAGTGTCTTTGTCAT

TGCGAATTAAAGAGCGAAATAATCATTATTAACAAACCATAGAAATTAATAAATAAATAT

ATTAAATATTGTAATTATACACGTGATTTGTCAAAATTACCCCCGGACGTAATCAATATA

TGGATGATTAGGATGAGGATTCGGATTTAAGATAACTTCTACGGCTTTTATTTTCTACGA

AACAGGAAACCCAAAATGAAAACCAAAATGGGAACTCCGGTTTTATCTATTTAATATCCT

TTTTTTTTTTTAAATTTATTGTAAAAAAAGAAAAATATATTGATTTATTTTCTTTTTTTG

ATGTTTATCTGAAAAACAATTTTAAAACGAAAATTGGGTGACCAGCCACGTGTCCGTTGT

TTTTACTTGTCTGGACCGACCTCTTCCCAGCTCACGAATGAAAAAGCCAGGTCAATTTTG

GGATAAAAATCAGAAGACCACCATTAGCACATGGCTACCTACATCCCTGTGTATACAAGT

TTTAGTTTTCTCGCTTGAGATTTTTTTTTTTTTTTTCCTTTCGATTTATAAGCTTCATTT

CTTGATTTTCTTCTGACATTCACCAGATATAAGGAGTAATTCTATTTACAGAGAGTTTGT

AGGCGAGCTTCCATGAGTGTTGGCCGATTCGTATGGATCCTACTCGCAATTCCGCTATAA

AGCCCCGCACAAGATTCACGTTTCTGGATAACGTATAATTCCTTTCCATTTTTTCTTCTT

TTTTTTCGTTTTTTTTTTTTGTTTTTTTTTTTGTTTTTTTTTTTCCTTTCCTTGATTTTT

GGTGTTTCAATTTATGTTCTGGTCTGAGTTCTTCGATTGATTTTCGTTTCTGTTTGTCGT

GTTCTTTTGAACAGAGTTCGAGTTCCTTAACGCTGGGAGAGAAAATCTTTGCAGGTTGTA

TACCGATAATAGCTATAGTAGAGGCTTTGATTTTTGTTGTGGGTGATTGTTTTGAGCAGC

GGCCCCGACCTCAGAAATGCCGGAAGGATACCGTAGATCTGGTTCAACTCGCCGCAGAAT

CTAGATGTACCGCTCTTTTCTCGCTTTTTCTCGTTTTGGTTTTGGTTTTCCCTTTTTTTT

TTTTTTTTTAATTCATTTACCGTATCATCGTTTCTCGAACACTGATTTTTAATGTGTCTG

GTAGTTGAGAAATCGAGAAAAGCGTTCCAATTAAAATTGAAAAATGGAAAAATATAATAT

GCTTCAGATGGAAGCAAGTTTATTATTGTAAATATAAAAAATTAAGATTGGTTTGTTTAC

TTCTGTATCACATGTAAGGGGAAAAATTAGAAATCTGAAGAACTTATGACATTATTTAAT

GTGCGCCTTTTGTCGGCTGGGATTTTTAATTGTTGTTTTATGGGTAATGGTGCAGTTACG

GTTAATGAACTGGAGGCGTTGTATGAGCTCTATAAGGGCTTGGGTAGCTCCATTATAAAA

GACGGTTTGATTCACAAGGTAATTTGAATATTTCTGCTTTGTTCAATTTGTTGAACTTCT

GATGTAAGTTTTCCATGGTGGCCAGGGAATGTCTTCTGTCTGCCCGAGAACAAAATGTTT

TGACAGCATCCATTTTGAACGTCTCAGTTTGCTCATAGCAAAATTTAAACAGTAAAGAGA

GCCTAGTGATAACATGGTTTACTTGACAATCAAAGAATGAATAATAGACTTTGGGTGCTG

CTTGTTAAGGTTGAGACGTTAAAAGATGCATTATTTTTAAGTGAACCTTATAGTTTCAAC

AATCTGCCCCCTTTCCAATGTCATTCCTGTGTAGGGAATCCCTTCCGGAGATTTACTACA

AGAATATTTTTTTTTTCCTCATTTTATTACTTAATAAGGAATAGAATGAAAATGTCTGTT

CACATGATCAGCTATAATAGTTTTCCTGTGCGGAAAGCATTGGACCTTGCATCCTCTTTT

GAGTGCATCAGCAAAGAGTAGATTTATGGCGTCTCCCGTTGGGCTCCTGACGGAGCTCTA

CCGAAGTCAGTGCTGACTAGCTCTGATTGATGACTACAGCTCTTTTTCAGATATCTTGGA

TTCAGATTAAATCCATGTATTCTACAGTAGTTCTGTTATCAATCGATCTGTCCATGTTTT

TTTCCCATGGATGTTACCTACAGCCTACATTGAGTGGACCGTGCTACATTTTTCATCTTA

TCCATAATCTATTATCTGTATCATGTGCATCCATTGCTTTTGTGTGACATCAACATATCA

GATGTAATGTTGCTCTGTAGGTTCTGTCTATTCCTTGATGTCTTTGTTTGGTCGATAATT

AATTTAGCATTGGTTATATGCTGAATGTATAAGGTCTTGGGCCGGTTGTACTTTATTACA

TTCGACAGTAAGAAATTATCCTTAAAACGTAGTATTAATGCTTACATGTCTCTGCTAAAG

TCATCCTTGTCCATTATGATTGTGCAATGACTTTGTAAGTTAGTTTATGCAAAAGGTAGT

ACTTCTTGCATGTTAACATGGTTATTGTTACTGTTGTTTTTTCTTGTAGGAAGAGCTTCA

GTTAGCATTGTTCCAAACTCCGCATGGTGAAAATCTATTTCTGGATCGGGTAAGCATATA

TGAATAGCCTGGATGTGATTTTATTGATTGTGGCATTGCTTTGCTTTGTTTGATTACTCC

ATGGTGGAAATTTGACATAGACACTGATCGACGTGGAGTTTTCTCATTCAGTGCGGCAAA

TCTGATTCCATCCACGTATCTATCCGGCTACTAATAAGTTTATATCAATATTAAACTTAC

CTTGGTTCTGTACATAATAATGGAATTGTACTTGTTGGTTGGAAAAATTTTATGTTCATA

TCTTCACAATTTTGCATGTATTTACTGATTGTGCTTGCCTTTGCAGGTTTTTGATCTGTT

TGATGAAAAGAAAAATGGTGTCATAGAATTTGAAGAATTTGTTCGTGCACTTAGTGTCTT

CCATCCCTATGCTCCATTGGAAGACAAAATTGATTGTAAGTACTAAAGAAATCATGTAAA

TCATGTCAATTGTATTTAGTTTATGCACATGTTTCATCTATTGATCTTTCCAATTCATTG

CAGTTGCTTTTAGGCTGTATGATCTGAGACAAACTGGGTATATAGAGCGAGAGGAAGTGA

GTGCGCAGAACTTTGATTTAATTATCTGTTCTTGTAGCTGAAATAGATGTTAAAAAACTT

ATTCCTGGCATCGTTCAAGGCTTTAATCTAATTAGCCTGTTTATGATTTCAGGTTAAGCA

AATGGTAATTGCTATTTTGATGGAATCTGACATAAAGTTGTCGGATGACCTTTTGGAAGA

TATAATCGACAAAGTAGGTCATCTCTTTAATCTCTTTGCTTTTAACTTTCTCATTTGTCT

GAAAACATTTCAATACTGATAGCAGCTAAAGCTTGTTGTGCAGACATTTGCGGACGCAGA

TTCCAACAAAGATGGTAAAATTGATAAACAAGAATGGAAGGAGTTTGTTGTTCGACACCC

AACTCTCTTAAAAAACATGACTCTTCCTTATTTAAAGTAAGCATTGCTCTTCCAATATGT

GTGTGCTTTTTACATTCATGACACATGCATGAATTTTCTGATCTATCCTATCATTCATGC

AAATTATGAGGAAATTCTTTAACCCTCTGAAAGAGCATGACTCTTCCGATGTGTGTGTGC

CTTCTACATTCATGACTCGTGCATGATTTTTCTGTTCCTATCCGTCCTATCAATCATACA

TATTATGAGGAAAGTCTTCAATACTCTTCCGATGTGTGTGTGCCTTCTACATTCATGACT

CATGCATGATTTTTCTGTTCCTATCCGTCCTATCAATCATACATATTATGAGGAAAGTCT

TGAATAGGACGGTCTCACATCTTTCAATCAAAAAAGTGCGTGTGGTAACATAAACATTGA

GGTGGCATTTTCTGGTTGAACAGTCCTGCACATGACTGCCTTATGAAAGATTCTCTCCCA

ATCGTTTGCTTTCTTCACCTTTTCACTATATTCCATTGGAAACTGATTATTTTACATTTA

TTACCAACTTCTGCAGAGATATTACGACTATATTCCCAAGTTTTGTTTTCAACACCGCTG

TAGAAGATTGAAATCCAAGGCCCTTTCCAGTATGGCAATTTAACAATTGCAGAGCTGTAT

TGGTTCTGCGTAGAAGTGCTTTCGATCCTTGGCAGAAGATAATACCATGCGTCAGCGGTA

AAGAAGATTGAGGATGCATTCTTACACATTTGGTCACAAGCTCGGCATGAATGGTAACGA

TGCTGAAATCTTTCGTGTTAGACACCCCGTGAACGATTAGCAGGGAATCTCATCGGTCGT

GAGATGCCTGTGCCGTAAAAGTGGACAGGAAGAAAAATATTTTTATCCTCTTCTCTGCCG

AAGAGATTTTTGTAGTCTTATTGCCTTGAATTTGCATGTTAGAGACCCCTGTTGTATGTT

TCCGTATTTGTCTTCTGTCTCCCTGGTGATCAAATGTTGCCGATTATCCAAAGTCTTTTT

CTTTTTTTCTTTCTTTCTGAAATGTGAAAAAGGGTGGTTTGTAAATTAGTAGAAAGTCTC

ACCCCAGAAATTTGCTTCGTTCACCAAATCTCTTATCTTTTCATCCCAATCCAATTGTAT

TGTAGTATTTATACAATACCATCTTCTTCTGCTACTTCTCTTAACTTAGAACCATATTAT

ATCCAATCTCAAAAAGGTCTCATACCCAGTTTTTATATTCTAAGACCTGCTTATCATTTT

ATAAATATTACTTTGATC

>NITAA05G0858 CHR5A 22025869 22030496

CTTTTTGAACACCCCTAGGTGCTTAGATGACTCAATTTTGCTTACTAACCCTAAAGGGTT

GAGATGGTAATCACGATGGATACTTCAGGGGCGACACGTGAAAAGAGATTTGGTCATTTA

GATCAACTCAACAGATTAACGTCATTTTTTTTTTATATGAATATTATGGTGCACTAACAT

TATATACATATAAGTATATAACACTACTAACAAATAAAAAAAAAGTTCCAAAAAACAATA

AAAAATCACGAGTTATAAATTATAACCGCGGTGCCTATTAGGCTTATAATTTTACTTGAA

ATGTAATAGTTGGATTTTCATTGAAAATACATTATGGTTTTGATAAAATTCAAACTTACA

TAGAAATTAGTTATATTACAAACAATCATATTTGAATATTCAATAATTAAGTGGGAAGAT

ACGTCTCATCCAATCGTTGGGTCATTGCTGATCTAATACGATTGCCACACATAATAATAC

ACATAAATACAAATAAAAAAATAAAATAAAATAGTTAGTAATCCACCATATGAATCATTC

ACAAAGTTAAATAATCAATCATTTAAACATTTAACGTCAACCTAATATACTTAACCCAAA

GGTAAGTGTCTTTGTTATTGCGAAATAATCATTATTAACAAACCATAGAAATTAATAAAT

AAATATATTAAATATTGTAATTATACACGTGATTTGTCAAAATTACCCCCGGCCGTAATC

AATATACGGATGATTAGGCCGAGGATTCAGATTTAAGATAACGTCTACGGCTTTTATTTA

TTTATTTATTATTATTATAATTTTTTTTTTGAGAATGCTTTTCTTTACTACGAAACAGGA

AACCAAAATGAGAACTCCGGCATTTAATATTTTTATTTATTTATTGTAAAAAAAGAAAAA

TATATTGATTTATTTTCTGTTTTTGATGTTTATCTGAAAAAACAATTTTAAAACAAAAAT

TGGGTGACCAGCCACGTGTCCGTTGTTTTTACTTGCTAGGACCGACCTCTTCCCAGCTCA

TGAATGAAAAAGCCAGGTCAATTTTGGGATAAAAATCAGAAGACCACCATTAGCACATGG

CTACCTCCATCCCTGTGCATACAAGTTTTGGTTTTCTCGCTTGAGATTTTTTTTTTTTCC

CCTTTTGATTTATAAGCTTCATTTCTTGATTTTCTTCTGACACTCACCAGATATAAGGAG

TAATCCTATTTACAGAGAGTTTGTAGGCGAGCTTCCATGAGTGTTGGCCGATTCGTATGG

ATCCTACTCGCAATTCCGCTTTAAAGCCCCGCACAAGATTCACGTTTCTGGATAACGTAT

AATTCCTCGCCATTTCTTCTCATTTTTTCCTTTTTTTTTCCCCCCTTCCCTTCCTTGATT

TTTGGTGTTTGAATTTATGTTCTGGTCTGAGTTATTCAATTGATTTTCGTTTCTGTTTGG

CGTGTCCTTTGAACAGAGTTCGAGTTCCTTAACGCTCGGAGAGAAAATCTTTGCAGGTTG

TATACCGATAATAGCTATAGTAGAGGCTTTGATTTTTGTTGTGGGTGATTGTTTTGAGCA

ACGGCCTCGACCTCAGAAATGCCGGGAGGATACCGTAGATCTGGTTCAACTGGCCGCAGA

ATCTAGATGTACCGCTCTTTTCTCGCCTTTTCTCGTTTTGGTTTTGGTTTTCCCTTTTCT

TTATTCATTTACCGTATCATCGTTTCTCGGACACTGATTTTTAATGTGTCTGGTAGTTGA

GAAATCGAGAAAAGCGTTCCAATTAAAATTGAAAAATGGAAAAATATAATATGCTTCAGA

TGGAAGCAAGTTCATTATTGTAAATATAAAAAATTAAGATTGGTTTGTTTACTTCTGTAT

CATATGTAAGGGGAAAAATTAGAAACCTGAAGAACTTATGACATTATTTAATGTGCGTCT

TTTGTCGCTGGGATTTTTAATTGTTGTTTTATGGGTAATGGTGCAGTTACCGTTAATGAA

CTGGAGGCGTTGTATGAGCTCTACAAGGGCTTGGGTAGCTCCATTATAAAAGACGGTTTG

ATTCACAAGGTAATTTAAATATTTTTGCTTTGTTCAATTTGTTGAACTTCTGATGTAAGT

TTTCCATGGTGGCCAGGGCATGTCTCCTATCTGCCCGAGAACCATTTTGTTTTAACTAAG

GTCTCAGTTTGCCCATAAGAAAATTTAAACAGGTAAAGAGAGCCTGTTATCGCTTGGCTT

ACTTGACAATCAAAGAAGGAATAATAAAATTAGGGCGCTGCTTGTTAAGGTTGAGGCATC

AACTGCCCCCTTTCCAATGTCATTCCTCTGTAGGGAATCCCTTCTGGAGATTTACCACAA

GAATAGTTTTTTTCTCAATTTGTTGCTTATTGAGGAATAGAATGAAAATCTCTGTTCACA

TGATCAGCTATAAGAGTATGCCTGTGCAGAAAGCATTGGACCTTGCATCCTCTTGTAAGT

GCATCAGCAAAGAGTAGATTTATGGTGTCTCCCGTTGGGCTCCTGACGGAGCTCTACTGA

AGTCAGTGCTGACTAGCTCTGATTGATGACGAGTAGGAATGATATCATATTTGGCACGTG

AAATTTTACAACTCTTTTTCAGATATCTTGGATTCAGATTAAATCCATGTCTTCTACTGT

GATTCTGTTATCAATGAATATGCCCTTGTTTTTACCCCATGGATGTTACCTACAAACCTA

CATTGAGTGGACAATGCTACATTTTTCATTTTATCCATATAATCTATTATCTGTACCATG

TGCACCCATTGCTTCTGTGTGACATCAACATATTAGATGTAATGTCGCTTTTTAGGTTCT

GTCTATCCATTGATGTCTTTGTTTGGTGGATAATTACTTTAGCATTGATTATCCTTAAAA

CATAGTATTAATGCTTAAATGTCTCTACTAAAGTCACCCTTGTCCATTATGATTGTGCAA

TGGCTTTGTAAGTTAATTTATGCAAAAGGTAGTACTTTTTGCATATTAACATGGTTATTG

TTATTGTTGTTTTTTTCTTGTAGGAAGAGCTTCAGTTAGCATTGTTCCAAACTCCGCATG

GTGAAAATCTATTTCTCGATCGGGTAAGCATATATTAATAGCCTGTGATGTGATTTTATT

GATTGTGGCATTGCTTTGCTTTGTTTGATTACTCCATGGTGGAATTTGGACGTAGACAAT

TGATTGACATGGAGTTCTCTCATTTGGTGTGGCAAATCTGATTCCATCCACGTATTTATC

CATCTACTAATATGTTTATATCAATATTAAACTTACCTCGGTGCTGTACCTAATAATGGA

AGTGTACTACTACTTGTTGGTTGGAAAAAGTGTTCATATTTTACAATTTTGCATGTATTT

TATTGGTCGTCTTTGCCTTTGCAGGTTTTTGATCTCTTTGATGAAAAGAAAAATGGTGTC

ATAGAATTTGAAGAATTCGTTCGTGCACTTAGTGTCTTCCATCCCTATGCTCCATTGGAA

GACAAAATTGATTGTAAGCACGAAAAGGAATCATGTAAATCATGTTAATCGTATTTAGTT

CTTGCACATGTTTCATCTGTTGACCTTTCCACTTCATTGCAGTCGCTTTTAGGCTGTATG

ATCTGAGACAAACTGGGTATATAGAACGCGAGGAGGCGAGTGCACTGAACCTGGATTTAA

TTATCAGTTTTTGTAGGTGAAATAGATGTTAAAAAACTTATTCCTGGCATTTTTCAAGCC

TTTAATGTAATTAGCCTGTTTATGATTTCAGGTTAAGCAAATGGTAATTGCCATTTTGAT

GGAATCTGACATAAAGCTGTCTAATGACCTTTTGGAAGATATAATTGACAAAGTAGGTCA

TCTCTTTAATCTCTTTGCTTTTAACTTTCTCATTTGTCTGAAAACATTTCAATACTGATA

GCAGCTAAAGCTTGTTGTGCAGACATTCGCTGACGCAGATTCCAACAAAGATGGTAAAAT

TGATAAACAAGAATGGAAGGAGTTTGTTGTCCGACACCCAACTCTCCTAAAAAACATGAC

TCTTCCTTATTTAAAGTAAGCATTGCTCTTCCAATATGTGTGCGCTTTTTACATTCATGA

CGCATGCATGAAGTTTCTTATCCTATCCTATCATTCATGCAAATTATGAGGAAATTCTTT

AACCCTCTGAAAGAACATGACTCTTCCGATATGTGTGTGCCTTCTACTTTCATGAGTCAT

GCATTATTTTTCTGTTCCTATCCGTCCCATCATTCATATATATTATGAGCAAAGTCTTTG

ATAGGACGGTCTCACATCTTTCAATCAAAAAAGTGTGTATGGTAACATAAACATTGTGGT

GGCATTTTCTGGTTGAACAGTCCTGCACATGACTGCCTTATGAAACATTCTCTCCCAATC

GTTTGCTTTCTTCACCTTTTCACTATATTCCATTGGAAACTGATTATTTTACATTTATTA

CCAACTTCTCCAGAGATATTACGACTATATTCCCAAGTTTTGTTTTCAACACCGCTGTAG

AGGATTGAAATCCAAGGCCCTTTCCAGTATGGCAATTTAACAATTGCAGAGCTGTATTGG

TTCTGCTTAGAAGTGCTTTCGATCCTTGGCAGAAGATAATACCATGCGTCAGCAGTATAG

AAGATTGA

**CBL_CDS.fasta**

>NITAA04G1075

ATGGGGTGTTTTCAGTCAAAGGTAGCGAGGCAGCACCCTGGGTATGAGGACCCAGTTGTT

CTGGCTTCGCAAACTGCGTTTAGTGTTAGTGAAGTTGAAGCCTTGTTTGAGCTATTCAAG

AGCATTAGCGGTTCCCTCATTGACGATGGGTTAATAAGCAAGGAAGAGTTTCAGTTGGCT

CTCTTCAAAAACAGAAAGAAGGAAAATCTATTTGCCAACAGGCTCTTTGAACTTTTTGAT

GTGAAGCGAAAGGGGCTCATTGATTTTAGTGATTTTGTTAGATCACTAAATGTTTTTCAT

CCAAATGCTTCTCAAGAGGACAAGATTGACTTCTCATTTAGGCTATATGATCAAGATAAT

ACAGGATACATCGAGCGTGAAGAGGTCAAGCAAATGCTGATTGCACTTCTATGTGAATCT

GAAATGAAGCTGGCTGATGAAACGATTGAAGTAATACTCGATAAGACTTTCTTGGAAGCC

GATGCAAACCAGGATGGAAAAATAGATAAAACAGAATGGCAAAACTTTGTCTCTAAAAAC

CCATCTTTGTTGAAGATCATGACTCTTCCTTATTTGAGGGACATAACGACGACTTTTCCC

AGTTTTGTTTTTAATTCGGAAGTGGATGAACTTGCCACATAA

>NITAB04G1197

ATGGGGTGTTTTCAGTCAAAGGTAGCAAGGCAGCATCCTGGGTATGAGGACCCAGTTGTT

CTGGCTTCGCAAACTGCGTTTAGTGTTAGTGAAGTTGAAGCCTTGTTTGAGCTATTCAAG

AGCATTAGCGGTTCTCTCATTGACGATGGGTTAATAAGCAAGGAAGAGTTTCAGTTGGCT

CTCTTCAAAAACAGAAAGAAGGAAAATCTATTTGCCAACAGGCTCTTTGAACTTTTTGAT

GTGAAGCGAAAGGGGCTCATTGATTTTAGTGATTTTGTTAGGTCACTAAATGTTTTTCAT

CCAAATGCTTCTCAAGAGGACAAGATTGACTTCTCATTTAGGCTATATGATCAAGATAAT

ACAGGATATATCGAGCGTGAAGAGGTCAAGCAAATGCTGATTGCACTTCTATGTGAATCC

GAAATGAAGCTGGCTGATGAAACGATTGAAGTAATACTCGATAAGACTTTCTTGGAAGCC

GATGCAAACCAGGATGGCAAAATAGATAAAACAGAATGGCAGAACTTTGTTTCTAAAAAC

CCATCTTTGTTGAAGATCATGACCCTTCCTTATTTGAGGGACATAACGACGACTTTTCCC

AGTTTTGTTTTTAATTCAGAAGTGGATGAGCTTGCCACATAA

>NITAB02G1010

ATGTTGCAGTGCGTAGAGGGATTTAAGCATTTATTTGCTTCCCTATTGCAGTGCTGTGAT

CTTGATTTGTACAAACAATCAAGGGGCCTTGAAGATCCCGAAGTTCTTGCTAGAGAGACA

GTTTTGGATACTGTTGTTATATATTACTCCTGCTTAATTTTGTCCTTCAAAAACCCTGAG

GAGTTCCAATTGGCGTTGTTTAAGACGAACAAAAAAGAGAGCTTGTTTGCAGACCGGGTC

TTTGACTTGTTTGATACAAAGCACAATGGAATTCTGGGTTTTGAAGAGTTTGCTCGTGCT

CTCTCTGTCTTCCATCCAAATGCTCCCATTGATGATAAAATTGAGTTTTCTTTTCAGCTA

TACGATCTCAAGCAACAGGGTTTTATCGAGAGACAGGAGGTGAAGCAAATGGTGGTAGCT

ACACTTGCTGAGTCGGGTATGAACCTCTCAGATGATGTTATAGAAAGTATAATTGACAAG

ACTTTTGAGGAAGCTGATACAAAACATGACGGGAAGATTGACAAGGAAGAATGGAGAAAC

CTTGTCCTGAGACATCCATCTCTTCTCAAGAATATGACTCTTCAATACCTTAACGCATTA

AACAACATGAACAAAGTTATAAGGATTGATGTTATTATAACTAATGTCATATTGTCGTTA

GACCATAATTTAGCTTTTGATGGGACATCACCACAACTTTCCCAAGTTTTGTATTCCATT

CACGAGTTGAAGATACCTGAACTCAAAATATGCAGATGA

>NITAA02G0832

ATGGTGCAGTGCATAGAGGGATTTAAGCATTTATTTGCTTCCCTATTGCAGTGCTGTGAT

CTTGATTTGTACAAACAATCAAGGGGCCTTGAAGATCCTGAAGTTCTTGCTAGAGAGACA

GTTTTTAGTGTAAGTGAAATTGAAGCACTTTATGAGCTGTTTAAGAAGATCAGCAGCGCC

GTGATCGATGACGGGCTGATTAACAAGGAGGAGTTCCAATTGGCGTTGTTTAAGACGAAC

AAAAAAGAGAGCTTGTTTGCAGATCGGGTCTTTGACTTGTTTGATACGAAGCACAATGGA

ATTCTGGGTTTTGAAGAGTTTGCTCGAGCTCTCTCTGTCTTCCATCCAAATGCTCCCATT

GATGATAAAATTGAGTTTTCTTTTCAGCTATACGATCTCAAGCAACAGGGTTTTATTGAG

AGACAGGAGGTGAAGCAAATGGTGGTAGCTACACTTGCTGAGTCGGGTATGAACCTCTCA

GATGATGTTATAGAAAGTATAATTGACAAGACTTTTGAGGAAGCTGATACAAAACATGAT

GGGAAGATTGACAAGGAAGAATGGAGAAACCTTGTCCTGCGACATCCATCTCTTCTCAAG

AATATGACTCTTCAATACCTTAAGGACATCACCACAACTTTCCCAAGTTTTGTATTCCAT

TCACGAGTTGAAGATACCTGA

>NITAA02G2027

ATGGGTTGTAAGTGCTCGAAGATAAGGAAACAAACACTTGGTTATGAGGATCCTACCGTT

CTTGCTGCTGTAACACCTTTTACAGTTAGTGAAGTAGAAGCCTTGCATGAGCTTTTCAAG

AAATTAAGCAATTCAATATTCCATGATGGGCTCATTCACAAGGAAGAATTCCAATTTGCA

CTCTTTAGAAACAGAAGAAAGAAGAATCTTTTTGCTGACAGGATTTTTGATCTTTTTGAT

GCCAAACGCAATGGGGTTATTGAGTTTGGGGAATTCGTTCGATCCTTGGGAGTATTCCAT

CCAAATGCGCGTGTTGAGGATAAAATAGCATTTGCTTTTAGATTGTATGATTTAAGACAA

ACCGGCTTTATCGAGCGGGAGGAGTTGAGGGAGATGGTATTGGCGCTTTTGCATGAATCA

GATTTAGTACTCTCAGATGAATACATTGAAACTATTGTGGATCAGACATTCAAAGAAGCT

GATACTAAAGGTGATGGAAAAATTGACCCAGAAGAATGGAAGGAGTTTGTCTCGAAGCAT

CCTTCTCTAATAAAGAACATGACTCTTCCATATTTAAAGGATATAACCTTGGCATTTCCA

AGCTTTGTTCTATCTTCTGAAGTTGAGGATTCAGATGTGTAG

>NITAA02G2024

ATGGGTTGTAAGTGCTCGAAGATAAGGAAACAAACACTTGGTTATGAGGATCCTACCGTT

CTTGCTGCTGTAACACCTTTTACAGTTAGTGAAGTAGAAGCCTTGCATGAGCTTTTCAAG

AAATTAAGCAATTCAATATTCCATGATGGGCTCATTCACAAGGAAGAATTCCAATTTGCA

CTCTTTAGAAACAGAAGAAAGAAGAATCTTTTTGCTGACAGGATTTTTGATCTTTTTGAT

GCCAAACGCAATGGGGTTATTGAGTTTGGGGAATTCGTTCGATCCTTGGGAGTATTCCAT

CCAAATGCGCGTGTTGAGGATAAAATAGCATTTGCTTTTAGATTGTATGATTTAAGACAA

ACCGGCTTTATCGAGCGGGAGGAGTTGAGGGAGATGGTATTGGCGCTTTTGCATGAATCA

GATTTAGTACTCTCAGATGAATACATTGAAACTATTGTGGATCAGACATTCAAAGAAGCT

GATACTAAAGGTGATGAAAAAATTGACCCAGAAGAATGGAAGGAGTTTGTCTCGAAGCAT

CCTTCTCTAATAAAGAACATGACTCTTCCATATTTAAAGGATATAACCTTGGCATTTCCA

AGCTTTGTTCTATCTTCTGAAGTTGAGGATTCAGATGTGTAG

>NITAB02G2334

ATGGGTTGTAAGTGGTCGAAGATAAGGAAACAAACACTTGGTTTTGAGGATCCTACCGTT

CTTGCTGCTGTAACACCTTTTACAGTTAGTGAAGTAGAAGCCTTGCATGAGCTTTTCAAG

AAATTAAGCAATTCAATATTCCATGATGGGCTCATTCACAAGGAAGAATTCCATTTTGCA

CTCTTTAGAAACAGAAGAAAGAAGAATCTATTTGCTGACAGGATTTTTGATCTTTTTGAT

GCCAAACGCAATGGGGTTATTGAGTTTGGGGAATTCGTCCGATCCCTGGGAGTATTCCAT

CCAAATGCGCGTGTTGAGGATAAAATAGCATTTGCTTTTAGATTGTATGATTTAAGACAA

ACCGGCTTTATCGAGCAAGAGGAGTTGAGGGAGATGGTTTTGGCGCTTTTGCATGAATCA

GATTTAGTACTCTCAGATGAATACATTGAAACTATTGTGGATCAGACATTCAAAGAAGCT

GATACTAAAGGTGATGGAAAAATTGACCCAGAAGAATGGAAGGAGTTTGTCTCGAAGCAT

CCTTCTCTAATAAAGAACATGACTCTTCCATATTTAAAGGATATAACCTTGGCATTTCCA

AGCTTTGTTCTATCTTCTGAAGTTGAGGATTCAGATGTGTAG

>NITAB04G1638

ATGGGGCGAGGTCACTCGACTCGGCTCGTGACCGAGGAGCTACCTCGGCCACGGGCCGAG

GAGCCATTGGCCGAGAAGTCCTCGGCCAATGGCCAAGGCTACTCCTCGGCCAATGGCCGA

GGTCTCACCTGGCCGTTTGGTCGAGTTCAGTTTACAATATTAAGTATTCTGGAATTCTGC

ATTTGTGAGATATTATATGTAAAATTTCTTTCCCTGGAGCTTATTTCTCTTGCTGATATC

GAGCAGTACAGGGACATTAAGGTGTATAAAGATGAAAAAATCTGTATGAACATTCAGCTA

ACACGTTGTTGTACTGAAGTCATGGATGCTCTTACGGGCTGCTTGTGCATGAAGAATTAC

AGACGAAGACGTGGCTATGAGAAGCCTTCGCTCCTTGCTTCTGAGACGCCATTTACTGTT

AATGAGGTAGAAGCATTGTATGATTTGTTCAAGAAGATCAGTGGCTCTATATGTAATGAT

GGTCTTATTAACAAGGAAGAATTCCAGCTTGCACTATTTAGGAACAGCAAGAAGAAGAAT

CTTTTTGCGGACAGGGTATTTGACCTGTTTGACGTCAAGCACAATGGGGTAATTGAATTC

GGAGAATTTGTTCGGGCACTAAGTGTCTTCCATCCTAATACTCCCGTGGAAGACAAAATT

GCCTTCGCATTTAGATTGTACGATCTTCGGCAGACTGGCTTCATTGAGCATAATGAGGTG

AAGGAGATGGTGGTGGCTCTATTGTGTGAATCAGAACTAGAACTTTCTGATGATGTCATC

GAATCAATTGTAGACAAGACCATGTCGGAGGCAGATTTAACTGGAGATAGAAAAATTGAT

ATGGAAGAATGGAAGCAGTATGTGGCACAGAATCCAACTATCATTAAAAACATGACACTT

CCATATCTAAAGGAAATAACCATGGCATTTCCAAGCTTCTTACTTGATTCCCAAGCTCGA

GTCTAG

>NITAA04G1516

ATGAACATTCAGCTAACACGTTGTTGTACTGAAGTCATGGATGCTCTTACGGACTGCTTG

TGCATGAAGAATTACAGACGAAGACGTGGCTATGAGAAGCCTTCGCGCCTTGCTTCTGAG

ACACCATTCACTGTTAATGAAGTAGAAGCACTGTATGATTTGTTCAAGAAGATCAGTGGT

TCTATATGTGATGATGGTCTTATTAACAAGGAAGAATTCCAGCTTGCACTATTTAGGAAC

AGCAAGAAGAAGAATCTTTTTGCGGACAGGGTATTTGACCTGTTTGACGTCAAGCGCAAT

GGGGTAATTGAATTCGGAGAATTTGTTCGGGCACTAAGTGTCTTCCATCCTAATACTCCC

GTGGAAGACAAAATTGCCTTCGCATTTAGATTGTATGATCTTCGGCAGACTGGCTTCATT

GAGCATAATGAGGTGAAGGAGATGGTGGTGGCTCTATTGTGTGAATCAGAACTGGAACTT

TCGGATGATTGCATCGAATCAATTGTAGACAAGACTATGTCGGAGGCAGATTTAACTGGA

GATAGAAAAATTGATATGGAAGAATGGAAGCAGTATGTGGCACAGAATCCAACTATCATT

AAAAACATGACACTTCCATATCTAAAGGAAATAACTATGGCATTTCCAAGCTTCTTACTT

GATTCCCAAGCTCGAGTCTAG

>NITAB05G0933

ATGGATCCTACTCGCAATTCCGCTATAAAGCCCCGCACAAGATTCACGTTTCTGGATAAC

AGTTCGAGTTCCTTAACGCTGGGAGAGAAAATCTTTGCAGGTTGTATACCGATAATAGCT

ATAGTAGAGGCTTTGATTTTTGTTGTGGGTGATTGTTTTGAGCAGCGGCCCCGACCTCAG

AAATGCCGGAAGGATACCGTAGATCTGGTTCAACTCGCCGCAGAATCTAGATTTACGGTT

AATGAACTGGAGGCGTTGTATGAGCTCTATAAGGGCTTGGGTAGCTCCATTATAAAAGAC

GGTTTGATTCACAAGGAAGAGCTTCAGTTAGCATTGTTCCAAACTCCGCATGGTGAAAAT

CTATTTCTGGATCGGGTTTTTGATCTGTTTGATGAAAAGAAAAATGGTGTCATAGAATTT

GAAGAATTTGTTCGTGCACTTAGTGTCTTCCATCCCTATGCTCCATTGGAAGACAAAATT

GATTTTGCTTTTAGGCTGTATGATCTGAGACAAACTGGGTATATAGAGCGAGAGGAAGTT

AAGCAAATGGTAATTGCTATTTTGATGGAATCTGACATAAAGTTGTCGGATGACCTTTTG

GAAGATATAATCGACAAACTAAAGCTTGTTGTGCAGACATTTGCGGACGCAGATTCCAAC

AAAGATGGTAAAATTGATAAACAAGAATGGAAGGAGTTTGTTGTTCGACACCCAACTCTC

TTAAAAAACATGACTCTTCCTTATTTAAAAGATATTACGACTATATTCCCAAGTTTTGTT

TTCAACACCGCTGTAGAAGATTGA

>NITAA05G0858

ATGGATCCTACTCGCAATTCCGCTTTAAAGCCCCGCACAAGATTCACGTTTCTGGATAAC

AGTTCGAGTTCCTTAACGCTCGGAGAGAAAATCTTTGCAGGTTGTATACCGATAATAGCT

ATAGTAGAGGCTTTGATTTTTGTTGTGGGTGATTGTTTTGAGCAACGGCCTCGACCTCAG

AAATGCCGGGAGGATACCGTAGATCTGGTTCAACTGGCCGCAGAATCTAGATTTACCGTT

AATGAACTGGAGGCGTTGTATGAGCTCTACAAGGGCTTGGGTAGCTCCATTATAAAAGAC

GGTTTGATTCACAAGGAAGAGCTTCAGTTAGCATTGTTCCAAACTCCGCATGGTGAAAAT

CTATTTCTCGATCGGGTTTTTGATCTCTTTGATGAAAAGAAAAATGGTGTCATAGAATTT

GAAGAATTCGTTCGTGCACTTAGTGTCTTCCATCCCTATGCTCCATTGGAAGACAAAATT

GATTTCGCTTTTAGGCTGTATGATCTGAGACAAACTGGGTATATAGAACGCGAGGAGGTT

AAGCAAATGGTAATTGCCATTTTGATGGAATCTGACATAAAGCTGTCTAATGACCTTTTG

GAAGATATAATTGACAAACTAAAGCTTGTTGTGCAGACATTCGCTGACGCAGATTCCAAC

AAAGATGGTAAAATTGATAAACAAGAATGGAAGGAGTTTGTTGTCCGACACCCAACTCTC

CTAAAAAACATGACTCTTCCTTATTTAAAAGCTGTATTGGTTCTGCTTAGAAGTGCTTTC

GATCCTTGGCAGAAGATAATACCATGCGTCAGCAGTATAGAAGATTGA

**CBL_gff**

CHR4A maker mRNA 21576777 21581709 . - . ID=NITAA04G1075;old_ID=Maker00017996;_AED=0.06;_eAED=0.06;_QI=942|1|1|1|0.87|0.77|9|1186|213;

CHR4A maker three_prime_UTR 21576777 21577962 . - . Parent=NITAA04G1075;old_Parent=Maker00017996;

CHR4A maker CDS 21577963 21578026 . - 1 Parent=NITAA04G1075;old_Parent=Maker00017996;

CHR4A maker CDS 21578118 21578230 . - 0 Parent=NITAA04G1075;old_Parent=Maker00017996;

CHR4A maker CDS 21578740 21578820 . - 0 Parent=NITAA04G1075;old_Parent=Maker00017996;

CHR4A maker CDS 21578902 21578954 . - 2 Parent=NITAA04G1075;old_Parent=Maker00017996;

CHR4A maker CDS 21579047 21579155 . - 0 Parent=NITAA04G1075;old_Parent=Maker00017996;

CHR4A maker CDS 21579248 21579307 . - 0 Parent=NITAA04G1075;old_Parent=Maker00017996;

CHR4A maker CDS 21580018 21580100 . - 2 Parent=NITAA04G1075;old_Parent=Maker00017996;

CHR4A maker CDS 21580505 21580583 . - 0 Parent=NITAA04G1075;old_Parent=Maker00017996;

CHR4A maker five_prime_UTR 21580584 21580700 . - . Parent=NITAA04G1075;old_Parent=Maker00017996;

CHR4A maker five_prime_UTR 21580885 21581709 . - . Parent=NITAA04G1075;old_Parent=Maker00017996;

CHR4B maker mRNA 24367647 24372487 . - . ID=NITAB04G1197;old_ID=Maker00040437;_AED=0.08;_eAED=0.08;_QI=939|1|1|1|0.87|0.77|9|1197|213;

CHR4B maker five_prime_UTR 24371666 24372487 . - . Parent=NITAB04G1197;old_Parent=Maker00040437;

CHR4B maker five_prime_UTR 24371365 24371481 . - . Parent=NITAB04G1197;old_Parent=Maker00040437;

CHR4B maker CDS 24371286 24371364 . - 0 Parent=NITAB04G1197;old_Parent=Maker00040437;

CHR4B maker CDS 24370813 24370895 . - 2 Parent=NITAB04G1197;old_Parent=Maker00040437;

CHR4B maker CDS 24370129 24370188 . - 0 Parent=NITAB04G1197;old_Parent=Maker00040437;

CHR4B maker CDS 24369928 24370036 . - 0 Parent=NITAB04G1197;old_Parent=Maker00040437;

CHR4B maker CDS 24369783 24369835 . - 2 Parent=NITAB04G1197;old_Parent=Maker00040437;

CHR4B maker CDS 24369621 24369701 . - 0 Parent=NITAB04G1197;old_Parent=Maker00040437;

CHR4B maker CDS 24369001 24369113 . - 0 Parent=NITAB04G1197;old_Parent=Maker00040437;

CHR4B maker CDS 24368844 24368907 . - 1 Parent=NITAB04G1197;old_Parent=Maker00040437;

CHR4B maker three_prime_UTR 24367647 24368843 . - . Parent=NITAB04G1197;old_Parent=Maker00040437;

CHR2B maker mRNA 15519064 15523183 . + . ID=NITAB02G1010;old_ID=Maker00038721;_AED=0.18;_eAED=0.18;_QI=294|0.6|0.81|0.81|0.4|0.54|11|713|252;

CHR2B maker five_prime_UTR 15519064 15519199 . + . Parent=NITAB02G1010;old_Parent=Maker00038721;

CHR2B maker five_prime_UTR 15520195 15520321 . + . Parent=NITAB02G1010;old_Parent=Maker00038721;

CHR2B maker five_prime_UTR 15520408 15520438 . + . Parent=NITAB02G1010;old_Parent=Maker00038721;

CHR2B maker CDS 15520439 15520562 . + 0 Parent=NITAB02G1010;old_Parent=Maker00038721;

CHR2B maker CDS 15520734 15520786 . + 2 Parent=NITAB02G1010;old_Parent=Maker00038721;

CHR2B maker CDS 15521136 15521195 . + 0 Parent=NITAB02G1010;old_Parent=Maker00038721;

CHR2B maker CDS 15521323 15521431 . + 0 Parent=NITAB02G1010;old_Parent=Maker00038721;

CHR2B maker CDS 15521595 15521647 . + 2 Parent=NITAB02G1010;old_Parent=Maker00038721;

CHR2B maker CDS 15521749 15521829 . + 0 Parent=NITAB02G1010;old_Parent=Maker00038721;

CHR2B maker CDS 15521949 15522061 . + 0 Parent=NITAB02G1010;old_Parent=Maker00038721;

CHR2B maker CDS 15522241 15522329 . + 1 Parent=NITAB02G1010;old_Parent=Maker00038721;

CHR2B maker CDS 15522394 15522470 . + 2 Parent=NITAB02G1010;old_Parent=Maker00038721;

CHR2B maker three_prime_UTR 15522471 15523183 . + . Parent=NITAB02G1010;old_Parent=Maker00038721;

CHR2A maker mRNA 14740891 14744844 . + . ID=NITAA02G0832;old_ID=Maker00031407;_AED=0.14;_eAED=0.14;_QI=297|1|1|1|0.63|0.58|12|551|226;

CHR2A maker five_prime_UTR 14740891 14741027 . + . Parent=NITAA02G0832;old_Parent=Maker00031407;

CHR2A maker five_prime_UTR 14742012 14742140 . + . Parent=NITAA02G0832;old_Parent=Maker00031407;

CHR2A maker five_prime_UTR 14742227 14742257 . + . Parent=NITAA02G0832;old_Parent=Maker00031407;

CHR2A maker CDS 14742258 14742381 . + 0 Parent=NITAA02G0832;old_Parent=Maker00031407;

CHR2A maker CDS 14742678 14742760 . + 2 Parent=NITAA02G0832;old_Parent=Maker00031407;

CHR2A maker CDS 14742923 14742982 . + 0 Parent=NITAA02G0832;old_Parent=Maker00031407;

CHR2A maker CDS 14743113 14743221 . + 0 Parent=NITAA02G0832;old_Parent=Maker00031407;

CHR2A maker CDS 14743385 14743437 . + 2 Parent=NITAA02G0832;old_Parent=Maker00031407;

CHR2A maker CDS 14743539 14743619 . + 0 Parent=NITAA02G0832;old_Parent=Maker00031407;

CHR2A maker CDS 14743739 14743851 . + 0 Parent=NITAA02G0832;old_Parent=Maker00031407;

CHR2A maker CDS 14744183 14744240 . + 1 Parent=NITAA02G0832;old_Parent=Maker00031407;

CHR2A maker three_prime_UTR 14744241 14744332 . + . Parent=NITAA02G0832;old_Parent=Maker00031407;

CHR2A maker three_prime_UTR 14744359 14744793 . + . Parent=NITAA02G0832;old_Parent=Maker00031407;

CHR2A maker three_prime_UTR 14744821 14744844 . + . Parent=NITAA02G0832;old_Parent=Maker00031407;

CHR2A maker mRNA 41041387 41047466 . - . ID=NITAA02G2027;old_ID=Maker00033264;_AED=0.09;_eAED=0.09;_QI=479|1|1|1|0.77|0.6|10|643|213;

CHR2A maker three_prime_UTR 41041387 41041990 . - . Parent=NITAA02G2027;old_Parent=Maker00033264;

CHR2A maker three_prime_UTR 41042011 41042049 . - . Parent=NITAA02G2027;old_Parent=Maker00033264;

CHR2A maker CDS 41042050 41042113 . - 1 Parent=NITAA02G2027;old_Parent=Maker00033264;

CHR2A maker CDS 41042335 41042447 . - 0 Parent=NITAA02G2027;old_Parent=Maker00033264;

CHR2A maker CDS 41042535 41042615 . - 0 Parent=NITAA02G2027;old_Parent=Maker00033264;

CHR2A maker CDS 41042888 41042940 . - 2 Parent=NITAA02G2027;old_Parent=Maker00033264;

CHR2A maker CDS 41043063 41043171 . - 0 Parent=NITAA02G2027;old_Parent=Maker00033264;

CHR2A maker CDS 41043287 41043346 . - 0 Parent=NITAA02G2027;old_Parent=Maker00033264;

CHR2A maker CDS 41043453 41043535 . - 2 Parent=NITAA02G2027;old_Parent=Maker00033264;

CHR2A maker CDS 41044465 41044543 . - 0 Parent=NITAA02G2027;old_Parent=Maker00033264;

CHR2A maker five_prime_UTR 41044544 41044605 . - . Parent=NITAA02G2027;old_Parent=Maker00033264;

CHR2A maker five_prime_UTR 41047050 41047466 . - . Parent=NITAA02G2027;old_Parent=Maker00033264;

CHR2A maker mRNA 40867594 40873675 . - . ID=NITAA02G2024;old_ID=Maker00008686;_AED=0.08;_eAED=0.08;_QI=479|1|1|1|0.77|0.6|10|643|213;

CHR2A maker three_prime_UTR 40867594 40868197 . - . Parent=NITAA02G2024;old_Parent=Maker00008686;

CHR2A maker three_prime_UTR 40868218 40868256 . - . Parent=NITAA02G2024;old_Parent=Maker00008686;

CHR2A maker CDS 40868257 40868320 . - 1 Parent=NITAA02G2024;old_Parent=Maker00008686;

CHR2A maker CDS 40868542 40868654 . - 0 Parent=NITAA02G2024;old_Parent=Maker00008686;

CHR2A maker CDS 40868742 40868822 . - 0 Parent=NITAA02G2024;old_Parent=Maker00008686;

CHR2A maker CDS 40869094 40869146 . - 2 Parent=NITAA02G2024;old_Parent=Maker00008686;

CHR2A maker CDS 40869269 40869377 . - 0 Parent=NITAA02G2024;old_Parent=Maker00008686;

CHR2A maker CDS 40869493 40869552 . - 0 Parent=NITAA02G2024;old_Parent=Maker00008686;

CHR2A maker CDS 40869660 40869742 . - 2 Parent=NITAA02G2024;old_Parent=Maker00008686;

CHR2A maker CDS 40870674 40870752 . - 0 Parent=NITAA02G2024;old_Parent=Maker00008686;

CHR2A maker five_prime_UTR 40870753 40870814 . - . Parent=NITAA02G2024;old_Parent=Maker00008686;

CHR2A maker five_prime_UTR 40873259 40873675 . - . Parent=NITAA02G2024;old_Parent=Maker00008686;

CHR2B maker mRNA 45889259 45895357 . - . ID=NITAB02G2334;old_ID=Maker00034795;_AED=0.11;_eAED=0.11;_QI=473|1|1|1|0.87|0.77|9|639|213;

CHR2B maker five_prime_UTR 45894947 45895357 . - . Parent=NITAB02G2334;old_Parent=Maker00034795;

CHR2B maker five_prime_UTR 45892407 45892468 . - . Parent=NITAB02G2334;old_Parent=Maker00034795;

CHR2B maker CDS 45892328 45892406 . - 0 Parent=NITAB02G2334;old_Parent=Maker00034795;

CHR2B maker CDS 45891291 45891373 . - 2 Parent=NITAB02G2334;old_Parent=Maker00034795;

CHR2B maker CDS 45891125 45891184 . - 0 Parent=NITAB02G2334;old_Parent=Maker00034795;

CHR2B maker CDS 45890900 45891008 . - 0 Parent=NITAB02G2334;old_Parent=Maker00034795;

CHR2B maker CDS 45890724 45890776 . - 2 Parent=NITAB02G2334;old_Parent=Maker00034795;

CHR2B maker CDS 45890377 45890457 . - 0 Parent=NITAB02G2334;old_Parent=Maker00034795;

CHR2B maker CDS 45890177 45890289 . - 0 Parent=NITAB02G2334;old_Parent=Maker00034795;

CHR2B maker CDS 45889898 45889961 . - 1 Parent=NITAB02G2334;old_Parent=Maker00034795;

CHR2B maker three_prime_UTR 45889259 45889897 . - . Parent=NITAB02G2334;old_Parent=Maker00034795;

CHR4B maker mRNA 28804196 28808788 . - . ID=NITAB04G1638;old_ID=Maker00036507;_AED=0.30;_eAED=0.30;_QI=0|0.8|0.72|0.90|0.9|1|11|522|321;

CHR4B maker CDS 28808644 28808788 . - 0 Parent=NITAB04G1638;old_Parent=Maker00036507;

CHR4B maker CDS 28807104 28807147 . - 2 Parent=NITAB04G1638;old_Parent=Maker00036507;

CHR4B maker CDS 28806958 28807029 . - 0 Parent=NITAB04G1638;old_Parent=Maker00036507;

CHR4B maker CDS 28806597 28806747 . - 0 Parent=NITAB04G1638;old_Parent=Maker00036507;

CHR4B maker CDS 28806399 28806481 . - 2 Parent=NITAB04G1638;old_Parent=Maker00036507;

CHR4B maker CDS 28806158 28806217 . - 0 Parent=NITAB04G1638;old_Parent=Maker00036507;

CHR4B maker CDS 28805714 28805822 . - 0 Parent=NITAB04G1638;old_Parent=Maker00036507;

CHR4B maker CDS 28805559 28805611 . - 2 Parent=NITAB04G1638;old_Parent=Maker00036507;

CHR4B maker CDS 28805233 28805313 . - 0 Parent=NITAB04G1638;old_Parent=Maker00036507;

CHR4B maker CDS 28805028 28805140 . - 0 Parent=NITAB04G1638;old_Parent=Maker00036507;

CHR4B maker CDS 28804718 28804772 . - 1 Parent=NITAB04G1638;old_Parent=Maker00036507;

CHR4B maker three_prime_UTR 28804196 28804717 . - . Parent=NITAB04G1638;old_Parent=Maker00036507;

CHR4A maker mRNA 26046762 26050429 . - . ID=NITAA04G1516;old_ID=Maker00017851;_AED=0.19;_eAED=0.19;_QI=427|1|1|1|0.77|0.7|10|535|226;

CHR4A maker three_prime_UTR 26046762 26047296 . - . Parent=NITAA04G1516;old_Parent=Maker00017851;

CHR4A maker CDS 26047297 26047351 . - 1 Parent=NITAA04G1516;old_Parent=Maker00017851;

CHR4A maker CDS 26047601 26047713 . - 0 Parent=NITAA04G1516;old_Parent=Maker00017851;

CHR4A maker CDS 26047805 26047885 . - 0 Parent=NITAA04G1516;old_Parent=Maker00017851;

CHR4A maker CDS 26048123 26048175 . - 2 Parent=NITAA04G1516;old_Parent=Maker00017851;

CHR4A maker CDS 26048277 26048385 . - 0 Parent=NITAA04G1516;old_Parent=Maker00017851;

CHR4A maker CDS 26048719 26048778 . - 0 Parent=NITAA04G1516;old_Parent=Maker00017851;

CHR4A maker CDS 26048960 26049042 . - 2 Parent=NITAA04G1516;old_Parent=Maker00017851;

CHR4A maker CDS 26049158 26049284 . - 0 Parent=NITAA04G1516;old_Parent=Maker00017851;

CHR4A maker five_prime_UTR 26049285 26049308 . - . Parent=NITAA04G1516;old_Parent=Maker00017851;

CHR4A maker five_prime_UTR 26049499 26049698 . - . Parent=NITAA04G1516;old_Parent=Maker00017851;

CHR4A maker five_prime_UTR 26050227 26050429 . - . Parent=NITAA04G1516;old_Parent=Maker00017851;

CHR5B maker mRNA 26867197 26872434 . + . ID=NITAB05G0933;old_ID=Maker00034236;_AED=0.04;_eAED=0.04;_QI=1112|0.87|0.88|1|0.87|0.88|9|667|267;

CHR5B maker three_prime_UTR 26871768 26872434 . + . Parent=NITAB05G0933;old_Parent=Maker00034236;

CHR5B maker CDS 26871713 26871767 . + 1 Parent=NITAB05G0933;old_Parent=Maker00034236;

CHR5B maker CDS 26871062 26871192 . + 0 Parent=NITAB05G0933;old_Parent=Maker00034236;

CHR5B maker CDS 26870909 26870989 . + 0 Parent=NITAB05G0933;old_Parent=Maker00034236;

CHR5B maker CDS 26870740 26870792 . + 2 Parent=NITAB05G0933;old_Parent=Maker00034236;

CHR5B maker CDS 26870543 26870651 . + 0 Parent=NITAB05G0933;old_Parent=Maker00034236;

CHR5B maker CDS 26870186 26870245 . + 0 Parent=NITAB05G0933;old_Parent=Maker00034236;

CHR5B maker CDS 26869052 26869134 . + 2 Parent=NITAB05G0933;old_Parent=Maker00034236;

CHR5B maker CDS 26868531 26868702 . + 0 Parent=NITAB05G0933;old_Parent=Maker00034236;

CHR5B maker CDS 26868309 26868368 . + 0 Parent=NITAB05G0933;old_Parent=Maker00034236;

CHR5B maker five_prime_UTR 26867197 26868308 . + . Parent=NITAB05G0933;old_Parent=Maker00034236;

CHR5A maker mRNA 22025869 22030496 . + . ID=NITAA05G0858;old_ID=Maker00006096;_AED=0.16;_eAED=0.20;_QI=931|0.77|0.8|1|0.66|0.5|10|0|275;

CHR5A maker five_prime_UTR 22025869 22026381 . + . Parent=NITAA05G0858;old_Parent=Maker00006096;

CHR5A maker five_prime_UTR 22026707 22027124 . + . Parent=NITAA05G0858;old_Parent=Maker00006096;

CHR5A maker CDS 22027125 22027184 . + 0 Parent=NITAA05G0858;old_Parent=Maker00006096;

CHR5A maker CDS 22027325 22027496 . + 0 Parent=NITAA05G0858;old_Parent=Maker00006096;

CHR5A maker CDS 22027835 22027917 . + 2 Parent=NITAA05G0858;old_Parent=Maker00006096;

CHR5A maker CDS 22028892 22028951 . + 0 Parent=NITAA05G0858;old_Parent=Maker00006096;

CHR5A maker CDS 22029253 22029361 . + 0 Parent=NITAA05G0858;old_Parent=Maker00006096;

CHR5A maker CDS 22029451 22029503 . + 2 Parent=NITAA05G0858;old_Parent=Maker00006096;

CHR5A maker CDS 22029620 22029700 . + 0 Parent=NITAA05G0858;old_Parent=Maker00006096;

CHR5A maker CDS 22029773 22029903 . + 0 Parent=NITAA05G0858;old_Parent=Maker00006096;

CHR5A maker CDS 22030418 22030496 . + 1 Parent=NITAA05G0858;old_Parent=Maker00006096;
