# Supplementary material for: Genome-wide discovery of CBL genes in Nitraria tangutorum Bobr. and functional analysis of NtCBL1-1 under drought and salt stress
Source: For Res (Fayettev). 2023 Dec 22;3:28. doi: 10.48130/FR-2023-0028 (PMC11524306; doi:10.48130/FR-2023-0028)
Supplement: Supplementary file 1 — Supplementary data to this article can be found online. [file FR-2023-0028-S1.zip › 10.48130_FR-2023-0028-Suppl-TableS1.pdf]

Table S1. Primers for Quantitative RT-PCR in *N.tangutorum*

Primer	Sequence(5'-3')
Nt_actin-F	GGAATCCACGAGACCACCTACA
Nt_actin-R	GATTGATCCTCCGATCCAGACA
q-NtCBL1-F	AGCGTGAAGAGGTCAAGCAA
q-NtCBL1-R	ACCTGGTTTGCATCGGCTT
q-NtCBL3-F	AGCTACACTTGCTGAGTCGG
q-NtCBL3-R	ATGGATGTCGCAGGACAAGG
q-NtCBL4-F	GATCCTACCGTTCTTGCTGCT
q-NtCBL4-R	AGGATCGAACGAATTCCCCA
q-NtCBL8-F	AGGAGATGGTGGTGGCTCTAT
q-NtCBL8-R	TCTGTGCCACATACTGCTTCC
q-NtCBL10-F	TGCCGGAAGGATACCGTAGA
q-NtCBL10-R	ACCATGCGGAGTTTGGAACA
