# Supplementary material for: Genome-wide discovery of CBL genes in Nitraria tangutorum Bobr. and functional analysis of NtCBL1-1 under drought and salt stress
Source: For Res (Fayettev). 2023 Dec 22;3:28. doi: 10.48130/FR-2023-0028 (PMC11524306; doi:10.48130/FR-2023-0028)
Supplement: Supplementary file 1 — Supplementary data to this article can be found online. [file FR-2023-0028-S1.zip › 10.48130_FR-2023-0028-Suppl-TableS2.pdf]

Table S2. Primers for isolation of *NtCBL1-1* fragment and construction of overexpression vector

Use	Primer	Sequence(5'-3')
Gene clone	NtCBL1F	ATGGGGTGTTTTCAGTCAAA
	NtCBL1R	TTATGTGGCAAGCTCATCCA
Overexpression vector	NtCBL1-1-121-F	gaacacgggggactctagaggatccccggggATGGGGTGTTTTCAGTCAAAGG
	NtCBL1-1-121-R	ttgaacgatcggggaaattcgagctcTGTGGCAAGCTCATCCACTTCT
