# Supplementary material for: Genome-wide discovery of CBL genes in Nitraria tangutorum Bobr. and functional analysis of NtCBL1-1 under drought and salt stress
Source: For Res (Fayettev). 2023 Dec 22;3:28. doi: 10.48130/FR-2023-0028 (PMC11524306; doi:10.48130/FR-2023-0028)
Supplement: Supplementary file 1 — Supplementary data to this article can be found online. [file FR-2023-0028-S1.zip › 10.48130_FR-2023-0028-Suppl-TableS3.pdf]

Table S3. CBL genes used to construct phylogenetic trees from 12 species except *N.tangutorum*

Arabidopsis thaliana	Gene name	Gene ID
	AtCBL1	AT4G17615
	AtCBL2	AT5G55990
	AtCBL3	AT4G26570
	AtCBL4	AT5G24270
	AtCBL5	AT4G01420
	AtCBL6	AT4G16350
	AtCBL7	AT4G26560
	AtCBL8	AT1G64480
	AtCBL9	AT5G47100
	AtCBL10	AT4G33000
Oryza sativa	OsCBL1	LOC_Os10g41510
	OsCBL2	LOC_Os12g40510
	OsCBL3	LOC_Os03g42840
	OsCBL4	LOC_Os05g45810
	OsCBL5	LOC_Os01g41510
	OsCBL6	LOC_Os12g06510
	OsCBL7	LOC_Os02g18880
	OsCBL8	LOC_Os02g18930
	OsCBL9	LOC_Os01g39770
	OsCBL10	LOC_Os01g51420
Citrus clementina	CcCBL1	Ciclev10022219m
	CcCBL2	Ciclev10016575m
	CcCBL3	Ciclev10009412m
	CcCBL4	Ciclev10022148m
	CcCBL8	Ciclev10023612m
	CcCBL10-1	Ciclev10009236m
	CcCBL10-2	Ciclev10026324m
Eucalyptus grandis	EgCBL1	Eucgr.D02136
	EgCBL2	Eucgr.F03674
	EgCBL3	Eucgr.E03829
	EgCBL4-1	Eucgr.A01457
	EgCBL4-2	Eucgr.K00375
	EgCBL4-3	Eucgr.K00377
	EgCBL4-4	Eucgr.K00380
	EgCBL4-5	Eucgr.E00051
	EgCBL8	Eucgr.D01757
	EgCBL9	Eucgr.E00335
	EgCBL10-1	Eucgr.F03125
	EgCBL10-2	Eucgr.C00642
Picea abies	PaCBL1	MA_9965g0020
	PaCBL2	MA_27386g0010
	PaCBL3	MA_10431324g0020
	PaCBL4	MA_105065g0010
	PaCBL5	MA_10204459g0010
	PaCBL6	MA_269415g0010
	PaCBL7	MA_3268g0010
	PaCBL8	MA_7799814g0010
	PaCBL9	MA_9445g0010
	PaCBL10	MA_18112g0010
	PaCBL11	MA_10280648g0010
	PaCBL12	MA_184231g0010
	PaCBL13	MA_10288665g0010
Populous trichocarpa	PtCBL2-1	Potri.006G002900
	PtCBL2-2	Potri.016G003500
	PtCBL2-3	Potri.001G371700
	PtCBL3	Potri.011G094900
	PtCBL4-1	Potri.015G013100
	PtCBL4-2	Potri.012G015100
	PtCBL4-3	Potri.015G013200
	PtCBL5	Potri.003G141400
	PtCBL8	Potri.001G090200
	PtCBL9	Potri.001G150200
	PtCBL10	Potri.006G230200

Prunus persica	PperCBL1	ppa011375m.g
	PperCBL3	ppa011040m.g
	PperCBL4-1	ppa011404m.g
	PperCBL4-2	ppa016322m.g
	PperCBL5	ppa014557m.g
	PperCBL8	ppa020531m.g
	PperCBL10	ppa010315m.g
Theobroma cacao	TcCBL1	Thecc1EG015835
	TcCBL2	Thecc1EG030465
	TcCBL3	Thecc1EG030580
	TcCBL4	Thecc1EG015411
	TcCBL5	Thecc1EG016487
	TcCBL8	Thecc1EG016488
	TcCBL10	Thecc1EG037279
Vitis venifer	VvCBL1-1	GSVIVG01004840001
	VvCBL1-2	GSVIVG01004842001
	VvCBL3-1	GSVIVG01004678001
	VvCBL3-2	GSVIVG01014744001
	VvCBL4	GSVIVG01038549001
	VvCBL5	GSVIVG01019554001
	VvCBL8	GSVIVG01019555001
	VvCBL10-1	GSVIVG01035369001
	VvCBL10-2	GSVIVG01035370001
Citrus sinensis	CsCBL1	orange1.1g040252m
	CsCBL2	orange1.1g027239m
	CsCBL3	orange1.1g027336m
	CsCBL4	orange1.1g027657m
	CsCBL5	orange1.1g041619m
	CsCBL8	orange1.1g048069m
	CsCBL10-1	orange1.1g025162m
	CsCBL10-2	orange1.1g025241m
Malus domestica	MdCBL1-1	MDP0000207134
	MdCBL1-2	MDP0000262916
	MdCBL2	MDP0000119547
	MdCBL3	MDP0000137075
	MdCBL4-1	MDP0000155124
	MdCBL4-2	MDP0000774066
	MdCBL4-3	MDP0000263313
	MdCBL5	MDP0000294682
	MdCBL8	MDP0000256696
	MdCBL9	MDP0000259502
	MdCBL10	MDP0000186655
Selaginella moellendorffii	SmCBL2	409467
	SmCBL3	272115
	SmCBL5	81220
	SmCBL9	131200
