# Supplementary material for: Genome-wide discovery of CBL genes in Nitraria tangutorum Bobr. and functional analysis of NtCBL1-1 under drought and salt stress
Source: For Res (Fayettev). 2023 Dec 22;3:28. doi: 10.48130/FR-2023-0028 (PMC11524306; doi:10.48130/FR-2023-0028)
Supplement: Supplementary file 1 — Supplementary data to this article can be found online. [file FR-2023-0028-S1.zip › 10.48130_FR-2023-0028-Suppl-TableS4.pdf]

Table S4 KaKs analysis in *N.tangutorum*

raw seq pairs name	gene id	Ka	Ks	Ka/Ks
NITAA05G0858-NITAB05G0933	NtCBL10-1--NtCBL10-2	0.031357703	0.154229737	0.203318135
NITAA04G1516-NITAB04G1638	NtCBL8-1--NtCBL8-2	0.011423353	0.07043295	0.162187623
NITAA02G0832-NITAB02G1010	NtCBL3-1--NtCBL3-2	0.10095412	0.223121871	0.452461786
NITAA04G1075-NITAB04G1197	NtCBL1-1--NtCBL1-2	0	0.09430325	0
NITAA02G2027-NITAB02G2334	NtCBL4-1--NtCBL4-3	0.008017447	0.037297927	0.214956913
NITAA02G2027-NITAA02G2024	NtCBL4-1--NtCBL4-2	0.001997338	0	NULL
NITAA02G2024-NITAB02G2334	NtCBL4-2--NtCBL4-3	0.010028563	0.03739092	0.268208517
