# Supplementary material for: Genome-wide discovery of CBL genes in Nitraria tangutorum Bobr. and functional analysis of NtCBL1-1 under drought and salt stress
Source: For Res (Fayettev). 2023 Dec 22;3:28. doi: 10.48130/FR-2023-0028 (PMC11524306; doi:10.48130/FR-2023-0028)
Supplement: Supplementary file 1 — Supplementary data to this article can be found online. [file FR-2023-0028-S1.zip › 10.48130_FR-2023-0028-Suppl-TableS5.pdf]

Table S5 Identity between *CBLs* gene in *Nitraria tangutorum*.

Identity（%）	NtCBL1-1	NtCBL1-2	NtCBL3-1	*NtCBL3-2*	NtCBL4-1	NtCBL4-2	NtCBL4-3	NtCBL8-1	NtCBL8-2	NtCBL10-1	NtCBL10-2
NtCBL1-1		**98.13%**	65.11%	66.49%	68.07%	68.07%	68.07%	42.18%	60.00%	67.68%	67.76%
NtCBL1-2	**98.13%**	-	65.60%	67.22%	68.58%	68.41%	68.41%	42.05%	59.71%	67.68%	67.76%
NtCBL3-1	65.11%	65.60%	-	**90.06%**	68.52%	68.28%	68.04%	65.65%	65.18%	67.65%	66.31%
NtCBL3-2	66.49%	67.22%	**90.06%**	-	68.43%	68.25%	67.98%	66.42%	66.24%	66.37%	64.83%
NtCBL4-1	68.24%	68.58%	68.52%	68.43%	-	**99.84%**	**98.60%**	74.13%	74.31%	67.03%	67.69%
NtCBL4-2	68.07%	68.41%	68.28%	68.25%	**99.84%**	-	**98.44%**	73.96%	74.13%	66.85%	67.50%
NtCBL4-3	68.07%	68.41%	68.04%	67.98%	**98.60%**	**98.44%**	-	73.96%	74.13%	67.03%	67.50%
NtCBL8-1	42.18%	42.05%	65.65%	66.42%	74.13%	73.96%	73.96%	-	**97.65%**	68.01%	67.31%
NtCBL8-2	60.00%	59.71%	65.18%	66.24%	74.31%	74.13%	74.13%	**97.65%**	-	68.33%	67.12%
NtCBL10-1	67.68%	67.68%	67.65%	66.37%	67.03%	66.85%	67.03%	68.01%	68.33%	-	**96.80%**
NtCBL10-2	67.76%	67.76%	66.31%	64.83%	67.69%	67.50%	67.50%	67.31%	67.12%	**96.80%**	-
